# Closed‐Loop Recyclable Solid Polymer Electrolytes via Dynamic Lipoate Cross‐Linking

**DOI:** 10.1002/anie.3540036

**Published:** 2026-08-17

**Authors:** Qian Huang, Jeet Sharma, Yingna Ding, Kenny Lee, Craig J. Hawker, Dipan Kundu, Cyrille Boyer

**Affiliations:** ^1^ Cluster for Advanced Macromolecular Design School of Chemical Engineering University of New South Wales Sydney New South Wales Australia; ^2^ School of Chemical Engineering UNSW Sydney Sydney New South Wales Australia; ^3^ Materials Department and Department of Chemistry and Biochemistry University of California Santa Barbara California USA; ^4^ School of Mechanical and Manufacturing Engineering UNSW Sydney Sydney New South Wales Australia

**Keywords:** bio‐sourced monomer, closed‐loop recycling, depolymerization, dynamic disulfide bonds, ionic conductivity, solid polymer electrolyte, supercapacitor, sustainable energy storage

## Abstract

Growing demand for electrochemical energy storage has intensified concerns over battery end‐of‐life disposal, driving an urgent need for recyclable electrolyte materials. Here, we report a photo‐curable, chemically recyclable solid polymer electrolyte platform built on a bio‐derived difunctional cross‐linker, poly(ethylene glycol) bis(lipoate) (PEG‐LP_2_), prepared from naturally occurring α‐lipoic acid. Visible‐light‐driven copolymerization of PEG‐LP_2_ with poly(ethylene glycol) methyl ether acrylate in the presence of an ionic liquid (BMITFSI, 45 wt%) yields flexible, ionically conductive membranes (1.30 mS cm^−1^ at room temperature). The dynamic disulfide cross‐linked network imparts both solid‐like mechanical integrity and chemical recyclability. When assembled into symmetric supercapacitors, the optimized SPE‐65 formulation retains ∼70% of its initial specific capacitance after 6000 galvanostatic cycles at 100 mA g^−1^. Complete catalyst‐assisted depolymerization is achieved using 1,8‐diazabicyclo[5.4.0]undec‐7‐ene (DBU) under mild, ambient conditions, and subsequent repolymerization regenerates ionically conductive membranes retaining ∼70% of the original conductivity (0.86 mS cm^−1^). Together, the bio‐derived lipoate cross‐linker, catalytically assisted depolymerization, and regenerated electrochemical performance constitute a sustainable and recyclable SPE manufacture platform for next‐generation supercapacitor applications.

## Graphical abstract

A visible‐light‐driven, closed‐loop recyclable solid polymer electrolyte is built from a bio‐derived lipoate cross‐linker, poly(ethylene glycol) bis(lipoate) (PEG‐LP_2_), synthesized from α‐lipoic acid. Dynamic disulfide exchange enables efficient DBU‐catalyzed depolymerization and photo‐repolymerization, regenerating membranes that retain high ionic conductivity and specific capacitance in symmetric supercapacitors.

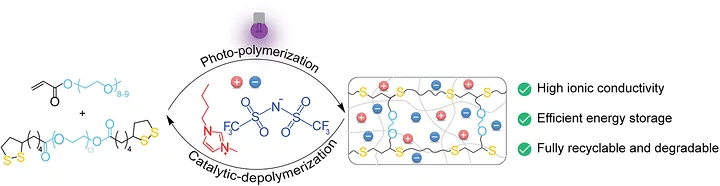

## 1 Introduction

The surging global demand for electrochemical energy storage has driven a sharp rise in the volume of end‐of‐life devices, intensifying the need for recyclable materials [1, 2]. Beyond improving electrochemical performance through advanced composite and nanostructured materials [3, 4], the development of future energy‐storage systems is increasingly shifting toward sustainable materials that integrate safety, durability, self‐healability, degradability, and recyclability [5, 6, 7, 8]. In this context, sustainable and self‐healable polymer electrolyte systems have emerged as particularly attractive research directions, including natural biopolymer‐based electrolytes [9] and dynamic covalent polymer networks [10, 11, 12, 13]. These systems aim not only to improve electrochemical device safety and lifetime by repairing mechanically or electrochemically induced damage, but also to reduce the environmental burden related to electrolyte production, disposal, and recycling [14].

Despite increasing attention to battery recycling, current efforts focus predominantly on electrode materials, which account for only 20%–25% of a battery's total mass [15], whereas electrolyte recovery typically depends on energy‐intensive, high‐temperature processes that pose significant safety hazards and erode the net environmental benefits of recycling [16, 17]. Establishing a genuine circular economy in battery technology therefore demands electrolyte materials that are intrinsically sustainable, combining robust operational stability, high energy density, and safe, efficient end‐of‐life recovery [18, 19].

Solid polymer electrolytes (SPEs) have emerged as a compelling alternative to conventional liquid electrolytes, addressing the latter's flammability, toxicity, and leakage risks while offering improved safety, higher energy density, and enhanced electrochemical stability for next‐generation energy storage [20, 21]. Conventional (meth)acrylate‐based SPEs form chemically robust carbon–carbon cross‐linked networks that deliver excellent durability (Figure 1a), yet their resistance to depolymerization renders material recovery exceedingly difficult [22, 23, 24]. The central challenge, then, is to design SPEs that simultaneously achieve efficient recyclability and robust operational stability, two properties that have historically proven difficult to reconcile. Although recent advances have yielded degradable and recyclable solid‐state electrolytes for lithium‐ion and lithium‐metal batteries, many of these systems remain limited by complex synthesis or demanding processing requirements. For example, Williams and coworkers reported recyclable cyclic carbonate‐based SPEs for lithium metal battery, achieving an ionic conductivity of 0.52 mS cm^−1^ (Figure 1b); however, their depolymerization required elevated temperatures and metal ion catalysts [25]. Similarly, Wang and coworkers employed a similar strategy to develop a high‐performance recyclable SPE with an ionic conductivity of 1.62 mS cm^−1^ (Figure 1c), yet recycling was contingent upon high‐temperature activation [26]. Both approaches highlight the continuing challenge of achieving efficient recycling under mild and practical conditions. Therefore, recyclable polymer electrolytes based on simpler dynamic covalent exchange networks are highly attractive, as they offer synthetic accessibility, processing flexibility, practical scalability, and closed‐loop recyclability [27].

**FIGURE 1:**
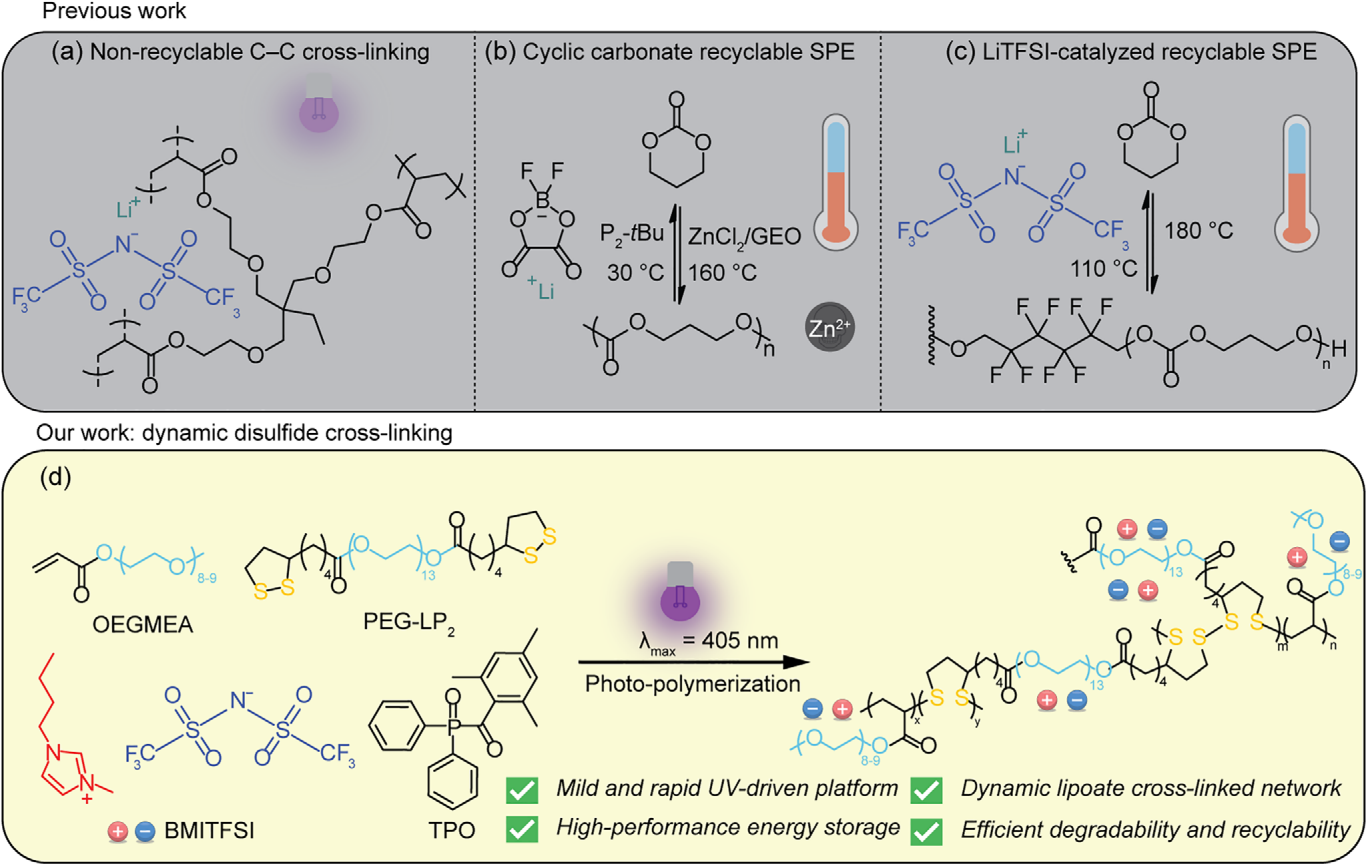
Comparison of selected strategies for polymer electrolytes. (a) Zhao et al. reported a highly cross‐linked gel polymer electrolyte for lithium‐ion batteries, but the non‐degradable acrylate framework prevented recyclability [22]. (b) Williams et al. developed a cyclic carbonate‐based recyclable solid polymer electrolyte for lithium metal batteries, however, with limited ionic conductivity (0.52 mS cm^−1^) and required high temperature and metal ions for depolymerization [25]. (c) Wang et al. demonstrated a closed‐loop recyclable SPE using LiTFSI as catalyst, although the recycling process relied on high‐temperature activation [26]. (d) This work: A mild and rapid visible‐light driven photopolymerization for the synthesis of a recyclable SPE enabled by dynamic disulfide cross‐linking, achieving closed‐loop recyclability and high ionic conductivity (1.30 mS cm^−1^).

Significant progress toward this goal has been made through the incorporation of dynamic covalent motifs into polymer networks, particularly those based on 1,2‐dithiolane compounds, such as α‐lipoic acid (LA) and its derivatives. Critically, α‐lipoic acid is a naturally occurring organosulfur compound biosynthesized in both plants and animals, where it serves as an essential metabolic cofactor; its commercial production relies on renewable feedstocks and fermentation‐based routes, rendering it an attractive bio‐derived monomer for sustainable polymer design [28, 29, 30, 31, 32, 33, 34]. These cyclic disulfide‐containing monomers are uniquely suited for constructing degradable, closed‐loop recyclable networks: polylipoate homopolymers can be efficiently depolymerized via disulfide bond cleavage and subsequently repolymerized through cationic, anionic, or radical ring‐opening polymerization [35, 36, 37, 38]. LA derivatives have also been successfully copolymerized with vinyl monomers such as acrylates via radical polymerization, yielding degradable and recyclable polymer architectures [39, 40, 41, 42]. Unlike purely carbon‐backbone polymers, lipoate‐containing copolymers incorporate disulfide linkages along the main chain, enabling dynamic bond exchange, imparting self‐healing capability, and facilitating depolymerization to thiol‐terminated oligomers that retain the capacity for repolymerization [37, 43, 44]. Collectively, these attributes make LA‐derived networks a highly attractive platform for recyclable SPE design.

Building on these advances, we report a bio‐derived difunctional lipoate cross‐linker, poly(ethylene glycol) bis(lipoate) (PEG‐LP_2_), incorporated into a visible‐light‐initiated radical copolymerization with poly(ethylene glycol) methyl ether acrylate (OEGMEA) in the presence of diphenyl(2,4,6‐trimethylbenzoyl)phosphine oxide (TPO) and the ionic liquid 1‐butyl‐3‐methylimidazolium bis(trifluoromethylsulfonyl)imide (BMITFSI) to generate a disulfide‐containing polymer electrolyte membrane. This approach moves beyond conventional thermoset polymer electrolyte designs by introducing dynamic disulfide cross‐linked networks in an ion‐conducting membrane under a mild and rapid visible light‐driven photopolymerization. By systematically varying the PEG‐LP_2_:OEGMEA molar ratio, we optimized the SPE formulation to achieve a balance between its closed‐loop recyclability and the electrochemical performance in symmetric supercapacitors. This design strategy demonstrates that high ionic conductivity, electrochemical stability, and efficient closed‐loop material recovery are mutually achievable, providing a scalable and practical pathway toward environmentally responsible next‐generation supercapacitor applications.

## 2 Results and Discussion

### 2.1 Design and Synthesis of Dynamic Lipoate Cross‐Linked SPE

To develop these polymer electrolytes that combine high ionic conductivity with chemical recyclability, we designed a photocurable system comprising poly(ethylene glycol) methyl ether acrylate (OEGMEA, *M*_W_ = 480 g/mol), diphenyl(2,4,6‐trimethylbenzoyl)phosphine oxide (TPO), and the bio‐derived difunctional cross‐linker poly(ethylene glycol) bis(lipoate) (PEG‐LP_2_). This cross‐linker contains two lipoate (1,2‐dithiolane) end groups connected by a poly(ethylene glycol) chain with a degree of polymerization of 13. During radical polymerization, the 1,2‐dithiolane groups undergo ring‐opening to generate a cross‐linked network of dynamic disulfide bonds throughout the OEGMEA matrix. As demonstrated in previous studies [45, 46, 47], these bonds can be selectively cleaved by specific catalysts, enabling depolymerization into constituent oligomers and recovery of the bio‐derived cross‐linker for efficient material recovery.

PEG‐LP_2_ was synthesized via esterification between the bio‐derived monomer α‐lipoic acid and a low molecular weight poly(ethylene glycol), utilizing 1‐ethyl‐3‐(3‐(dimethylamino)propyl)carbodiimide (EDC) as the coupling agent and 4‐dimethylaminopyridine (DMAP) as the catalyst. As noted above, α‐lipoic acid is a naturally occurring, commercially available compound produced via biosynthetic routes. Successful formation of PEG‐LP_2_ was confirmed by ^1^H NMR and ^13^C NMR spectroscopy, along with size exclusion chromatography (SEC) analysis (Figures S1–S3). Low molecular weight PEG segments were deliberately selected for both the monomer and cross‐linker to suppress PEG crystallization and preserve high chain segmental mobility—both prerequisites for achieving high ionic conductivity at room temperature [48, 49]. The ionic liquid BMITFSI was incorporated at a fixed loading of 45 wt% across all formulations, exploiting its high intrinsic conductivity and excellent compatibility with PEG‐based matrices [50, 51, 52]. A series of membranes was prepared by systematically varying the molar ratio of OEGMEA to PEG‐LP_2_ from 20:80 to 80:20 mol% at constant BMITFSI content. The resulting membranes are denoted SPE‐*x*, where *x* represents the molar percentage of PEG‐LP_2_ in the formulation; for example, SPE‐65 corresponds to 65 mol% PEG‐LP_2_ and 35 mol% OEGMEA (Table S1).

Prior to membrane fabrication, photopolymerization kinetics were investigated to determine the optimal irradiation time. The kinetics were monitored by tracking vinyl bond conversion using attenuated total reflectance Fourier‐transform infrared (ATR‐FTIR) spectroscopy. Monomer conversion was calculated from the vinyl stretching overtone integral at 1500–1650 cm^−1^, normalized against the carbonyl stretching absorbance at 1660–1780 cm^−1^ as an internal standard. As shown in Figure S4, the vinyl stretching intensity of SPE‐20 decreases markedly after polymerization, indicating near‐complete consumption of the OEGMEA monomer. Figure S5 shows the polymerization kinetics for four formulations (SPE‐20, SPE‐35, SPE‐50, and SPE‐65), demonstrating that increasing PEG‐LP_2_ content progressively reduces the polymerization rate. In addition, the photopolymerization kinetics of SPE‐20 were evaluated under different light intensities and wavelengths (*λ*_max_ = 405 and 530 nm). The 405 nm wavelength was selected to match the characteristic UV–visible absorption band of the TPO photoinitiator (385‐410 nm), ensuring efficient radical generation. In contrast, irradiation at 530 nm did not induce measurable polymerization (Figure S6), consistent with TPO's negligible absorbance at this wavelength. Monomer conversion for SPE‐80 could not be reliably determined due to the relatively low acrylate monomer content. Nevertheless, all other SPE formulations reached conversions above 90% under irradiation in less than 90 s, confirming efficient curing as lipoate conversion is normally higher than the vinyl conversion [53, 54].

Having established the photopolymerization kinetics for all formulations, membranes (thickness = 0.5 mm, diameter = 11 mm) were fabricated by casting the SPE precursor into pre‐cut hollow silicone molds and irradiating with a violet light LED array (*λ*_max_ = 405 nm and *I*_0_ = 2.06 mW cm^−2^) using TPO as photoinitiator for 180 s (Figure 1d), yielding transparent, flexible membranes. The resulting membranes were used directly for thermal, mechanical, and electrochemical characterization without further purification.

To identify the viscoelastic behavior of the resulting membranes, dynamic mechanical analysis (DMA) was performed from −70°C to 30°C to measure their glass transition temperature (*T*_g_) and storage modulus (*G*'). The *T*_g_ at approximately −50°C and a long plateau from −10°C to 30°C among all samples suggested sufficient polymer segmental mobility of lipoate‐based dynamic cross‐linked SPEs, facilitating ion transport (Figure S7). Meanwhile, the retained storage modulus above *T*_g_ confirmed that the membranes maintain solid‐like mechanical integrity under ambient conditions (Figure S8). Therefore, the DMA results demonstrate a balance between segmental mobility for ionic conduction and sufficient mechanical stability (*G'* ≈1 MPa) at room temperature for further solid‐state energy‐storage applications. Additionally, the thermal stability of the recyclable membranes was further confirmed by thermogravimetric analysis (TGA). The membranes remained thermally stable up to approximately 270°C. The TGA and derivative thermogravimetric curves of all SPE samples exhibited two main thermal degradation stages, at approximately 270°C–370°C and 370°C–490°C, which can be assigned to the decomposition of the dynamic disulfide‐cross‐linked polymer network and the ionic liquid component, respectively (Figure S9).

Building on this favorable thermomechanical behavior, electrochemical impedance spectroscopy (EIS) was conducted to measure the ionic conductivity of the resulting SPEs. Ionic conductivity increased from 0.28 to 1.30 mS cm^−1^ as the molar ratio of PEG‐LP_2_ increased from 20 to 65 mol%. Beyond 65 mol%, however, ionic conductivity dropped markedly from 1.30 to 0.57 mS cm^−1^ as the PEG‐LP_2_ content went from 65 to 80 mol% (Figure 2a). Additional temperature‐dependent EIS measurements were performed to study ionic conductivity at elevated temperatures. The conductivity of SPE‐65 increased steadily from 25°C to 60°C, reaching 3.87 mS cm^−1^ at 60°C (Figure S11). This enhanced ionic conductivity at elevated temperatures showcased the suitability of the recyclable SPEs for electrochemical energy‐storage applications across a broad operating temperature range.

**FIGURE 2:**
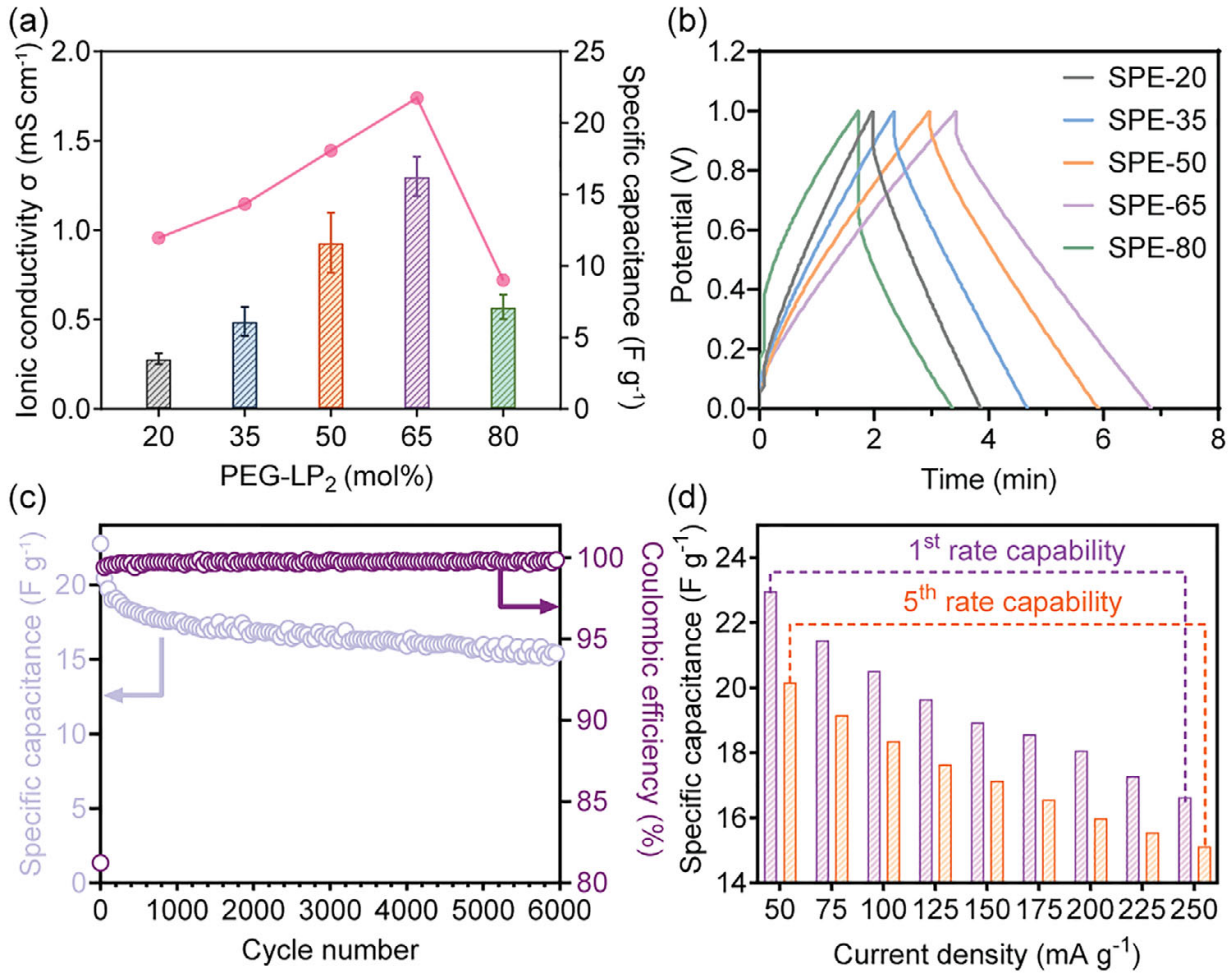
(a) Ionic conductivity (bars) and specific discharge capacitance (line) of SPE formulations as a function of the PEG‐LP_2_:OEGMEA molar ratio. The specific discharge capacitance was measured at 100 mA g^−1^ current density. (b) Galvanostatic charge‐discharge (GCD) plots of symmetric AC supercapacitor/SPE at 100 mA g^−1^ current density, cycled in 1 V operating voltage window. (c) Galvanostatic charge–discharge (GCD) profiles of symmetric AC||SPE‐65||AC supercapacitor at 100 mA g^−1^ within a 1 V operating voltage window. (d) Rate capability of symmetric AC||SPE‐65||AC supercapacitor at current densities ramping from 50 to 250 mA g^−1^. *Note*: EIS analysis performed in triplicates; error bars represent the standard deviation obtained from these repeated measurements.

Having acquired high ionic conductivity for photocured SPEs, galvanostatic charge‐discharge (GCD) analysis was performed on symmetric activated carbon (AC) supercapacitors assembled with each SPE formulation. Each device was assembled by sandwiching the SPE membrane between AC electrodes with graphite foil current collectors (thickness = 50 µm) (Section S2.10). The GCD results mirrored the ionic conductivity trend: SPE‐65 delivered the highest specific capacitance, while SPE‐80 showed the lowest (Figure 2b). The specific capacitance of the AC||SPE‐65||AC supercapacitor exhibited reasonable stability over extended cycling, retaining 15.0 F g^−1^ (65.8% of its initial value of 22.8 F g^−1^) after 6000 cycles (Figure 2c). To evaluate the electrochemical durability of SPE‐65, open‐circuit voltage (OCV) decay and floating/holding tests were performed on symmetric AC||SPE‐65||AC supercapacitor (Figures S14–S17). During the OCV decay measurements, measurable voltage decay was observed, with the cell stabilizing at approximately 0.65 V under open‐circuit conditions, indicating low self‐discharge. While in the floating/holding voltage test, the charged supercapacitor was held at 1.0 V for 2 h during each cycle, exhibiting minimal leakage current of approximately 75 µA in the first cycle to only ∼50 µA, indicating minimal parasitic reactions. Furthermore, the consistently low leakage‐current over successive floating cycles, together with the consistent Nyquist spectra and preserved galvanostatic charge‐discharge characteristics demonstrate stable interfacial kinetics and low overall electrochemical degradation. These results, in combination, confirmed the outstanding electrochemical stability and performance of symmetric AC||SPE‐65||AC system. To further assess rate capability of the AC||SPE‐65||AC device under high‐current operation, galvanostatic charge‐discharge experiments were conducted over current densities ranging from 50 to 250 mA g^−1^ (Figure 2d and Figure S12). Each current density was maintained for 50 cycles, resulting in 450 cycles per loop. Five such loops were completed for symmetric AC||SPE‐65||AC device. Specific capacitance recycled to near its initial value after each loop, with approximately 88% retention over five complete loops. Cyclic voltammetry over a 0–2 V window at scan rates from 1 to 100 mV s^−1^ was also performed to characterize AC||SPE‐65||AC (Figure S13). Collectively, these electrochemical measurements confirmed the exceptional operational stability and high‐capacity retention of assembled AC||SPE‐65||AC electrochemical cell. A current density of 100 mA g^−1^ was also used for GCD analysis among all symmetric supercapacitors assembled with other different SPE formulations. All these devices exhibited > 98% Coulombic efficiency over 2000 cycles (Figures S18–S21).

### 2.2 Depolymerization and Recyclability

The incorporation of dynamic disulfide bonds into SPEs provides a robust mechanism for degradation, facilitating both recyclability and reusability (Scheme 1). To quantitatively assess this depolymerization capability, three catalytic systems were investigated, including 1,8‐diazabicyclo[5.4.0]undec‐7‐ene (DBU), a DBU/1‐dodecanethiol (DDT) mixture, and tri‐*n*‐butylphosphine (TBP) (Table S3). For each experiment, photo‐cured SPE membranes were ground into a fine powder to maximize surface area, then dispersed in dichloromethane (DCM) in a round‐bottom flask. The chosen catalyst was added at room temperature, and the mixture was stirred until complete dissolution was achieved. Notably, most SPE formulations (containing at least 50 mol% PEG‐LP_2_) achieved complete dissolution within 30 min, with no solid residue remaining in the pale‐yellow solution (Figure 3a). Among the tested catalysts, the DBU/DDT combination enabled the most rapid depolymerization, fully dissolving SPEs with 35 mol% or greater PEG‐LP_2_ content in just 10 min. Using only DBU in solution, complete dissolution occurred within 30 min for SPEs containing 50 mol% or more PEG‐LP_2_, while SPE‐35 required up to 12 h. In contrast, TBP was less effective: SPEs with 35 mol% or less PEG‐LP_2_ were not degraded under these conditions. These results highlight the superior depolymerization efficiency of the DBU/DDT mixture and DBU alone compared to TBP, establishing them as the preferred systems for rapid and complete depolymerization of cross‐linked SPEs. To thoroughly identify and quantify the products formed during depolymerization, particularly the regeneration of the 1,2‐dithiolane ring, we employed ^1^H NMR spectroscopy during the analysis of the final depolymerization mixtures. Across all catalyst treatments, broad signals appeared at 3.1–3.3 ppm and 2.3–2.4 ppm, corresponding to the 1,2‐dithiolane ring (Figure 3d and Figures S25–S27). The amount of recyclable cross‐linker in the degraded solution was quantified using the integral at 3.1–3.3 ppm, corresponding to the α‐methylene protons adjacent to sulfur in the 1,2‐dithiolane ring. As the α‐hydrogens adjacent to the ester group (4.1–4.4 ppm) of the cross‐linker remain unchanged during degradation, their signal was used as an internal reference to normalize the 1,2‐dithiolane ring peak. Equation (1) calculation details are shown below:

(1) RegNMR%=2×int3.1−3.3int4.1−4.4×100

**SCHEME 1:**
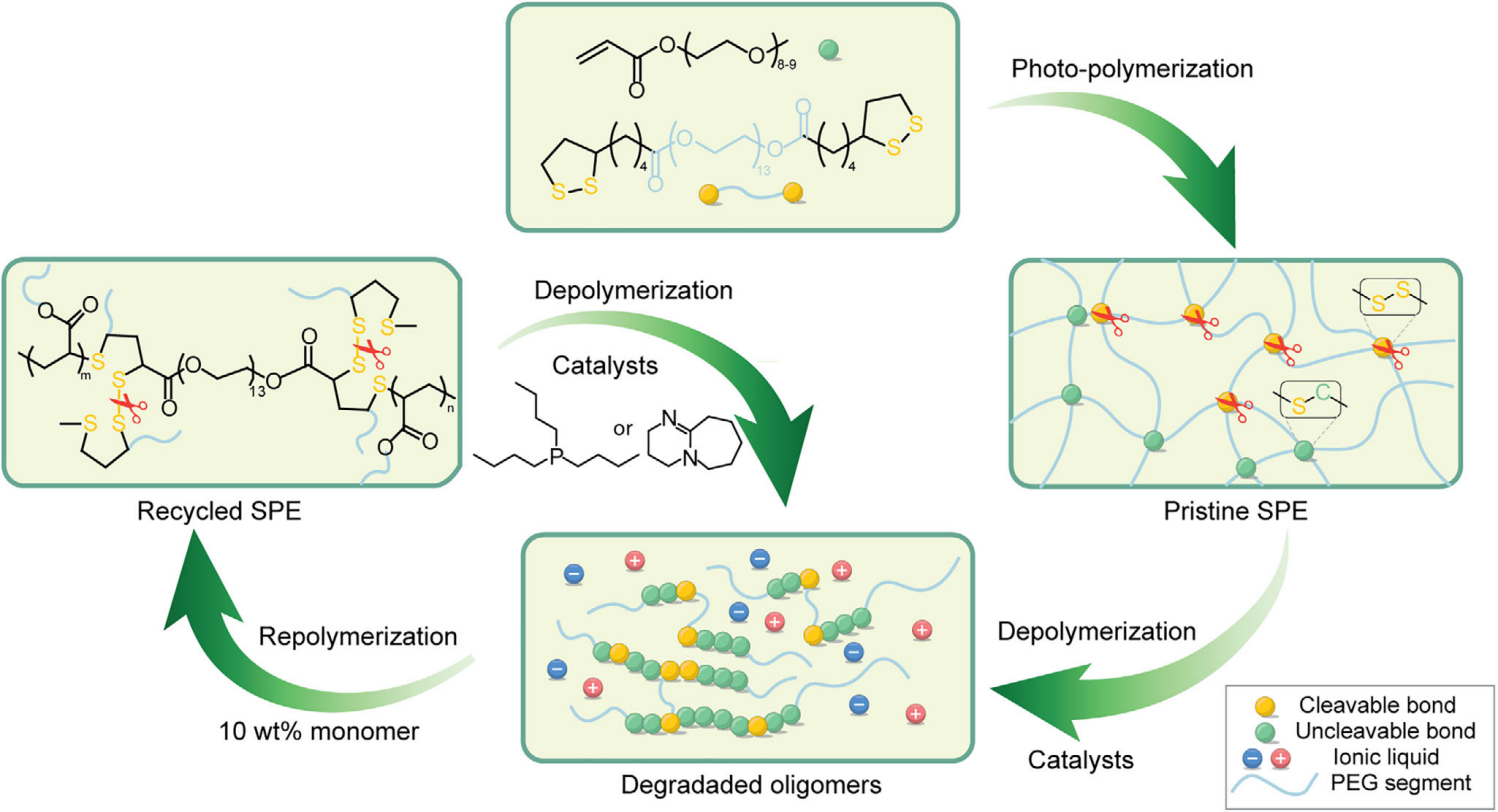
Closed‐loop schematic illustration of recycling: depolymerization and repolymerization of recyclable SPE.

**FIGURE 3:**
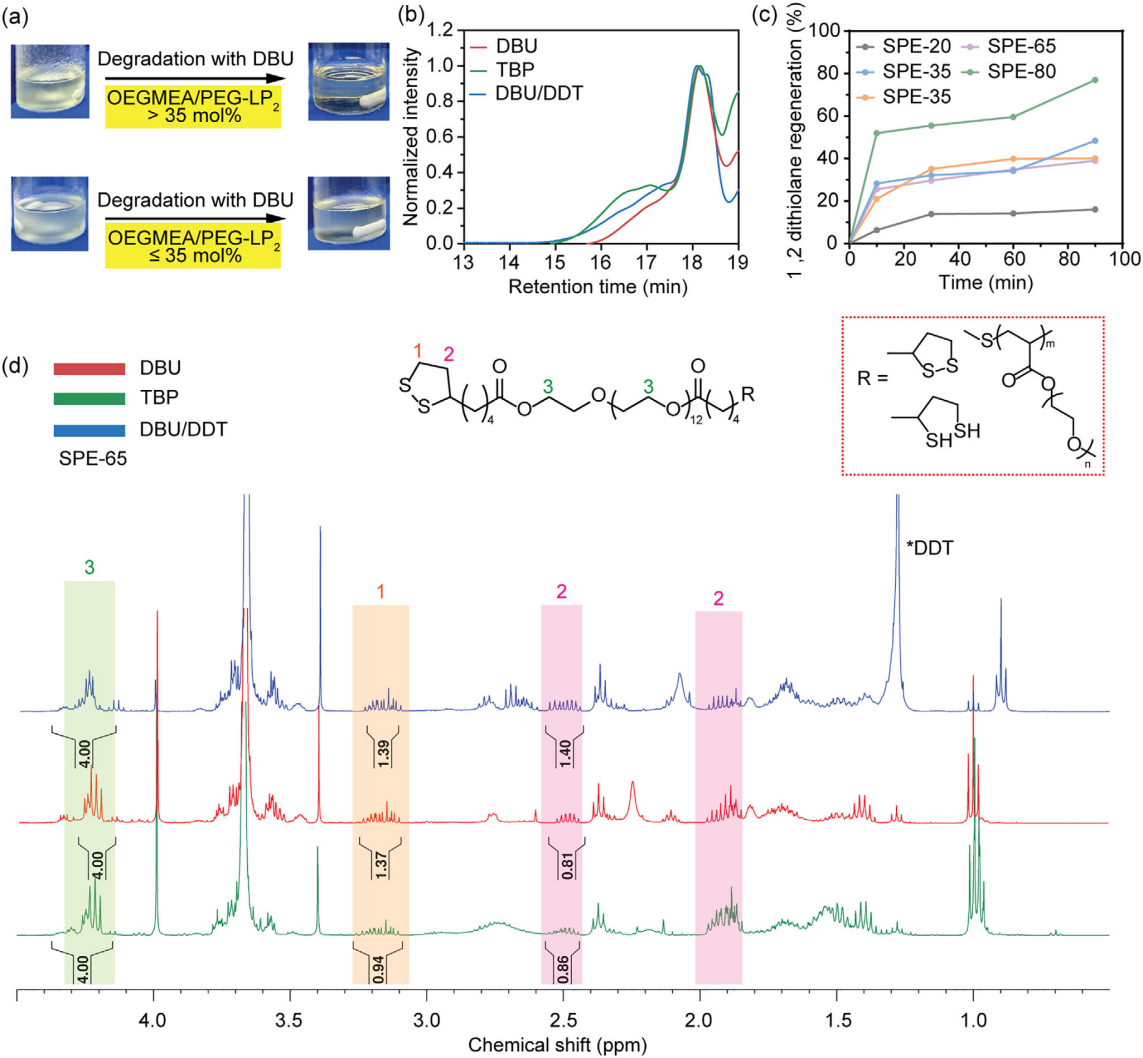
(a) Digital images of membrane degradation using 0.05 M DBU solution. (b) SEC curves of depolymerization final product of SPE‐65 using DBU/DDT mixture, DBU, and TBP, catalysts, respectively. (c) Comparison of 1,2‐dithiolane ring regeneration percentage over time for all formulations using UV–vis light spectroscopy, calculated from Equation (2). (d) ^1^H NMR spectra of the depolymerization final purified product of SPE‐65 using DBU and DDT mixture, DBU and TBP as depolymerization agents, respectively.

Among all catalysts tested, DBU treatment afforded the highest 1,2‐dithiolane ring recovery, with peak integration indicating a regeneration yield of approximately 70%, significantly exceeding those obtained with a DBU/DDT mixture or TBP (Table 1 and Figures S22–S24). We further analyzed the depolymerization products from each catalyst system using size exclusion chromatography (SEC). As illustrated in Figure 3b and Figures S28–S30, the SEC profile of the depolymerization residue closely matched that of PEG‐LP_2_, confirming the ^1^H NMR findings and demonstrating successful recovery of the 1,2‐dithiolane functional group. UV–visible (UV‐vis) spectroscopy was further employed to monitor the depolymerization of DBU‐treated membranes. The 1,2‐dithiolane ring, a diagnostic marker for successful depolymerization and ring regeneration, displays a characteristic absorbance at 335 nm. Absorbance at this wavelength was recorded at 10, 30, 60, and 90‐min intervals to quantify the extent of ring reformation over time. The amount of regenerated cross‐linker in the solution at a given time was calculated from the intensity of the absorption peak at 335 nm, corresponding to the disulfide in the 1,2‐dithiolane ring. Equation (2) calculation details are shown below:

(2) RegUV−Vis%=A335ε×k/m×xV×MwPEG−Lp2×100

where *A*_335_ refers to the intensity of the absorbance peak at 335 nm, *ε* is the molar extinction coefficient, *k* is the dilution ratio, *V* is the volume of the total degradation solution, *m* is the mass of the ground membrane powder, and *x* is the mass fraction of the lipoate cross‐linker PEG‐LP_2_ in the SPE precursor.

**TABLE 1:** Percentage of 1,2‐dithiolane ring regeneration of all SPE formulations with different catalyst(s) measured by ^1^H NMR and UV–vis spectroscopy, respectively.

Entry	Samples	Catalyst (s)	Regeneration rate ^NMR^ (%)^a^	Regeneration rate ^UV–vis^ (%)^b^
1	SPE‐20	DBU solution	—	16%
2	SPE‐35		32%	34%
3	SPE‐50		53%	40%
4	SPE‐65		69%	40%
5	SPE‐80		78%	77%
6	SPE‐20	DBU/DDT mixture	—	—
7	SPE‐35		17%	—
8	SPE‐50		46%	—
9	SPE‐65		57%	—
10	SPE‐80		70%	—
11	SPE‐20	TBP solution	—	—
12	SPE‐35		—	—
13	SPE‐50		47%	—
14	SPE‐65		47%	—
15	SPE‐80		70%	—

^a^ Regeneration rate was calculated by ^1^H NMR spectroscopy using Equation (1).

^b^ Regeneration rate was calculated by UV–vis light spectroscopy using Equation (2).

Membranes with higher disulfide content exhibited a more pronounced and rapid increase in absorbance at 335 nm, indicating more efficient ring regeneration (Figure 3c). For SPE‐80 in particular, the absorbance at 335 nm increased steadily throughout the reaction, consistent with progressive and efficient 1,2‐dithiolane ring reformation as depolymerization proceeded (Figure S31). This enhanced 1,2‐dithiolane regeneration is attributed to the greater abundance of LP‐LP‐LP triads within the copolymer structure, which increases the availability of disulfide bonds for efficient depolymerization and subsequent ring closure.

### 2.3 Repolymerization

Having established optimal depolymerization conditions, residue recovery and repolymerization were investigated for SPE‐65, given its superior electrochemical performance. Consistent with the depolymerization studies, DBU treatment was selected as it provided the highest extent of 1,2‐dithiolane ring regeneration, as confirmed by ^1^H NMR analysis. SEC analysis of the depolymerization product confirmed that it comprises thiol‐terminated POEGMEA oligomers and 1,2‐dithiolane compounds. To achieve successful repolymerization, supplementation with additional OEGMEA monomer and photoinitiator was therefore required. To isolate the recycled SPE precursor, the DBU‐treated SPE‐65 residue was washed with 1 M aqueous HCl to remove the basic catalyst, as residual DBU can prematurely trigger re‐crosslinking [40]. Complete removal of the catalyst was confirmed by ^1^H NMR spectroscopy, which showed the total disappearance of characteristic DBU signals at 1.5 ppm (Figure S32). Following solvent removal, 10 wt% OEGMEA and 2 wt% TPO photoinitiator were added to the recycled product, affording a homogeneous recycled SPE precursor (Table S4). The resulting SPE precursor was directly photo‐cured under the same irradiation conditions as for the pristine membrane (*λ*_max_ = 405 nm, *I*_0_ = 2.06 mW cm^−2^; Scheme 1). A transparent and flexible recycled membrane was successfully obtained after irradiation for 7 min (Figure 4f,g). This recycled membrane (SPE‐65) could be subjected to repeat depolymerization and repolymerization following the same protocol to yield a second recycled membrane.

**FIGURE 4:**
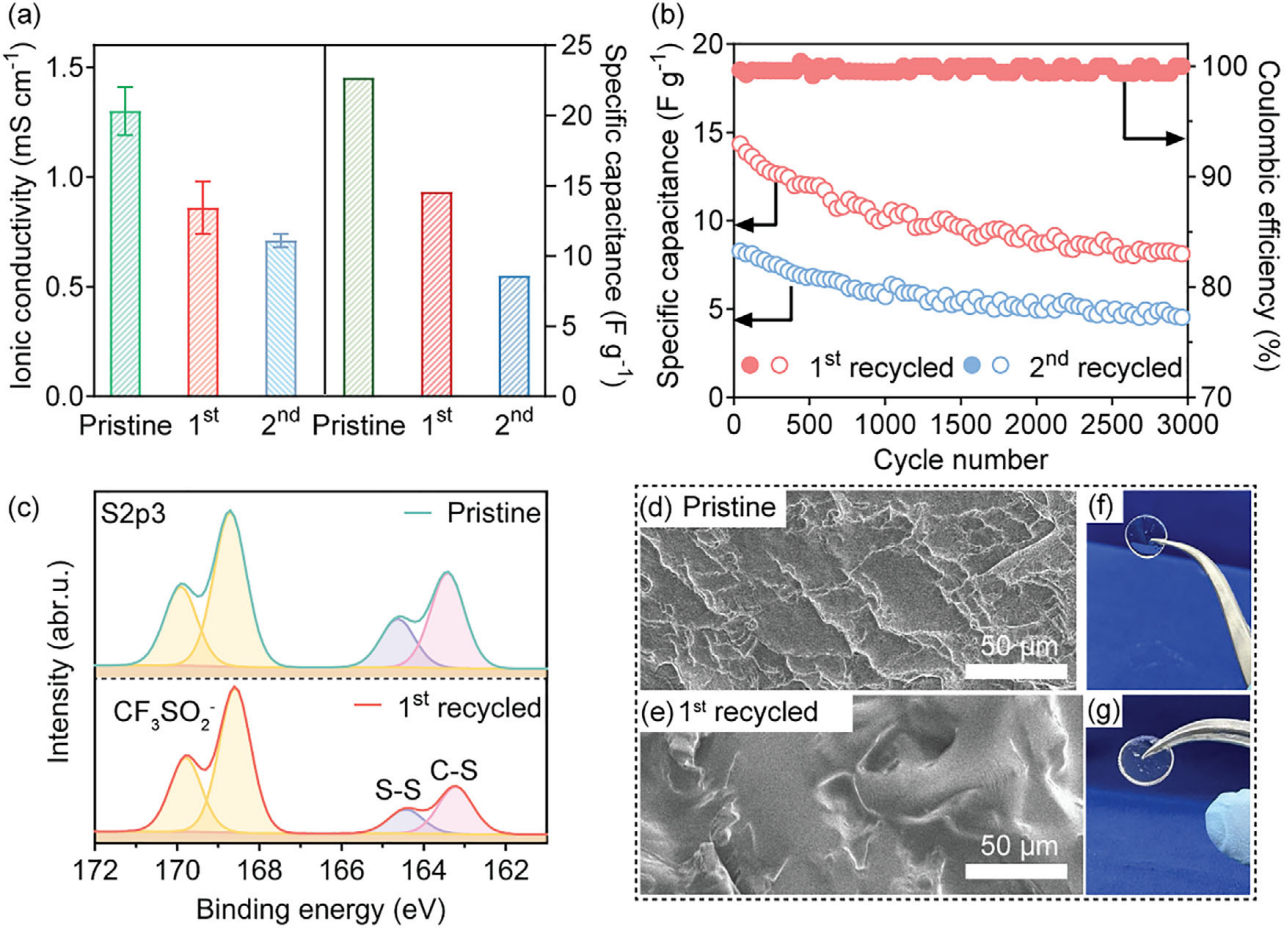
(a) Comparison of ionic conductivity and specific capacitance for pristine and sequentially recycled SPE‐65 membranes. (b) Specific capacitance and Coulombic efficiency of symmetric AC||first recycled SPE‐65||AC supercapacitor and AC||second recycled SPE‐65||AC supercapacitor, respectively. (c) Surface S2p3 XPS spectra for pristine and first recycled SPE‐65, respectively. (d, e) SEM cross‐sectional images of pristine and first recycled SPE‐65, respectively. (f, g) Digital photographs of pristine and first recycled SPE‐65, respectively. *Note*: EIS analysis performed in triplicates; error bars represent the standard deviation obtained from these repeated measurements.

The depolymerization/repolymerization process was quantitatively assessed to demonstrate the recyclability and robustness of our SPE system. For each recycling cycle, ionic liquid retention was determined from the TGA curves by integrating the mass‐loss region corresponding to BMITFSI decomposition, while the overall mass recovery was calculated based on the recycled material after depolymerization (Figure S10). The detailed calculation procedure and values are provided in Table S5. Notably, more than 85% of the ionic liquid and depolymerized oligomer species were recycled after the depolymerization and purification process, confirming the high recycling efficiency of the proposed SPE system. Furthermore, ^19^F NMR spectra of pure BMITFSI and the residues obtained after the first and second depolymerization cycles confirmed the successful recovery and retention of the ionic liquid throughout repeated recycling (Figure S33).

In addition to TGA analysis, DMA measurement was applied to evaluate the mechanical properties of the first recycled membrane. The result demonstrated that the recycled membrane maintained mechanical integrity and thermomechanical stability comparable to that of the pristine membrane, confirming that the recycling process did not compromise their mechanical integrity or thermal stability (Figures S34–S35).

After confirming the structural and thermal stability of the membranes, the electrochemical performance of recycled SPEs was further evaluated. EIS measurements revealed that the first recycled SPE‐65 retained approximately 70% of the ionic conductivity of the pristine membrane, while the second recycled SPE‐65 retained 83% of the conductivity of the first recycled membrane (Figure 4a). It is worth mentioning that the ionic conductivity increased to 93% retention when the first recycled membrane was regenerated with 30 wt% additional OEGMEA monomer (Figure S36). This result indicated that the ion‐transport performance can be effectively restored by compensating for compositional changes during recycling (Figure S37). However, to better reflect a closed‐loop recycling scenario with minimal material replenishment, the recycled residue was combined with only a 10 wt% additional OEGMEA monomer to fabricate the first recycled SPE, which was then assembled into symmetric AC supercapacitors for GCD analysis using the same configuration as for the pristine membrane. GCD evaluations of the AC||first recycled SPE‐65||AC configuration yielded a Coulombic efficiency exceeding 95% over 3000 cycles, demonstrating the robust electrochemical integrity of the dynamic disulfide matrix. The first‐generation recycled membrane preserved 64% of the pristine specific capacitance (22 F g^−1^). Upon undergoing a second recycling iteration (Scheme 1), the resulting membrane retained roughly 60% of the capacitance displayed by the first recycled generation. This performance recovery across successive generations (Figure 4b) demonstrates the potential of the SPE platform for reuse. Compared with the reported cyclic carbonate‐based recyclable system, our approach employed a more straightforward lipoate‐based dynamic cross‐linking strategy, where dynamic disulfide exchange directly occurs within the polymer network [55]. This design may trade some of the electrochemical sophistication and closed‐loop monomer recovery of cyclic carbonate systems for improved synthetic simplicity, processing flexibility, and broader formulation compatibility, providing a practical alternative pathway toward scalable recyclable SPEs.

The reproducibility of SPE fabrication and recycling was further confirmed through repeated experiments, as detailed in the Supporting Information (Section S3.5), with the corresponding quantitative data presented as mean values with standard deviations where applicable.

In addition, x‐ray photoelectron spectroscopy (XPS) was performed on both the pristine and first recycled membranes to confirm their chemical compositions (Figures S38–S40). XPS spectra shown in Figure 4c provided no evidence of side reactions involving the ionic liquid. Notably, the intensity and position of the signals at 167–171 eV, corresponding to S2p binding energy of the sulfonyl group bis(trifluoromethylsulfonyl)imide (CF_3_SO_2_^−^), remains unchanged. This suggests that the ionic liquid was stable toward the strong base DBU during the recycling process. In contrast, the intensity of dynamic disulfide and C─S bonds slightly decreased following the depolymerization–repolymerization cycle, which may contribute to the reduction in overall electrochemical performance. ATR‐FTIR spectroscopy was similarly performed on both membranes (Figure S41). Close overlap of key absorption bands, including the 1,2‐dithiolane ring asymmetric stretch and the imidazolium C–H stretching mode of the ionic liquid, confirmed that the chemical structures of the pristine and first recycled membranes are similar. Scanning electron microscopy (SEM) coupled with energy‐dispersive x‐ray (EDX) spectroscopy were then employed to examine the cross‐sectional morphology and elemental composition of both membranes (Figures S42, S43). The pristine SPE exhibited a dense, continuous cross‐section with uniform thickness and no visible macroscopic defects, consistent with the formation of a homogeneous polymer network. Following repolymerization, the first recycled membrane retained a compact cross‐section morphology, suggesting that the bulk structural integrity of the SPE remains preserved. Although the fracture surface appeared slightly smoother than that of the pristine membrane, no evidence of macroscopic voids or structural defects was observed, suggesting that the recycling process does not significantly alter the bulk membrane structure (Figure 4d,e). SEM‐EDX elemental mapping further confirmed the presence of fluorine and nitrogen in both membranes, demonstrating that the ionic liquid is stable toward treatment with the strong base DBU (Figures S44, S45). EDX elemental mapping revealed homogeneous sulfur distribution in both pristine and first recycled membranes, confirming that polylipoate segments are preserved throughout the depolymerization‐repolymerization cycle.

## 3 Conclusion

In summary, we report a recyclable solid polymer electrolyte platform that integrates a dynamic lipoate cross‐linker with visible‐light photopolymerization, successfully combining high ionic conductivity with closed‐loop chemical recyclability. Among all formulations investigated, the SPE containing 65 mol% PEG‐LP_2_ delivered the highest overall electrochemical performance, consistent with its superior ionic conductivity. When assembled into symmetric supercapacitors, AC||SPE‐65||AC electrochemical cell achieved high specific capacitance, retaining ∼70% of its initial capacitance after 6000 galvanostatic cycles, while exhibiting stable charge–discharge behavior across a range of applied current densities. Compared to conventional non‐recyclable electrolyte membranes, this approach uniquely enables SPE fabrication under mild, ambient conditions, and with rapid photo‐polymerization kinetics, significantly lowering the barriers to practical implementation.

Significantly, the disulfide cross‐linked networks could be fully depolymerized using a range of catalysts, with DBU proving particularly effective. Repolymerization of the recycled oligomers, supplemented with a minor addition of new OEGMEA monomer, yielded the first recycled membranes that retained ∼70% of their initial ionic conductivity and maintained high Coulombic efficiency in a reassembled symmetric AC supercapacitor. To demonstrate the robustness of the closed‐loop process, the regenerated membrane could undergo subsequent depolymerization and repolymerization cycles under identical conditions, successfully yielding a second‐generation recycled membrane. SEM analysis provided structural insight into the first recycled membrane. While the first recycled SPE preserved a dense and continuous cross‐sectional morphology with no evidence of porous structures, it exhibited a smoother and less textured fracture surface relative to the pristine membrane, consistent with the observed reductions in ionic conductivity and specific capacitance. Collectively, these results establish a scalable and efficient route to recyclable polymer electrolyte membranes for supercapacitor applications, providing a design framework for sustainable energy‐storage devices that require balanced electrochemical stability and end‐of‐life recyclability. Beyond recyclability, the use of a bio‐derived lipoate cross‐linker sourced from naturally occurring α‐lipoic acid, a renewable, biosynthetically produced metabolite, adds a further dimension of sustainability to this platform, complementing the closed‐loop recycling strategy and contributing to broader efforts to displace petroleum‐derived polymer components in next‐generation electrochemical devices. While the current study focuses exclusively on supercapacitor applications, future work will be directed toward evaluating the interfacial compatibility and cycling stability of this sustainable SPE platform in lithium‐metal and lithium‐ion battery architectures.

## Author Contributions

**Qian Huang**: investigation, validation, data curation, formal analysis, writing – original draft. **Jeet Sharma**: investigation, validation, data curation, formal analysis, writing – original draft. **Yingna Ding**: investigation, validation, formal analysis, data curation, writing – review and editing. **Kenny Lee**: writing – review and editing, formal analysis, data curation. **Craig J. Hawker**: conceptualization, supervision, writing – review and editing. **Dipan Kundu**: investigation, formal analysis, data curation, writing – review and editing, supervision, resources. **Cyrille Boyer**: conceptualization, project administration, resources, supervision, writing – review and editing, writing – original draft, funding acquisition.

## Conflicts of Interest

The authors declare no conflicts of interest.

## Supporting information



The Supporting Information is available free of charge. Experimental section and additional characterizations, including NMR spectra, SEC traces, UV–vis spectra, FTIR, XPS, SEM spectroscopy, and cyclic voltammetry (Figures S1–S45, Tables S1–S6) are provided in Supporting Information.**Supporting File**: anie74156‐sup‐0001‐SuppMat.pdf.

## Data Availability

The data that support the findings of this study are available in the Supporting Information of this article.

## References

[1] G. Harper , R. Sommerville , E. Kendrick , et al., “Recycling Lithium‐Ion Batteries From Electric Vehicles,” Nature 575 (2019): 75–86, 10.1038/s41586-019-1682-5. 31695206

[2] F. Yang , X. Chen , G. Qu , et al., “Electrode Separation via Water Electrolysis for Sustainable Battery Recycling,” Nature Sustainability 8 (2025): 520–529, 10.1038/s41893-025-01539-3.

[3] S. Yao , X. Wang , Y. Wu , et al., “Interfacial Molecular Engineering of Polymer‐in‐Ceramic Composite Solid Electrolyte for High Performance Solid‐State Lithium Batteries,” Journal of Energy Storage 173 (2026): 123070, 10.1016/j.est.2026.123070.

[4] B. Y. Mekonnen , B. B. Yimam , M. M. Abdelaal , K. Z. Walle , and M. Kotobuki , “Effect of 3‐Aminopropyl Trimethoxysilane Coating on Properties of Lithium Calcium Zirconium Phosphate‐Based Composite Polymer Electrolyte for Lithium Metal Batteries,” Journal of Power Sources 689 (2026): 240690, 10.1016/j.jpowsour.2026.240690.

[5] Y. H. Jo , S. Li , C. Zuo , et al., “Self‐Healing Solid Polymer Electrolyte Facilitated by a Dynamic Cross‐Linked Polymer Matrix for Lithium‐Ion Batteries,” Macromolecules 53 (2020): 1024–1032, 10.1021/acs.macromol.9b02305.

[6] S. Siccardi , J. Amici , S. Colombi , et al., “UV‐Cured Self‐Healing Gel Polymer Electrolyte Toward Safer Room Temperature Lithium Metal Batteries,” Electrochimica Acta 433 (2022): 141265, 10.1016/j.electacta.2022.141265.

[7] F. Elizalde , S. Trano , J. Ayestarán , et al., “3D‐Printable, and Self‐Healable Polymer Electrolyte for Lithium Batteries,” Advanced Functional Materials 35 (2025): 2419034, 10.1002/adfm.202419034.

[8] Y. Wei , Y. Zhang , H. Wang , et al., “Self‐Healing Supramolecular Cathode Binder Additive With Dynamic Bonds for Durable High‐Voltage Solid‐State Lithium Batteries,” eScience 6 (2026): 100555, 10.1016/j.esci.2026.100555.

[9] K. Ahuja , S. P. Singh , P. Joshi , M. S. Lodhi , R. K. Singh , and S. Rana , “Self‐Healable Bio‐Based Solid Polymer Electrolytes Incorporating Ionic Liquids for Safer Lithium–Ion Batteries,” Materials Advances 7 (2026): 6254–6265, 10.1039/D6MA00073H.

[10] J. Huang , S. Wang , J. Chen , C. Chen , and E. Lizundia , “Environmental Sustainability of Natural Biopolymer‐Based Electrolytes for Lithium Ion Battery Applications,” Advanced Materials 37 (2025): 2416733, 10.1002/adma.202416733. PMC12138847 39757715

[11] Y.‐H. Lu and Y.‐L. Liu , “Self‐Healing and Externally Recoverable Solid Polymer Electrolytes for Lithium Metal Batteries With Enhanced Cycling Stability,” ACS Applied Energy Materials 9 (2026): 7803–7812.

[12] H. Lv , X. Chu , Y. Zhang , Q. Liu , F. Wu , and D. Mu , “Self‐Healing Solid‐State Polymer Electrolytes for High‐Safety and Long‐Cycle Lithium‐Ion Batteries,” Materials Today 78 (2024): 181–208, 10.1016/j.mattod.2024.06.018.

[13] B. B. Jing and C. M. Evans , “Catalyst‐Free Dynamic Networks for Recyclable, Self‐Healing Solid Polymer Electrolytes,” Journal of the American Chemical Society 141 (2019): 18932–18937, 10.1021/jacs.9b09811. 31743006

[14] L. Zhu , H. Yang , K. Liu , et al., “Molecularly Engineered Cellulose: The Next‐Generation Sustainable Polymer Electrolyte Material,” Energy & Environmental Science 19 (2026): 1407–1457, 10.1039/D5EE05398F.

[15] M. Ahuis , S. Doose , D. Vogt , P. Michalowski , S. Zellmer , and A. Kwade , “Recycling of Solid‐State Batteries,” Nature Energy 9 (2024): 373–385, 10.1038/s41560-024-01463-4.

[16] Y. Zhao , Y. Kang , J. Wozny , et al., “Recycling of Sodium‐Ion Batteries,” Nature Reviews Materials 8 (2023): 623–634, 10.1038/s41578-023-00574-w.

[17] B. K. Biswal , B. Zhang , P. T. M. Tran , J. Zhang , and R. Balasubramanian , “Recycling of Spent Lithium‐Ion Batteries for a Sustainable Future: Recent Advancements,” Chemical Society Reviews 53 (2024): 5552–5592, 10.1039/D3CS00898C. 38644694

[18] P. Simon and Y. Gogotsi , “Materials for Electrochemical Capacitors,” Nature Materials 7 (2008): 845–854, 10.1038/nmat2297. 18956000

[19] K. Ge , H. Shao , Z. Lin , P.‐L. Taberna , and P. Simon , “Advanced Characterization of Confined Electrochemical Interfaces in Electrochemical Capacitors,” Nature Nanotechnology 20 (2025): 196–208, 10.1038/s41565-024-01821-z. 39639178

[20] Q. Zhao , S. Stalin , C.‐Z. Zhao , and L. A. Archer , “Designing Solid‐State Electrolytes for Safe, Energy‐Dense Batteries,” Nature Reviews Materials 5 (2020): 229–252, 10.1038/s41578-019-0165-5.

[21] S. Han , P. Wen , H. Wang , et al., “Sequencing Polymers to Enable Solid‐State Lithium Batteries,” Nature Materials 22 (2023): 1515–1522, 10.1038/s41563-023-01693-z. 37845320

[22] X. Gao , W. Yuan , Y. Yang , et al., “High‐Performance and Highly Safe Solvate Ionic Liquid‐Based Gel Polymer Electrolyte by Rapid UV‐Curing for Lithium‐Ion Batteries,” ACS Applied Materials & Interfaces 14 (2022): 43397–43406, 10.1021/acsami.2c13325. 36102960

[23] M. Hamrahjoo , S. Hadad , E. Dehghani , M. Salami‐Kalajahi , and H. Roghani‐Mamaqani , “Poly(poly(ethylene glycol) Methyl Ether Methacrylate‐co‐acrylonitrile) Gel Polymer Electrolytes for High Performance Lithium Ion Batteries: Comparing Controlled and Conventional Radical Polymerization,” European Polymer Journal 173 (2022): 111276, 10.1016/j.eurpolymj.2022.111276.

[24] S. A. Langevin , B. Tan , A. W. Freeman , et al., “UV‐Cured Gel Polymer Electrolytes With Improved Stability for Advanced Aqueous Li‐Ion Batteries,” Chemical Communications 55 (2019): 13085–13088, 10.1039/C9CC06207F. 31608913

[25] H. Gao , V. Riesgo‐Gonzalez , J. R. Runge , et al., “Recyclable Li‐Metal Battery Electrolytes via In Situ Cyclic Carbonate Polymerization,” Advanced Science 12 (2025): e04206, 10.1002/advs.202504206. PMC12407366 40490987

[26] P. Chen , S. Liu , H. Zhou , et al., “Closed‐Loop Recyclable Solid‐State Polymer Electrolytes Enabled by Reversible Lithium Salt Catalysis,” Journal of the American Chemical Society 147 (2025): 7624–7633, 10.1021/jacs.4c17035. 39993282

[27] Y. Cho , C. D. Fincher , G. Lamour , et al., “Reversible Self‐Assembly of Small Molecules for Recyclable Solid‐State Battery Electrolytes,” Nature Chemistry 18 (2025): 1023–1032, 10.1038/s41557-025-01917-6. 40877574

[28] Q. Zhang , Y. Deng , C.‐Y. Shi , B. L. Feringa , H. Tian , and D.‐H. Qu , “Dual Closed‐Loop Chemical Recycling of Synthetic Polymers by Intrinsically Reconfigurable Poly(disulfides),” Matter 4 (2021): 1352–1364, 10.1016/j.matt.2021.01.014.

[29] T. O. Machado , C. J. Stubbs , V. Chiaradia , et al., “A Renewably Sourced, Circular Photopolymer Resin for Additive Manufacturing,” Nature 629 (2024): 1069–1074, 10.1038/s41586-024-07399-9. PMC11136657 38750360

[30] S. Pal , J. Shin , K. DeFrates , et al., “Recyclable Surgical, Consumer, and Industrial Adhesives of Poly(α‐lipoic acid),” Science 385 (2024): 877–883, 10.1126/science.ado6292. 39172835

[31] J. Guo , S. Zhang , Y. Tao , et al., “Synthesis of Cationic Cyclic Oligo(disulfide)s via Cyclo‐Depolymerization: A Redox‐Responsive and Potent Antibacterial Reagent,” Journal of the American Chemical Society 147 (2025): 6772–6785, 10.1021/jacs.4c16627. 39945514

[32] T. Pesenti and J. Nicolas , “100th Anniversary of Macromolecular Science Viewpoint: Degradable Polymers From Radical Ring‐Opening Polymerization: Latest Advances, New Directions, and Ongoing Challenges,” ACS Macro Letters 9 (2020): 1812–1835, 10.1021/acsmacrolett.0c00676. 35653672

[33] S. Yang , S. Du , J. Zhu , and S. Ma , “Closed‐Loop Recyclable Polymers: From Monomer and Polymer Design to the Polymerization–Depolymerization Cycle,” Chemical Society Reviews 53 (2024): 9609–9651, 10.1039/D4CS00663A. 39177226

[34] K. Lee , S. Han , P. T. Morris , C. M. Bates , C. J. Hawker , and C. Boyer , “Controlled Synthesis of Lipoate Homopolymers via Reversible Addition–Fragmentation Chain Transfer Polymerization,” Journal of the American Chemical Society 147 (2025): 38796–38806, 10.1021/jacs.5c14377. 41051070

[35] Z. Guan , “Sustainable Polymers That Stick Inside and Out,” Science 385 (2024): 829–830, 10.1126/science.adr5857. 39172855

[36] H. Yun , K. Wang , J. Zhang , G. Peng , and H. Zhao , “Construction of Peptide‐Lipoic Acid Cationic Polymers With Redox Responsiveness and Low Toxicity for Gene Delivery,” ACS Omega 9 (2024): 3499–3506. 10.1021/acsomega.3c07194 PMC10809251 38284089

[37] K. R. Albanese , J. Read de Alaniz , C. J. Hawker , and C. M. Bates , “From Health Supplement to Versatile Monomer: Radical Ring‐Opening Polymerization and Depolymerization of *α*‐Lipoic Acid,” Polymer 304 (2024): 127167, 10.1016/j.polymer.2024.127167.

[38] Y. Liu , Y. Jia , Q. Wu , and J. S. Moore , “Architecture‐Controlled Ring‐Opening Polymerization for Dynamic Covalent Poly(disulfide)s,” Journal of the American Chemical Society 141 (2019): 17075–17080, 10.1021/jacs.9b08957. 31603692

[39] K. R. Albanese , P. T. Morris , J. Read de Alaniz , C. M. Bates , and C. J. Hawker , “Controlled‐Radical Polymerization of α‐Lipoic Acid: A General Route to Degradable Vinyl Copolymers,” Journal of the American Chemical Society 145 (2023): 22728–22734, 10.1021/jacs.3c08248. PMC10591472 37813389

[40] S. Han , V. A. Bobrin , M. Michelas , C. J. Hawker , and C. Boyer , “Sustainable and Recyclable Acrylate Resins for Liquid‐Crystal Display 3D Printing Based on Lipoic Acid,” ACS Macro Letters 13 (2024): 1495–1502, 10.1021/acsmacrolett.4c00600. 39446026

[41] K. R. Albanese , P. T. Morris , B. Roehrich , J. Read de Alaniz , C. J. Hawker , and C. M. Bates , “Selective Electrochemical Degradation of Bottlebrush Elastomers,” Journal of Polymer Science 62 (2024): 4326–4331, 10.1002/pol.20240393.

[42] Y. Okayama , P. Morris , K. Albanese , et al., “Enhanced Degradation of Vinyl Copolymers Based on Lipoic Acid,” Journal of Polymer Science 63 (2025): 1345–1351, 10.1002/pol.20241085.

[43] M. Czuczola , M. S. Hossain , D. P. Shannon , et al., “Telechelic Dithiol Copolymers as Tunable Building Blocks for Synthesizing Multiblock Materials,” Journal of Polymer Science 63 (2025): 759–765, 10.1002/pol.20240876.

[44] X. Dai , K. Lee , Y. Xiu , et al., “3D‐Printable Nanoporous Thermosets via Disulfide‐Based Polymerization‐Induced Microphase Separation,” Angewandte Chemie International Edition (2026): e8227484, 10.1002/anie.8227484. PMC13452562 42287685

[45] C. Choi , Y. Okayama , P. T. Morris , et al., “Digital Light Processing of Dynamic Bottlebrush Materials,” Advanced Functional Materials 32 (2022): 2200883, 10.1002/adfm.202200883.

[46] H. Jiang and Z. An , “Visible Light Photoiniferter Polymerization for the Synthesis of Degradable Lipoate Copolymers,” ACS Macro Letters 14 (2025): 1641–1645, 10.1021/acsmacrolett.5c00583. 41092080

[47] Y. Deng , L. Liu , H.‐X. Luo , et al., “Supramolecular Chemical Recycling of Dynamic Polymers,” Nature Nanotechnology 20 (2025): 1805–1812, 10.1038/s41565-025-02041-9. 41193862

[48] Z. Rong , Y. Sun , M. Yang , F. Cheng , W. Zhang , and J. Chen , “How the PEG Terminals Affect the Electrochemical Properties of Polymer Electrolytes in Lithium Metal Batteries,” Energy Storage Materials 63 (2023): 103066, 10.1016/j.ensm.2023.103066.

[49] K. Lee , Y. Shang , V. A. Bobrin , et al., “3D Printing Nanostructured Solid Polymer Electrolytes With High Modulus and Conductivity,” Advanced Materials 34 (2022): 2204816, 10.1002/adma.202204816. 36007199

[50] M. Wang , X. Xiao , S. Siddika , et al., “Glassy Gels Toughened by Solvent,” Nature 631 (2024): 313–318, 10.1038/s41586-024-07564-0. 38898283

[51] D. Melodia , A. Bhadra , K. Lee , et al., “3D Printed Solid Polymer Electrolytes With Bicontinuous Nanoscopic Domains for Ionic Liquid Conduction and Energy Storage,” Small 19 (2023): 2206639, 10.1002/smll.202206639. 36737816

[52] D. Kitto and J. Kamcev , “The Need for Ion‐Exchange Membranes With High Charge Densities,” Journal of Membrane Science 677 (2023): 121608, 10.1016/j.memsci.2023.121608.

[53] I. O. Levkovsky , L. Trachsel , H. Murata , and K. Matyjaszewski , “Oxygen‐Tolerant, Red Light‐Driven Controlled Synthesis of Easily Degradable and High Molecular Weight α‐Lipoic Acid‐Vinyl Copolymers,” Angewandte Chemie International Edition 64 (2025): e202516164, 10.1002/anie.202516164. PMC12559452 40916180

[54] S. Han , K. Lee , P. T. Morris , et al., “Light‐Induced Copolymerization of Ethyl Lipoate and Vinyl Monomers: Increased Efficiency and Degradability,” Macromolecules 58 (2025): 13288–13299, 10.1021/acs.macromol.5c02734.

[55] M. Mondal , P. E. Jankoski , L. D. Lee , et al., “Reversible Disulfide Bond Cross‐Links as Tunable Levers of Phase Separation in Designer Biomolecular Condensates,” Journal of the American Chemical Society 146 (2024): 25299–25311, 10.1021/jacs.4c09557. PMC11403603 39196681

