# Supplementary material for: Closed‐Loop Recyclable Solid Polymer Electrolytes via Dynamic Lipoate Cross‐Linking
Source: Angew Chem Int Ed Engl. 2026 Aug 17;65(40):e3540036. doi: 10.1002/anie.3540036 (PMC13618284; doi:10.1002/anie.3540036)
Supplement: Supplementary file 1 [file ANIE-65-e3540036-s001.pdf]

Supporting Information

Closed-Loop Recyclable Solid Polymer Electrolytes via Dynamic Lipoate Cross-linking

Qian Huang,[a] Jeet Sharma,[a] Yingna Ding,[b] Kenny Lee,[a,c] Craig J. Hawker,[c] Dipan Kundu,[b,d] and Cyrille Boyer*[a]

[a] Cluster for Advanced Macromolecular Design, School of Chemical Engineering, University of New South Wales, Sydney, New South Wales, Australia

[b] School of Chemical Engineering, UNSW Sydney, Sydney, New South Wales, Australia

[c] Materials Department and Department of Chemistry and Biochemistry, University of California, Santa Barbara, California, USA.

[d] School of Mechanical and Manufacturing Engineering, UNSW Sydney, Sydney, New South Wales, Australia

*Corresponding Email: cboyer@unsw.edu.au

Contents

1. Materials **1**

2. Characterization **1**

2.1 Nuclear magnetic resonance (NMR) spectroscopy *1*

2.2 Attenuated total reflectance-Fourier transform infrared (ATR-FTIR) spectroscopy *1*

2.3 Size exclusion chromatography (SEC) *2*

2.4 Dynamic mechanical analysis (DMA) testing *2*

2.5 Thermogravimetric analysis (TGA) *2*

2.6 UV-visible (UV-Vis) light spectroscopy *2*

2.7 Scanning electron microscopy (SEM) *3*

2.8 X-ray photoelectron spectroscopy (XPS) *3*

2.9 Electrochemical impedance spectroscopy (EIS) *3*

2.10 Assembly of symmetric supercapacitors for electrochemical performance testing *4*

2.11 Electrochemical performance evaluation *4*

3. Methods **5**

3.1 Synthesis of lipoate cross-linker (PEG-LP$_2$) *5*

3.2 Preparation of SPE precursor *5*

3.3 Photo-curing procedure *5*

3.4 Degradation and recovery *6*

3.5 Experimental reproducibility *6*

4. Additional data **7**

1. Materials

Poly(ethylene glycol) methyl ether acrylate (OEGMEA, M_n = 480), poly(ethylene glycol) (PEG, M_n = 600), 1,8-diazabicyclo[5.4.0]undec-7-ene (DBU, ≥ 99%), 1-dodecanethiol (DDT, ≥ 98%), dichloromethane (DCM, anhydrous, = 99.8%), poly(vinylidene fluoride) (PVDF, average M_w = 534,000), Super P (carbon average particle size ≈ 40 nm) 4-(dimethylamino)pyridine (DMAP, ≥ 99%), 1-butyl-3-methylimidazolium bis(trifluoromethylsulfonyl)imide (BMITFSI, ≥ 98%), tri-*n*-butylphosphine (TBP, ≥ 99%) were purchased from Sigma Aldrich. *N*'-(ethylcarbonimidoyl)-*N,N*-dimethylpropane-1,3-diamine hydrochloride (EDC-HCl, ≥ 99%), α-lipoic acid (LA, ≥ 97%), and diphenyl(2,4,6-trimethylbenzoyl)phosphine oxide (TPO, ≥ 97%) were purchased from AmBeed. Ethanol (EtOH, ChemSupply AU, ≥ 99.5%), sodium bicarbonate ($NaHCO_3$, ChemSupply, ≥ 99%), sodium chloride (NaCl, ChemSupply, ≥ 99%), sodium sulfate (Na_2SO_4, ChemSupply, ≥ 99%), magnesium sulfate ($MgSO_4$, ChemSupply, ≥ 99%), hydrochloric acid (HCl, ChemSupply, 32%), and N-methyl-2-pyrrolidone (NMP, ChemSupply ≥ 99.5%) were purchased from ChemSupply Australia. Activated carbon (AC, ~2500 m^2 g^{-1}) was purchased from Jiangsu Xfnano Materials Tech Co., Ltd. All reagents and solvents were used as received unless stated otherwise.

2. Characterization

2.1 Nuclear magnetic resonance (NMR) spectroscopy

^{1}H, ^{13}C and ^{19}F NMR spectra were recorded using a Bruker Avance III 400 MHz spectrometer. Proton NMR signals are reported using $CDCl_3$ as deuterated solvent (δ = 7.26 ppm). All the NMR spectra were analyzed using TopSpin 4.4.1 software. The amount of recyclable cross-linker in the degraded solution was quantified using the integral at 3.1–3.3 ppm of ^{1}H NMR, corresponding to the dynamic disulfide bond in 1,2-dithiolane ring. As the α-hydrogens adjacent to the ester group (4.1–4.4 ppm) of the cross-linker remain unchanged during degradation, their signal was used as an internal reference to normalize the 1,2-dithiolane ring peak.

2.2 Attenuated total reflectance-Fourier transform infrared (ATR-FTIR) spectroscopy

ATR-FTIR spectroscopy was used to monitor the polymerization kinetics of the vinyl bond and to compare the pristine and recycled membranes. A Bruker Alpha FTIR spectrometer equipped with a room-temperature DTGS detector was used to determine the double bond conversion in the SPE precursor by calculating the integral of the vinylic stretching overtone at 1600-1650 cm^{-1} normalized to the C=O stretching overtone at 1660–1780 cm^{-1} as an internal standard. 60 µL SPE precursors were pipetted onto the ATR crystal plate after background scanning. An absorption spectrum was obtained by scanning the stretching overtone of the droplet from 400–4000 cm^{-1} for 8 s as t_0. A Thorlabs mounted LED with a collimation adapter (λ_{max} = 405 nm, I_0 = 2.06 mW cm^{-2}) was used to irradiate the droplet SPE precursor at various times before obtaining the absorption spectrum by determining the integral of the vinylic peak at x using **Equation S1** below. To evaluate experimental reproducibility, photopolymerization kinetics were performed at least three times with three independently prepared samples under identical conditions. The reported values are presented as mean values,

and the error bars in the corresponding **Figures S5-6** represent the standard deviation obtained from repeated measurements.

$$\alpha_t\ (\%) = 100 \times (1 - \frac{int_x - Std_x}{int_0 - std_0}) \quad (1)$$

where α_t is the double bond conversion after irradiation for t_x s, int_x and std_x is the integral of the stretching peak of 1600-1650 cm^{-1} and 1660-1780 cm^{-1} at x seconds of irradiation respectively, int_0 and std_0 is the initial integral of the stretching peak of 1600-1650 cm^{-1} and 1660-1780 cm^{-1} peak before irradiation, respectively.

2.3 Size exclusion chromatography (SEC)

Polymers and oligomers were analyzed using tetrahydrofuran (THF) as the eluent. A Shimadzu modular system composed of a SIL-20A auto-injector, oven at 40 °C, a Phenomenex 5.0 µm bead-size guard column (50 × 7.5 mm) followed by two Agilent MIXED C columns (PLgel 5 µm), and a Shimadzu RID-10A refractive-index detector was used to determine the molecular weight and dispersity of polymers. The system was calibrated using Agilent narrow molecular weight distribution poly(methyl methacrylate) (PMMA) standards with molecular weights (M_n) ranging from 545 to 2,210,000 g mol^{-1}.

2.4 Dynamic mechanical analysis (DMA) testing

All DMA experiments were conducted using a rectangular prism sample with dimensions of 30 × 8 × 2 mm (length × width × thickness). Single cantilever testing methodology was applied for DMA testing. The analysis was performed using a TA instruments Q800 dynamic mechanical analyzer. Initially, a digital caliper was used to measure the dynamic cross-linked SPEs before placing the sample into the single cantilever clamp, which was then tightened using a torque wrench with a force of 1.2 in lb. The samples were subjected to a temperature ramp of 2 °C/min up from -70 °C to room temperature (30 °C), with a constant oscillation frequency of 1 Hz and a displacement of 10 µm. All the samples were determined in the same procedure. To evaluate experimental reproducibility, DMA analysis was conducted twice with two independent strip samples under identical conditions. Both data are identical and represented data was shown in **Figures S7-8**.

2.5 Thermogravimetric analysis (TGA)

The thermogravimetric analysis (TGA) of SPE membranes was performed from room temperature to 600 °C at a rate of 15 °C min^{-1} under nitrogen as well as under air using a TA Instrument TGA Q5000 thermogravimetric analyzer.

2.6 UV-visible (UV-Vis) light spectroscopy

A Varian Cary 300 spectrophotometer was used to determine all UV-Vis spectra. The scans range was from 800 to 200 nm at a recording speed of 600 nm/min. Before every sample scanning, a 2 × 10 mm path length quartz cuvette containing pure DCM was used for baseline scanning. The UV-Vis instrument was then zeroed at 800 nm. All samples were run in the same quartz cuvette size. At 10 min, 30 min, 60 min, and 90 min, an aliquot was taken from the degradation solution and diluted to an appropriate concentration for UV-Vis measurement to ensure the absorption intensity remained between 0 and 1. Using the Beer–Lambert law, the amount of regenerated crosslinker in the solution at a given time was calculated from the intensity of the

absorption peak at 335 nm, corresponding to the disulfide in the 1,2-dithiolane ring. This allowed determination of the regeneration rate at specific time intervals.

2.7 Scanning electron microscopy (SEM)

Scanning electron microscopy (SEM) was employed to analyze the elemental composition of the pristine membrane and that of the recycled membrane using a Bruker QUANTAX energy dispersive X-ray spectroscopy (EDX) system. A field-emission FEI Nova NanoSEM 450 instrument with 5 – 18 kV accelerating voltage and a secondary electron detector was used for all SEM experiments. A 3 mm circular membrane with 0.5 mm thickness was mounted onto the holder using carbon tape before SEM experiments were performed.

2.8 X-ray photoelectron spectroscopy (XPS)

X-ray photoelectron spectroscopy (XPS) was used to analyze the elemental composition of the pristine and 1st recycled membrane. Thin film samples (11 mm diameter, 0.45 mm thickness) were photo-polymerized prior to measurement using a Thermo ESCALAB250Xi XPS instrument. All surface elements within a binding energy range of 0–1350 eV were determined quantitatively.

2.9 Electrochemical impedance spectroscopy (EIS)

The ionic conductivity of the recyclable SPE was determined by two-probe AC impedance spectroscopy (VMP3, Biologic) over a frequency range of 1 MHz to 100 mHz with a potentiostatic signal perturbation of 50 mV. Before measurement, one drop of BMITFSI was placed on the titanium rod to ensure good contact with the membrane discs (11 mm diameter, 0.45 mm thickness), which were mounted in a symmetric Swagelok-type cell sandwiched between two titanium rods. EClab Z-fit analysis software was used to fit the Nyquist impedance plots so that the ionic conductivities of SPEs with different polymer architectures could be measured. A model circuit $\frac{CPEb}{Rb} + CPEint$ used to fit the Nyquist profiles, where CPE_b and R_b refer to the bulk SPE resistance and constant phase element (capacitance), respectively, and *CPEint* represents the constant phase element corresponding to the interfacial capacitance. A reasonably good fit was obtained in all cases with a goodness of fit (χ^2/|Z|; where χ^2 is the difference between the experimental and fitted data and |Z| is the modulus of impedance) in the range of 10^{-2} – 10^{-3}. To examine the temperature dependence of conductivity, samples with 65 mol % PEG-LP_2 were conducted only by heating from 30 °C to 60 °C, followed by cooling down to 30 °C. Conductivity was recorded at each 15 °C intervals. Heating and cooling processes were carried out at a constant temperature with insulation duration of 30 min and 60 min, respectively. The ion conductivity was calculated by **Equation S2** below. To validate experimental reproducibility, impedance spectroscopy analysis was performed at least three times with three independently prepared samples under identical conditions. The reported values are presented as mean values, and the error bars in the corresponding **Figures 2a** and **Figure 4a** represent the standard deviation obtained from repeated measurements.

$$\sigma = \frac{l}{AR} \tag{2}$$

where l is the thickness of the sample disc, A refers to the cross-sectional area of the membrane, R represents the resistance fitted by circuit model $\frac{CPE_b}{R_b} + CPEint$.

2.10 Assembly of symmetric supercapacitors AC||SPE||AC for electrochemical performance testing

The electrode material was prepared by adding the activated carbon (AC, 80 %) with Super P carbon (10%) in the mortar, grinding for 30 min until a uniform black powder was obtained. The mixed carbon was transferred to a weighing boat. PVDF (10%) was dissolved in NMP to form a homogeneous solution in the mortar. The mixed carbon was then gradually added to the PVDF/NMP solution in batches with continued grinding over 30 min to form a slurry. The obtained slurry was coated onto the current collector graphite foils by doctor-blading and subsequently dried at 40 °C under vacuum for 24 h, yielding electrodes with 2.5 mg cm^{-2} AC loading. A symmetric Swagelok-type cell was constructed, where the SPE and two activated carbon (AC) electrodes were sandwiched between two titanium rods, to fabricate a supercapacitor device.

2.11 Electrochemical performance evaluation

VMP3 (Biologic) and Landt battery cycler were used to conduct galvanostatic charge-discharge (GCD) tests, floating stability and cyclic voltammetry (CV), respectively. All the SPE membranes were assembled as AC||SPE||AC symmetric supercapacitor for evaluation of GCD analysis at a current density of 100 mA g^{-1} with a voltage window of 0 to 1 V. For AC||SPE-65||AC only, a current density variation cycling experiment was performed (current density range from 50 mA g^{-1} to 250 mA g^{-1} with a 25 mA g^{-1} interval). Each current density was applied for 50 cycles for one loop, and five loops were employed for a total of 2250 cycles. The floating stability of the symmetric AC||SPE-65||AC supercapacitor was measured by galvanostatic charge-discharged at 250 mA g^{-1} within a voltage window of 0 V-1 V, followed by open-circuit voltage monitoring to evaluate voltage retention and device stability. Electrochemical impedance spectroscopy was collected before and after the floating test to examine changes in impedance and verify the electrochemical stability of SPE-65 in the assembled device. CV characterization was conducted using AC supercapacitor/SPE-65 only. The scan rate varied from 1 mV s^{-1} to 100 mV s^{-1} with a voltage window of 0 to 2 V.

3. Methods

3.1 Synthesis of lipoate cross-linker (PEG-LP$_2$)

To synthesize lipoate cross-linker poly(ethylene glycol) bis(lipoate) (PEG-LP$_2$), poly(ethylene glycol) (PEG, M_n = 600, 1 equiv., 7.93 g, 13.22 mmol), α-lipoic acid (LA, 2.2 equiv., 6 g, 29.08 mmol) and DMAP (2.2 equiv., 3.6 g, 29.47 mmol) were dissolved in anhydrous DCM (50 mL) and placed in a 250 mL round-bottom flask in an ice bath at 0 °C. The reaction mixture was stirred for 15 min before the solution of EDC-HCl (2.2 equiv., 5.57 g, 29.06 mmol) in anhydrous DCM (50 mL) added portionwise over 30 min. The ice bath was removed, and the reaction mixture was wrapped in aluminum foil. After stirring for 24 h, the crude product was washed with hydrochloric acid (3 times), saturated sodium bicarbonate solution (twice), and brine (once). The crude product was dried over anhydrous Na_2SO_4. The solvent was removed under reduced pressure, and the final product was dried in a vacuum oven at 40 °C overnight.

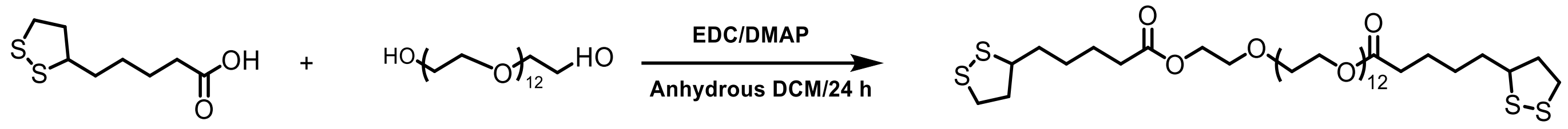

Scheme S1. Synthesis of lipoate cross-linker (PEG-LP$_2$) via EDC/DMAP coupling reaction.

3.2 Preparation of SPE precursor

All the SPE precursors were prepared according to the values reported in **Table S1** at the same procedure**.** The weight ratio of BMITFSI in all SPE precursors remains at 45 wt%. r[monomer]:r[cross-linker] varied from 80/20 mol% to 20/80 mol%. For instance, 5.5 g of SPE precursor of SPE-65 were prepared by dissolving 750 mg monomer OEGMEA in 2.5 g BMITFSI, followed by adding 2.25 g PEG-LP$_2$ and 11 mg TPO. These SPE precursor mixtures were mixed by vortex for 30 s before being placed in a bath sonicator (Hwashin powersonic 410, 400 W) for 10 min. The homogeneous mixture obtained was then directly used for photo-curing under violet light irradiation (λ_{max} = 405 nm, I_0 = 2.06 mW cm^{-2}) for 3 min.

Table S1. Summary of SPE precursor formulations in this study.

SPE precursor formulations	OEGMEA (mol %)	PEG-LP$_2$ (mol %)	TPO (wt %)	BMITFSI (wt %)	Cure time
SPE-20	80	20	2.0	45	3 min
SPE-35	65	35	2.0	45	3 min
SPE-50	50	50	2.0	45	3 min
SPE-65	35	65	2.0	45	3 min
SPE-80	20	80	2.0	45	3 min

weight percentage of monomers plus that of cross-linker is 55 wt % in total.

3.3 Photo-curing procedure

All pristine SPE samples were photo-cured with a violet light LED array (λ_{max} = 405 nm, I_0 = 2.06 mW cm^{-2}) for 3 min. For electrochemical analysis, a pre-cut silicone mold with a cavity of 11 mm in diameter and 0.45 mm in thickness was used to fabricate circular sample membrane. For the membrane reuse experiment, the

recycled SPE precursors were placed in the same silicone mold under a violet light LED array (λ_{max} = 405 nm, I_0 = 2.06 mW cm^{-2}). The resulting recycled membranes were denoted as the 1st recycled membrane and 2nd recycled membrane, respectively

3.4 Degradation and recovery

A 150 × 1.5 × 0.5 mm strip photo-cured object was ground with a mortar and pestle, then transferred to a 50 mL round-bottom flask to prepare a 20 mg/mL DCM solution. Depolymerization catalysts (0.05 M DBU/0.05 M DBU and 0.01 M DDT mixture/0.05 M TBP) were added to the flask to give a 0.05 M solution. The reaction mixture was stirred at room temperature for 30 min until complete dissolution. Thereafter, 1 M hydrochloric acid (HCl) solution was added to quench the DBU, and the mixture was stirred for a further 15 min at room temperature. A separatory funnel was used to remove the HCl solution, and the remaining crude product was dried over magnesium sulfate before concentration using a rotary evaporator. Finally, a yellow oily recycled SPE precursor was obtained. For the preparation of the reused membrane, 10 wt% additional monomer OEGMEA was combined with the recycled residue (90 wt%) in a 20 mL vial. An additional 2 wt% photoinitiator TPO was added to the vial. The mixture was sonicated for 10 min to give a homogeneous solution. The reproducibility of the proposed recyclable SPE system was evaluated through repeated experiments. At least three repeated tests were performed. The relatively small variation in NMR spectra among repeated measurements confirms the good reproducibility of the polymerization behavior of the recycled system.

3.5 Experimental reproducibility

To evaluate the reproducibility of the proposed recyclable SPE system, key experiments were repeated using independently prepared samples or repeated experimental runs under identical conditions. Unless otherwise stated, photopolymerization kinetics, ionic conductivity, degradation, and repolymerization experiments were performed at least three times. The reported values are presented as mean values, and the error bars in the corresponding figures represent the standard deviation obtained from repeated measurements. Specifically, ATR-FTIR kinetic measurements were conducted using three independently prepared samples, and the corresponding conversion profiles are shown with standard deviations in **Figures S5-6**. EIS was also performed on at least three independently prepared membranes, with error bars included in **Figure 2a** and **Figure 4a**. DMA measurements were conducted using two independent strip samples under identical conditions, and the obtained thermomechanical profiles were highly consistent; representative data are shown in **Figures S7-8**. For the degradation and repolymerization experiments, repeated tests gave consistent ^{1}H NMR spectra and successful membrane regeneration, confirming the reproducibility of the depolymerization/repolymerization process.

4. Additional data

Characterization of lipoate cross-linker (PEG-LP$_2$)

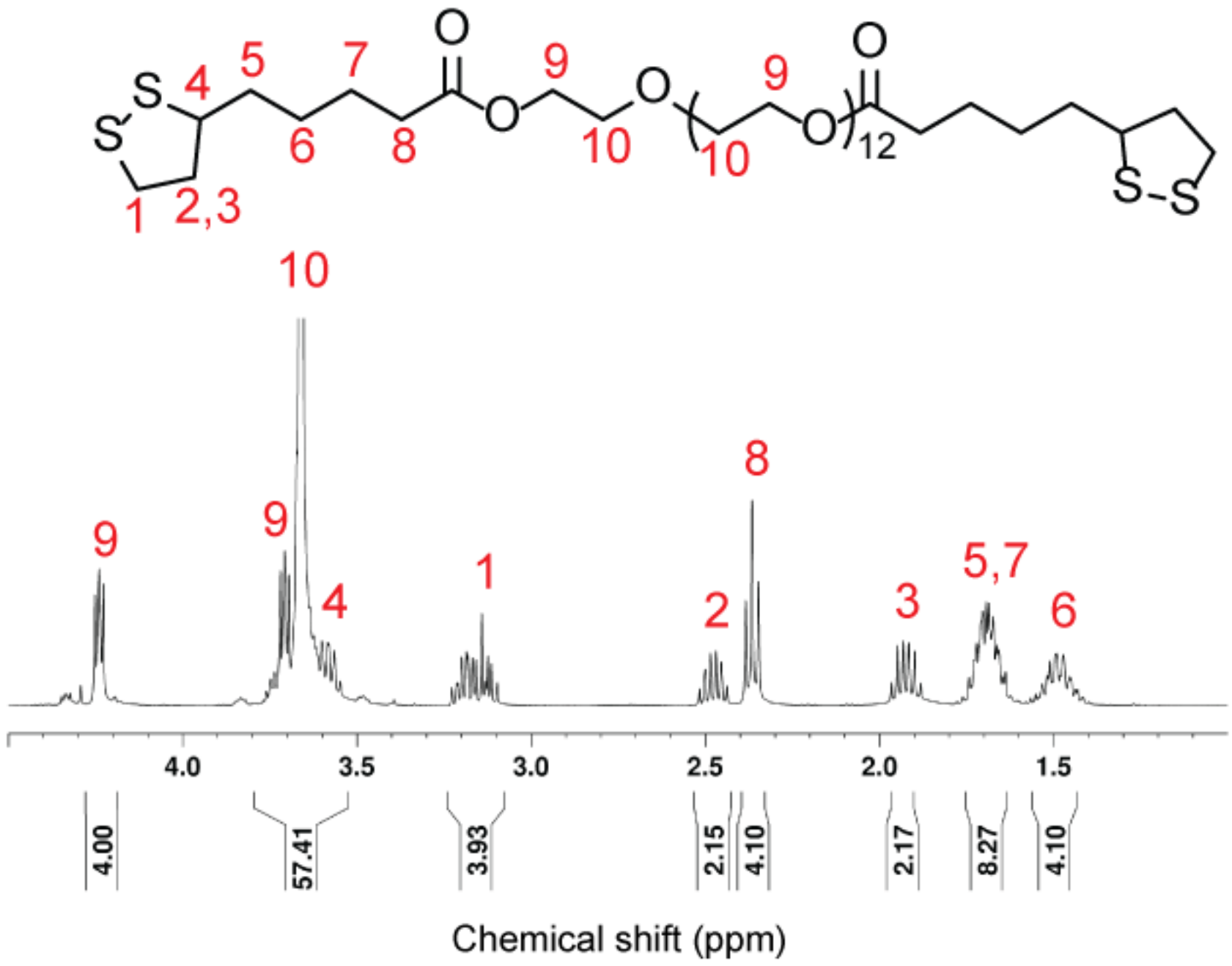

Figure S1. ^{1}H NMR spectrum of PEG-LP$_2$ recorded in $CDCl_3$ (400 MHz, 298 K).

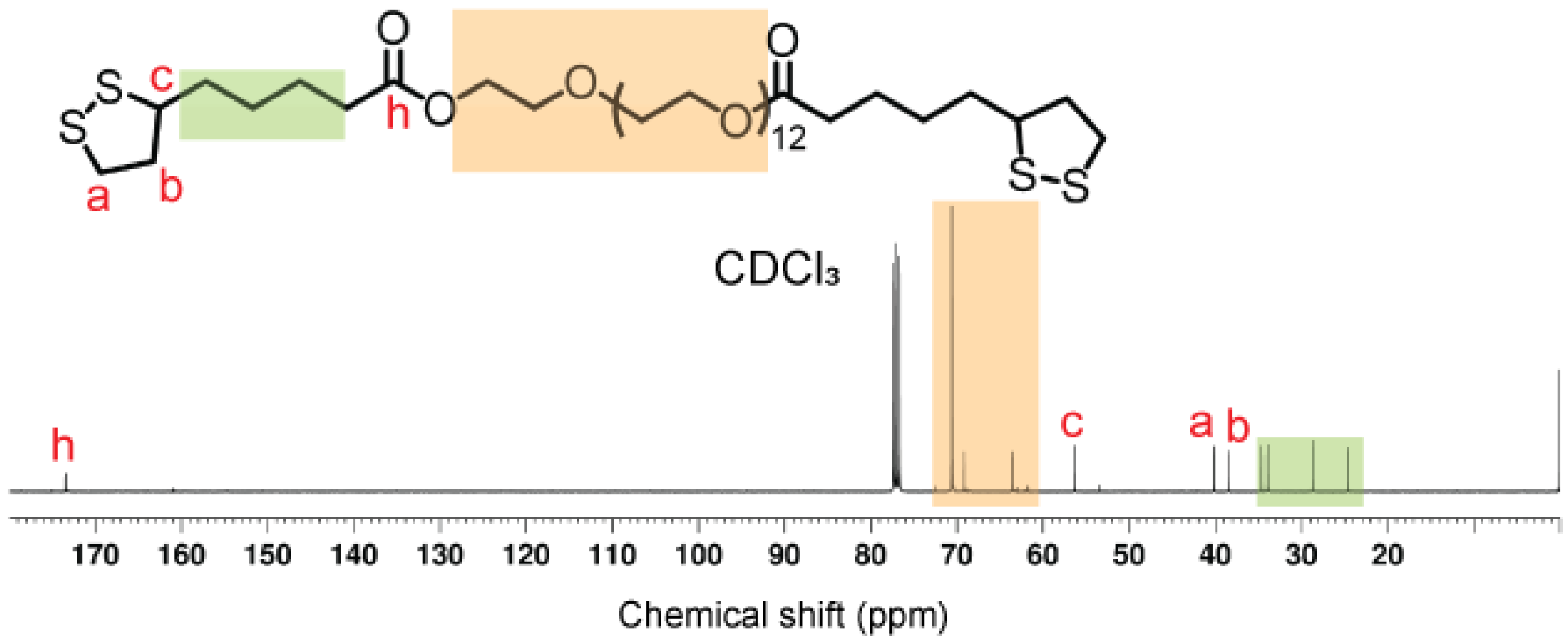

Figure S2. ^{13}C NMR spectrum of PEG-LP$_2$ recorded in $CDCl_3$ (400 MHz, 298 K).

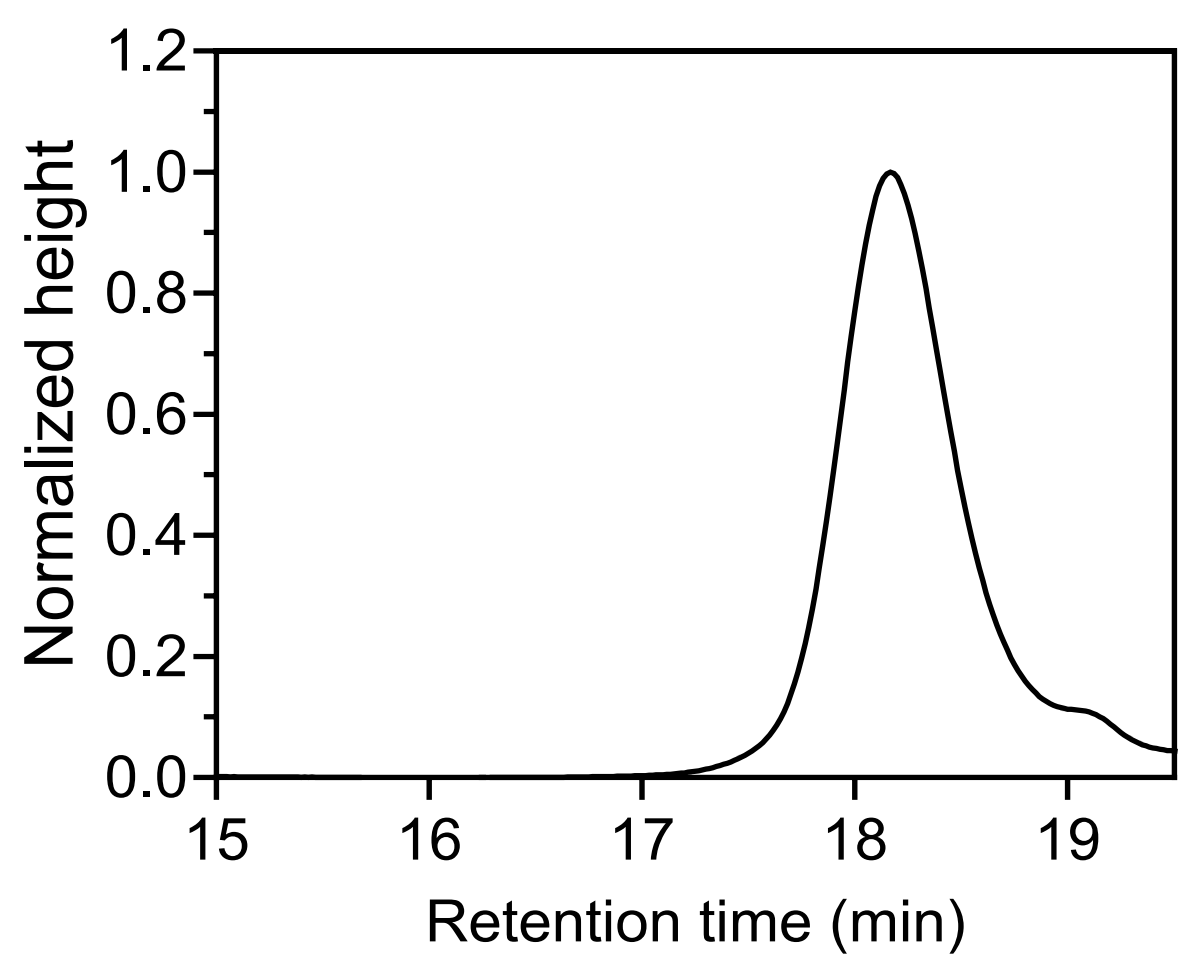

Figure S3. SEC trace of PEG-LP$_2$ using THF as eluent.

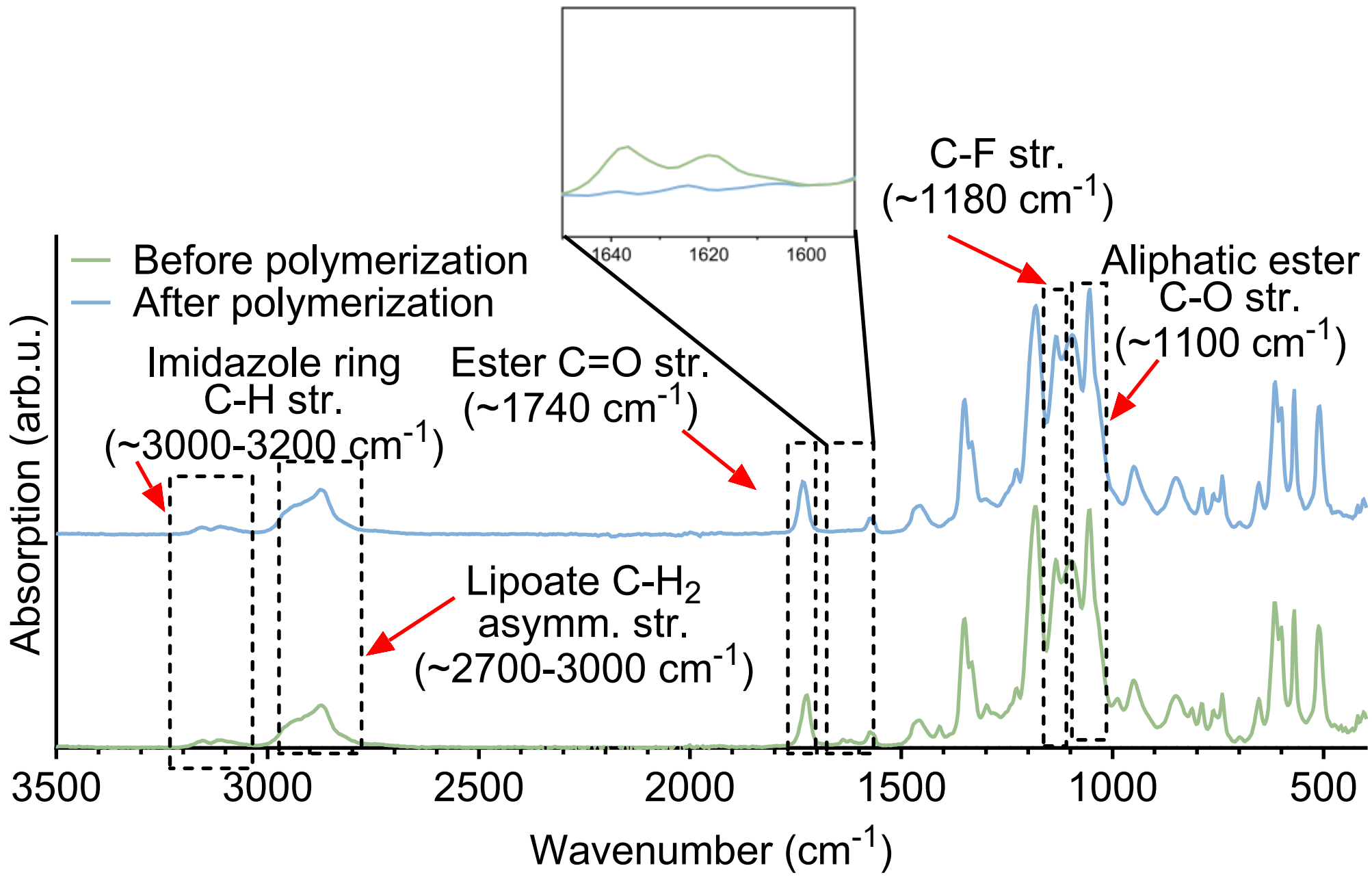

Figure S4. ATR-FTIR spectra of SPE-20 before and after polymerization under 405 nm violet light LED array (I_0 = 2.06 mWcm^{-2}).

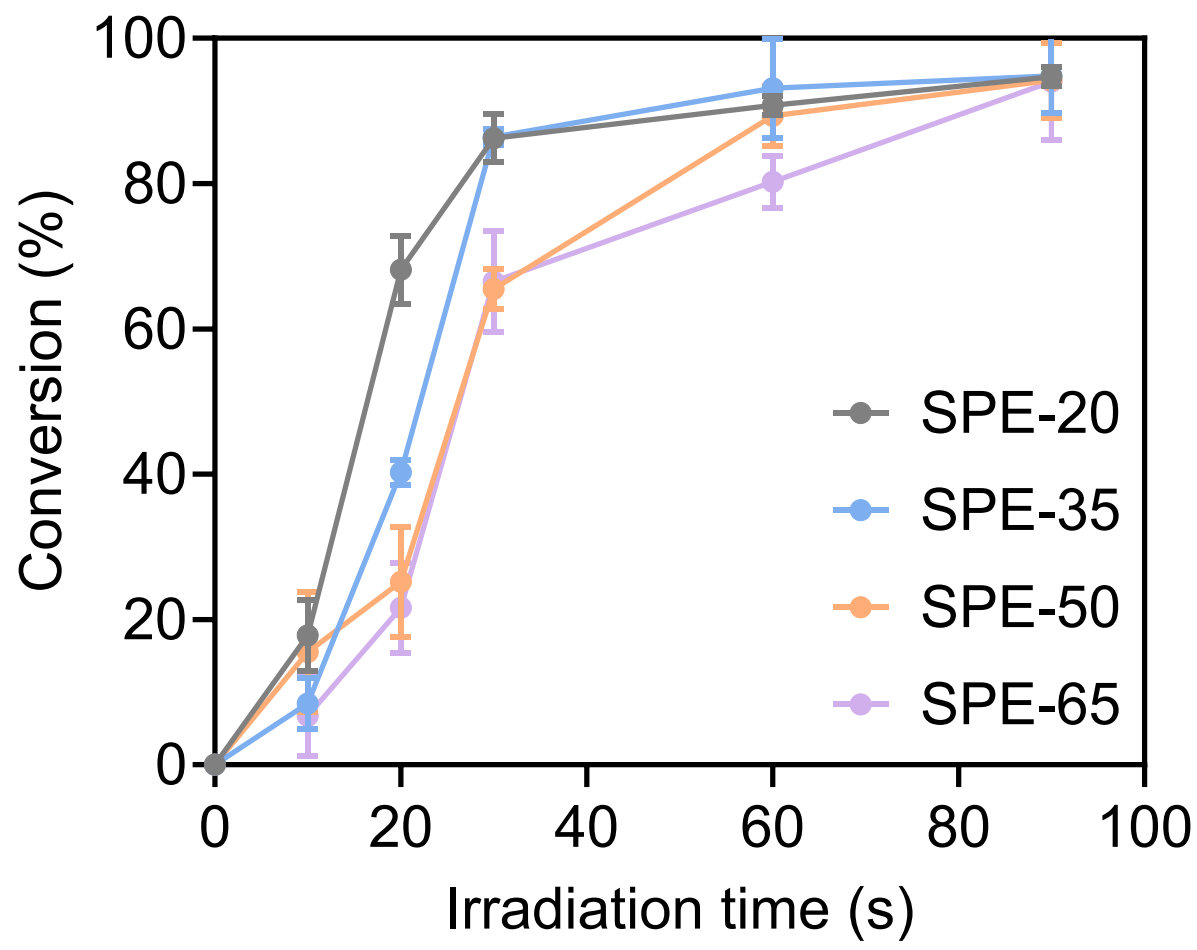

Figure S5. Photopolymerization kinetics monitored by ATR-FTIR spectroscopy based on double-bond conversion. Vinyl conversion profiles of all SPE formulations under irradiation with a 405 nm violet LED array at a light intensity of 2.06 mW cm^{-2}. Note: Results performed in triplicates; error bars represent the standard deviation obtained from these repeated measurements.

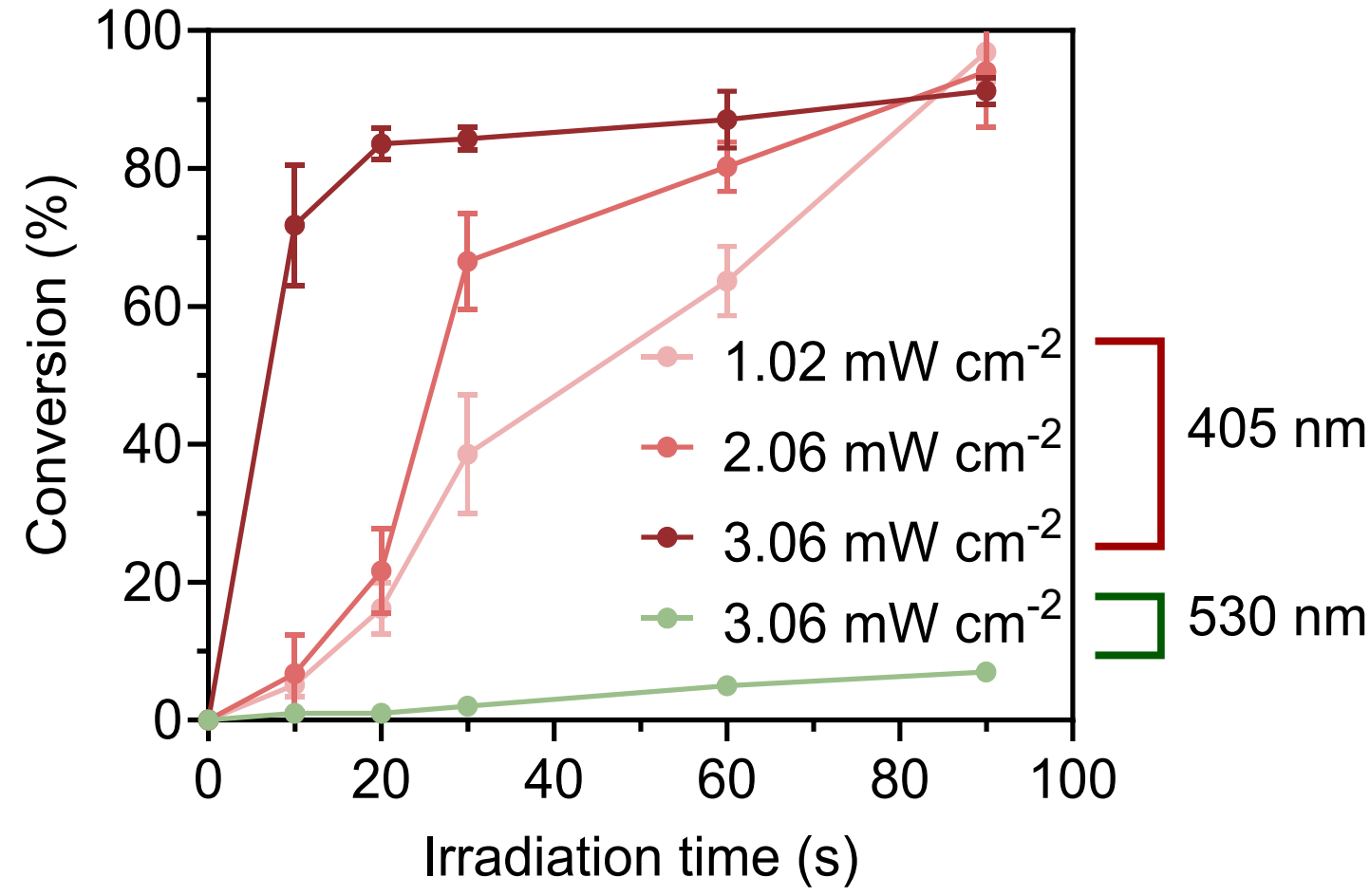

Figure S6. Photopolymerization kinetics monitored by ATR-FTIR spectroscopy based on double-bond conversion. Vinyl conversion kinetics of SPE-65 under different light intensities using 405 nm violet LED, compared with irradiation under 3.06 mW cm^{-2} at 530 nm. Note: Results performed in triplicates; error bars represent the standard deviation obtained from these repeated measurements.

Table S2. Glass transition temperature (T_g) and storage modulus G' @20 °C of SPE membranes.

	SPE-20	SPE-35	SPE-50	SPE-65	SPE-80	SPE-65 1st recycled
T_g (°C)	-46.5	-45.0	-43.3	-43.4	-44.7	-37.0
Storage modulus G' (MPa, @ 20 °C)	1.29	0.84	0.86	2.14	1.21	2.93

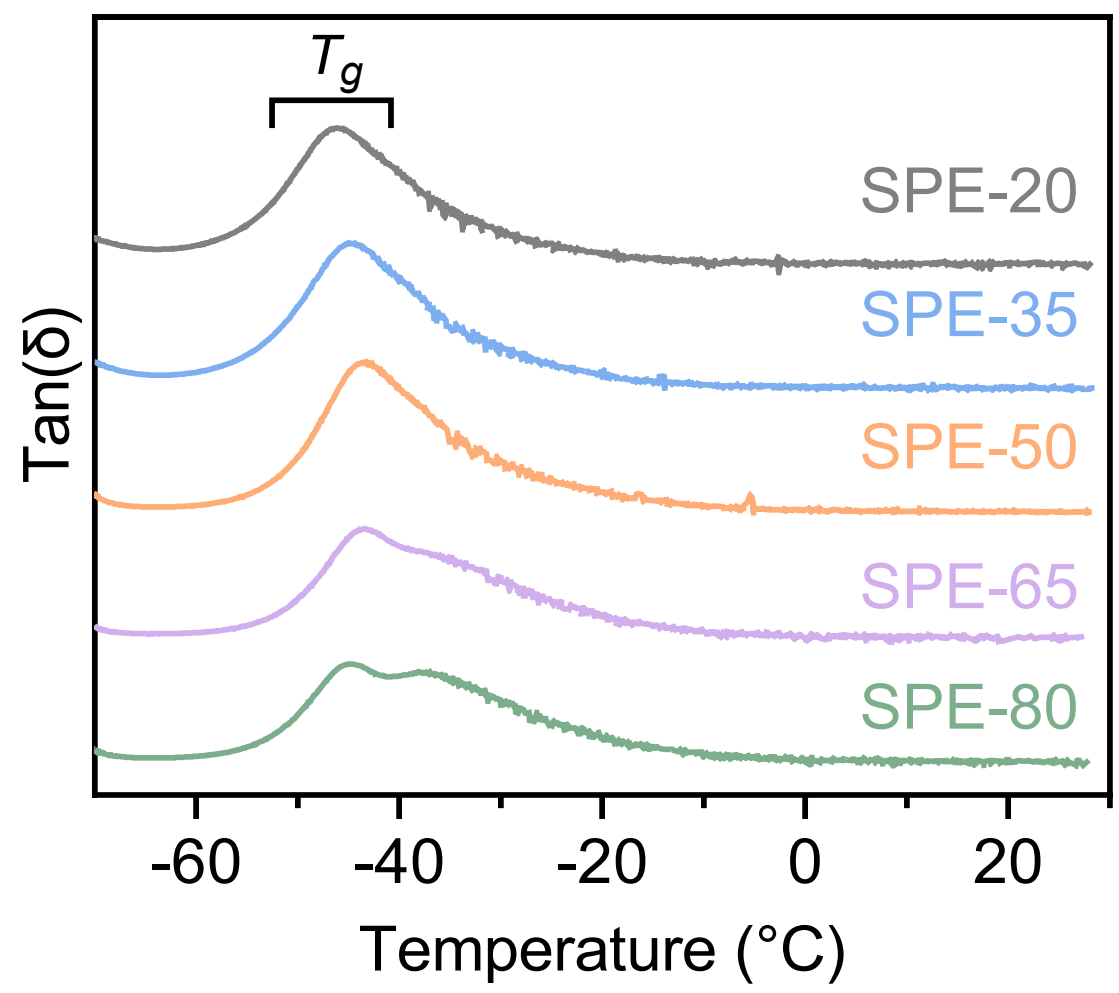

Figure S7. Tan(δ) as a function of temperature for all formulations from -70 to 25 °C.

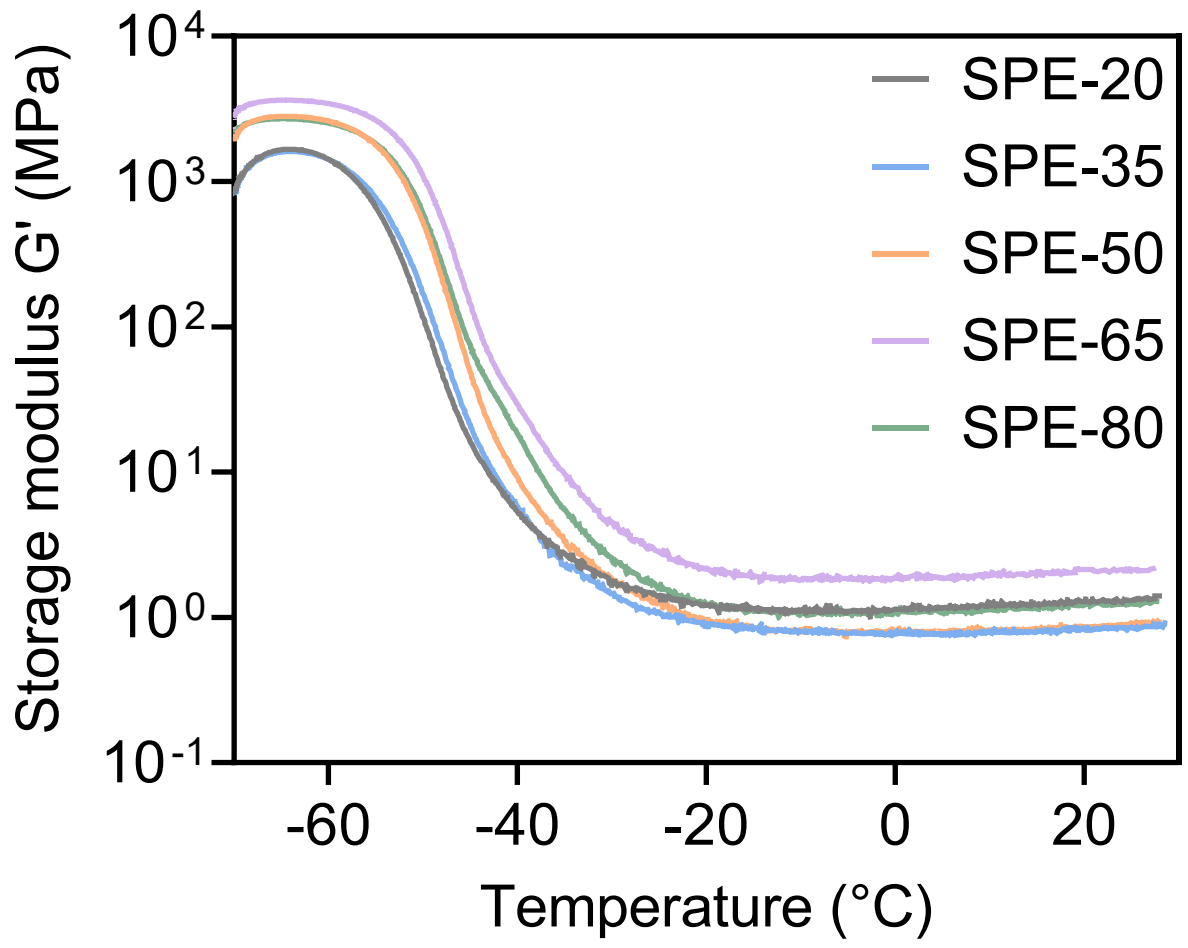

Figure S8. Storage modulus as a function of temperature for all formulations. Note: DMA analysis was conducted at least twice using three independently prepared samples under identical conditions, and representative curves are shown.

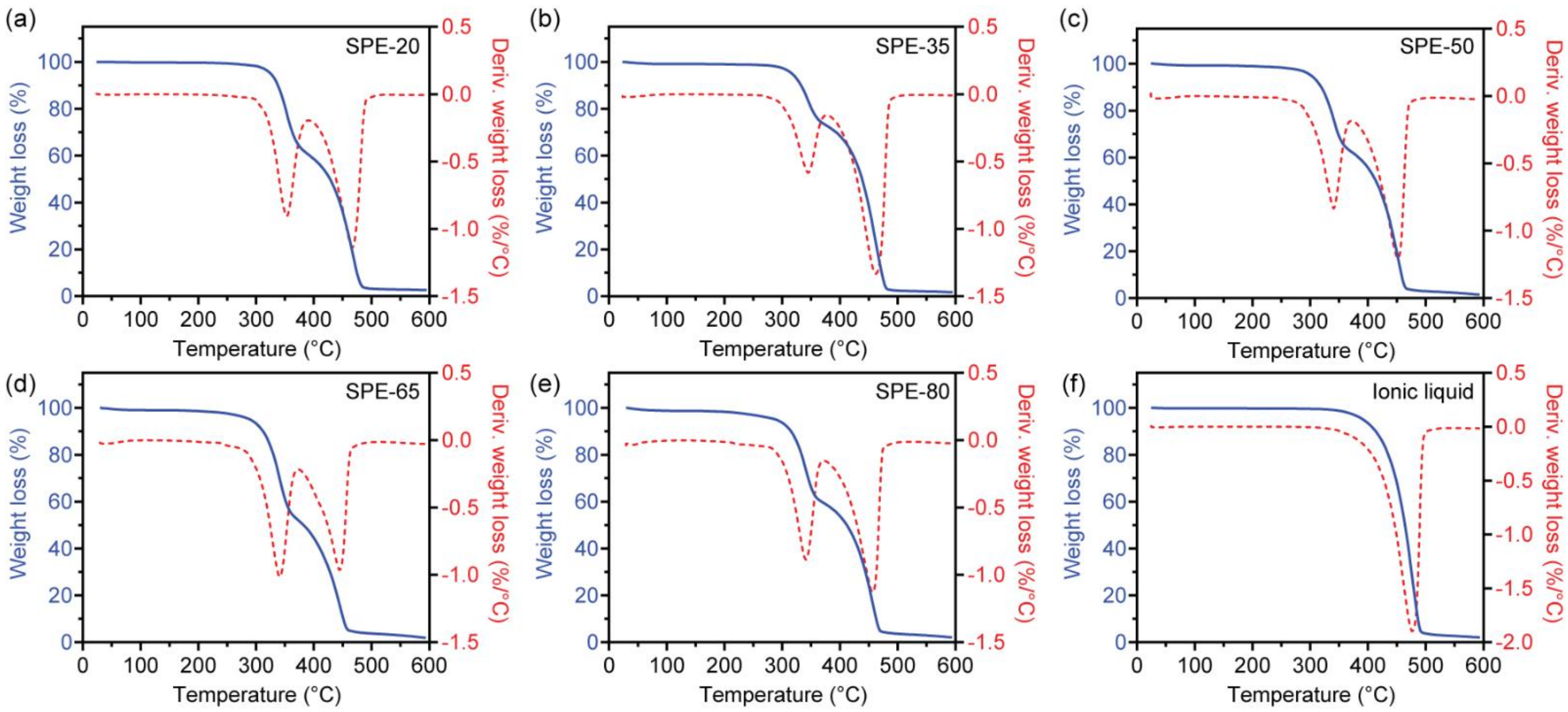

Figure S9. Thermogravimetric analysis (TGA) curves of the recyclable SPE formulations.

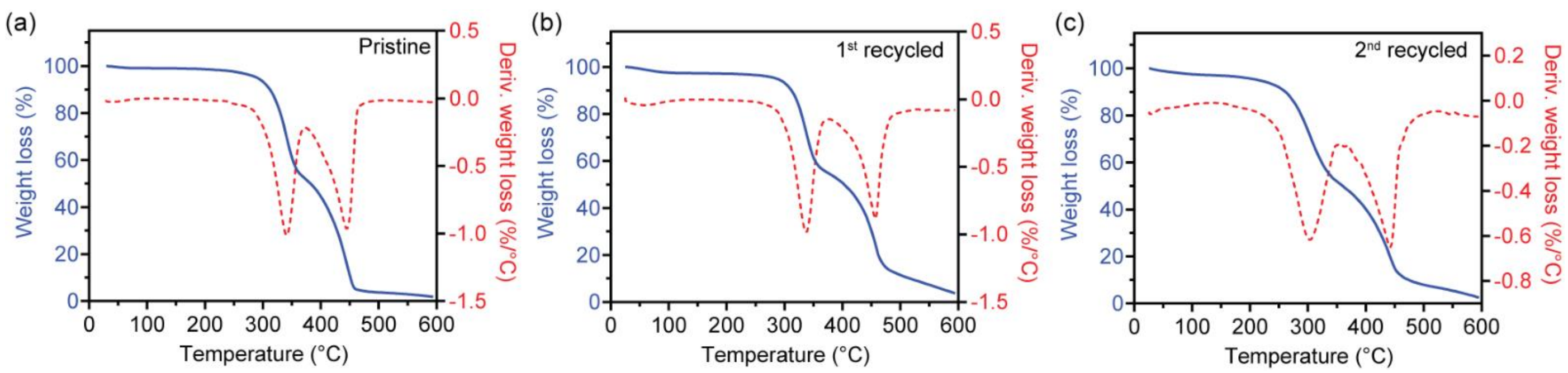

Figure S10. Thermogravimetric analysis (TGA) curves of pristine and recycled SPE membranes, respectively.

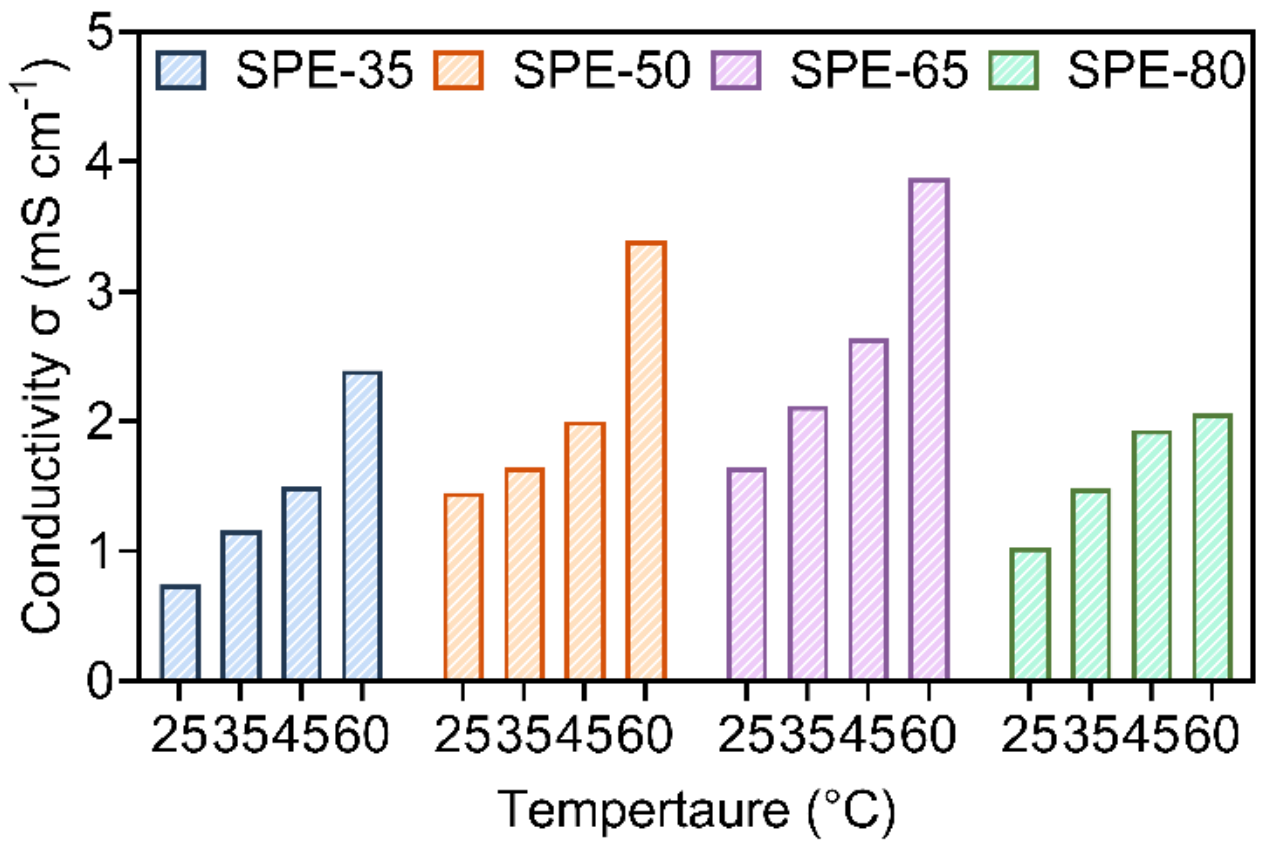

Figure S11. Temperature-dependent ionic conductivity of SPE membranes measured by electrochemical impedance spectroscopy during a heating cycle from 25 to 60 °C. The ionic conductivity increased with increasing temperature, indicating thermally enhanced ion transport within the SPE membranes.

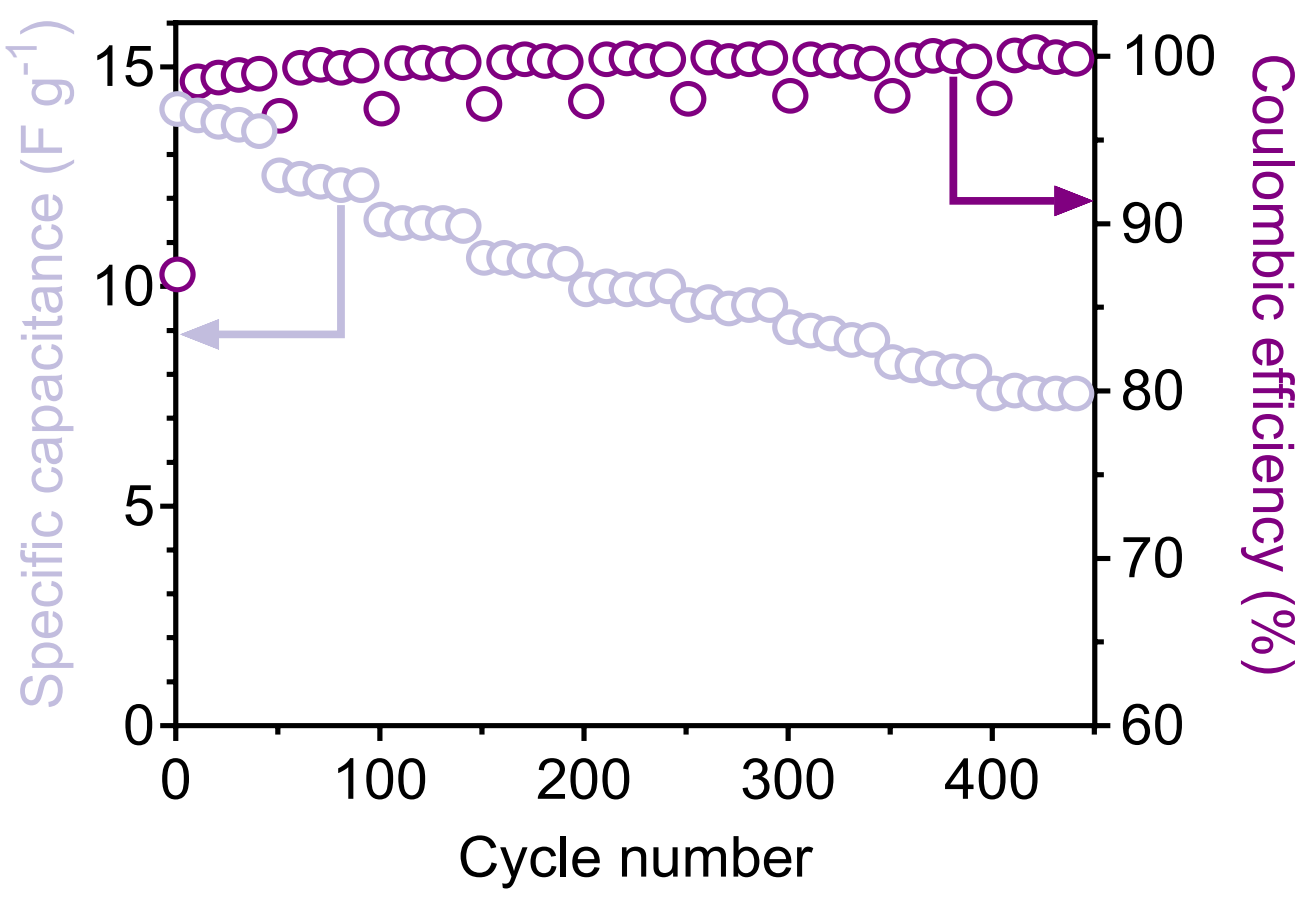

Figure S12. Specific capacitance (F g^{-1}) and Coulombic efficiency (%) of symmetric AC||SPE-65||AC supercapacitors measured at different current densities. The current density varied from 50 to 250 mA g^{-1} in 50 mA g^{-1} interval, with 50 cycles at each step, resulting in 450 total cycles.

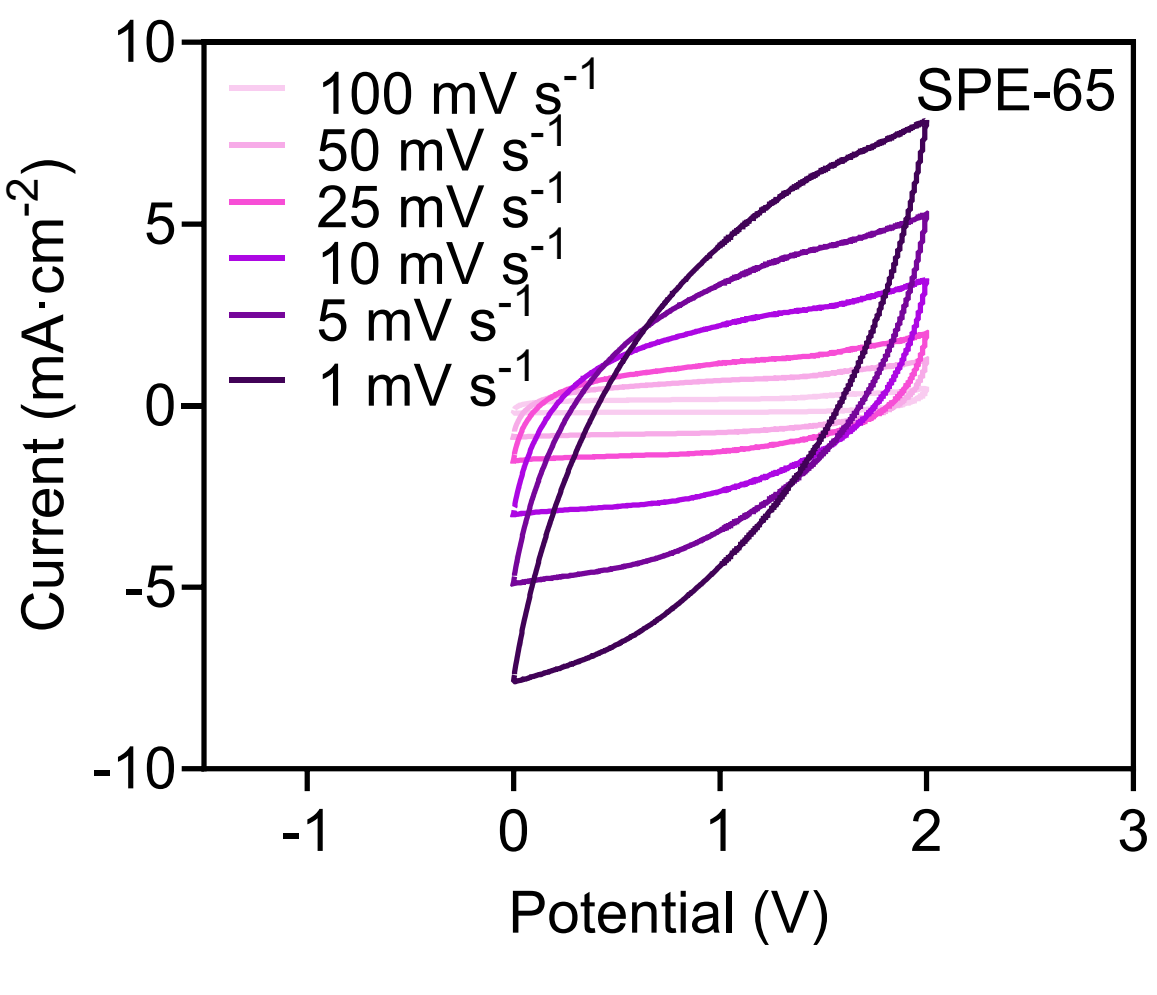

Figure S13. Cyclic voltammetry (CV) curve of symmetric AC||SPE-65||AC supercapacitor, with a scanning rate ranging from 1 mV s^{-1} to 100 mV s^{-1}.

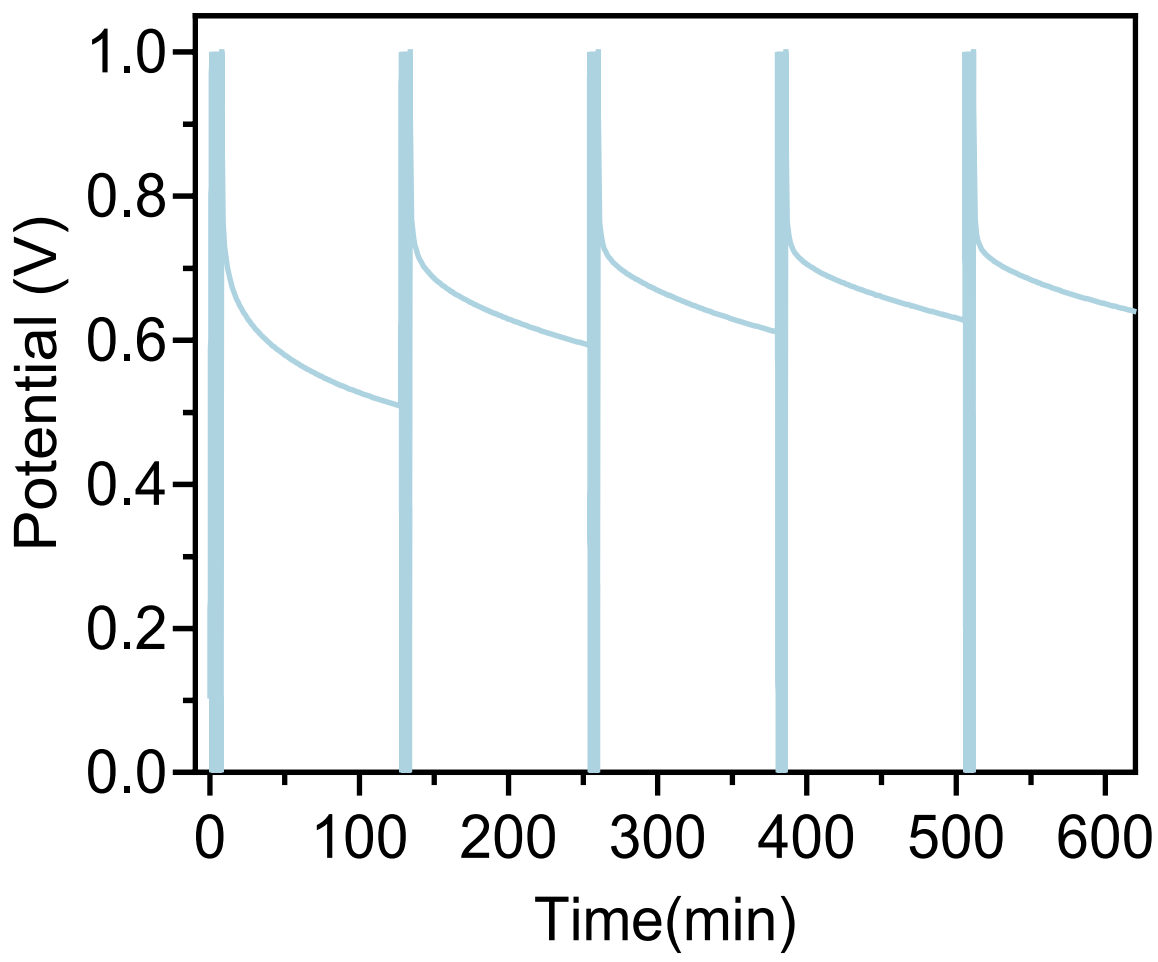

Figure S14. Accelerated electrochemical stability analysis for the symmetric AC||SPE-65||AC supercapacitor. Open-circuit voltage (OCV) decay test conducted after initial galvanostatic charge-discharge cycling at 250 mA g^{-1}.

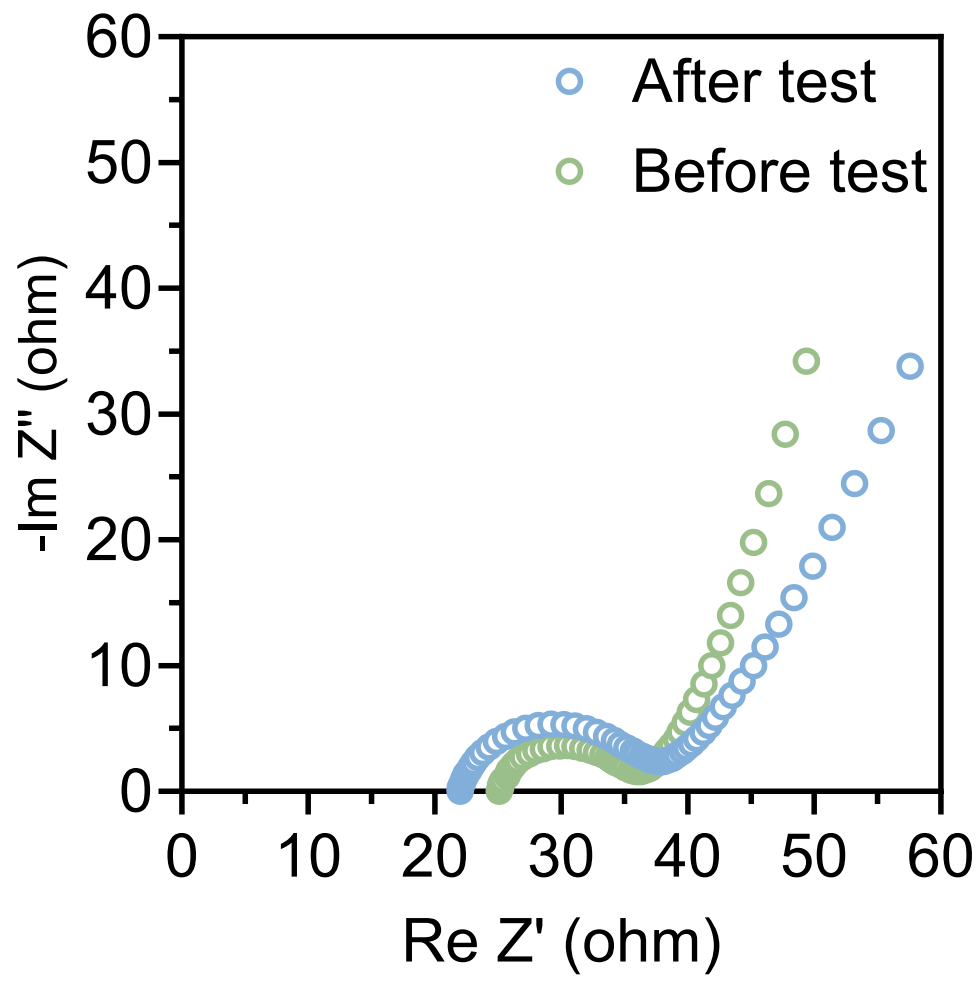

Figure S15. Nyquist plots for the AC||SPE-65||AC supercapacitor before and after the OCV test, demonstrating the sustained electrochemical stability of the SPE-65 electrolyte.

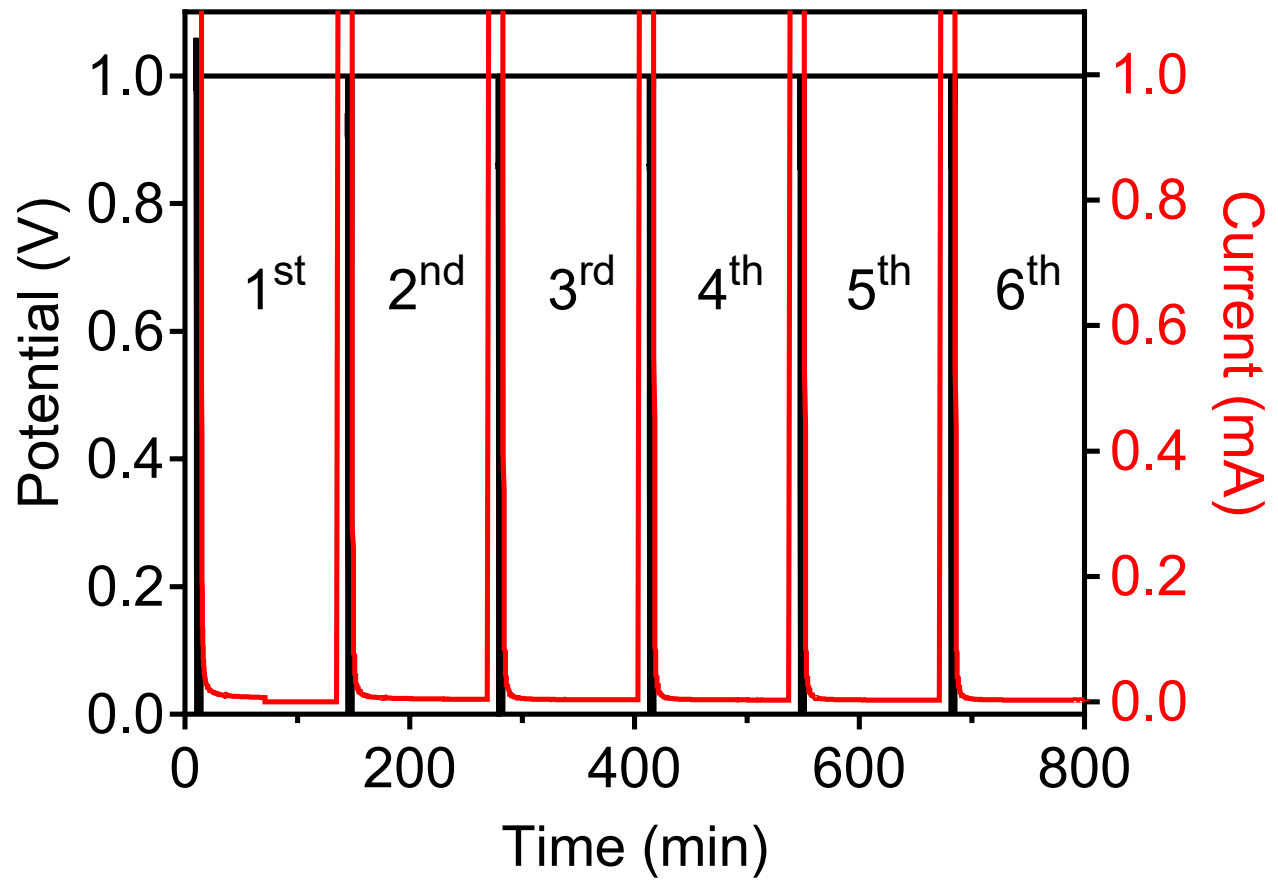

Figure S16. Accelerated electrochemical stability analysis for the symmetric AC||SPE-65||AC supercapacitor. Floating/holding profiles for the symmetric AC||SPE-65||AC supercapacitor at a peak voltage held of 1 V over six successive cycles, showing a low steady-state leakage current of approximately 50 µA.

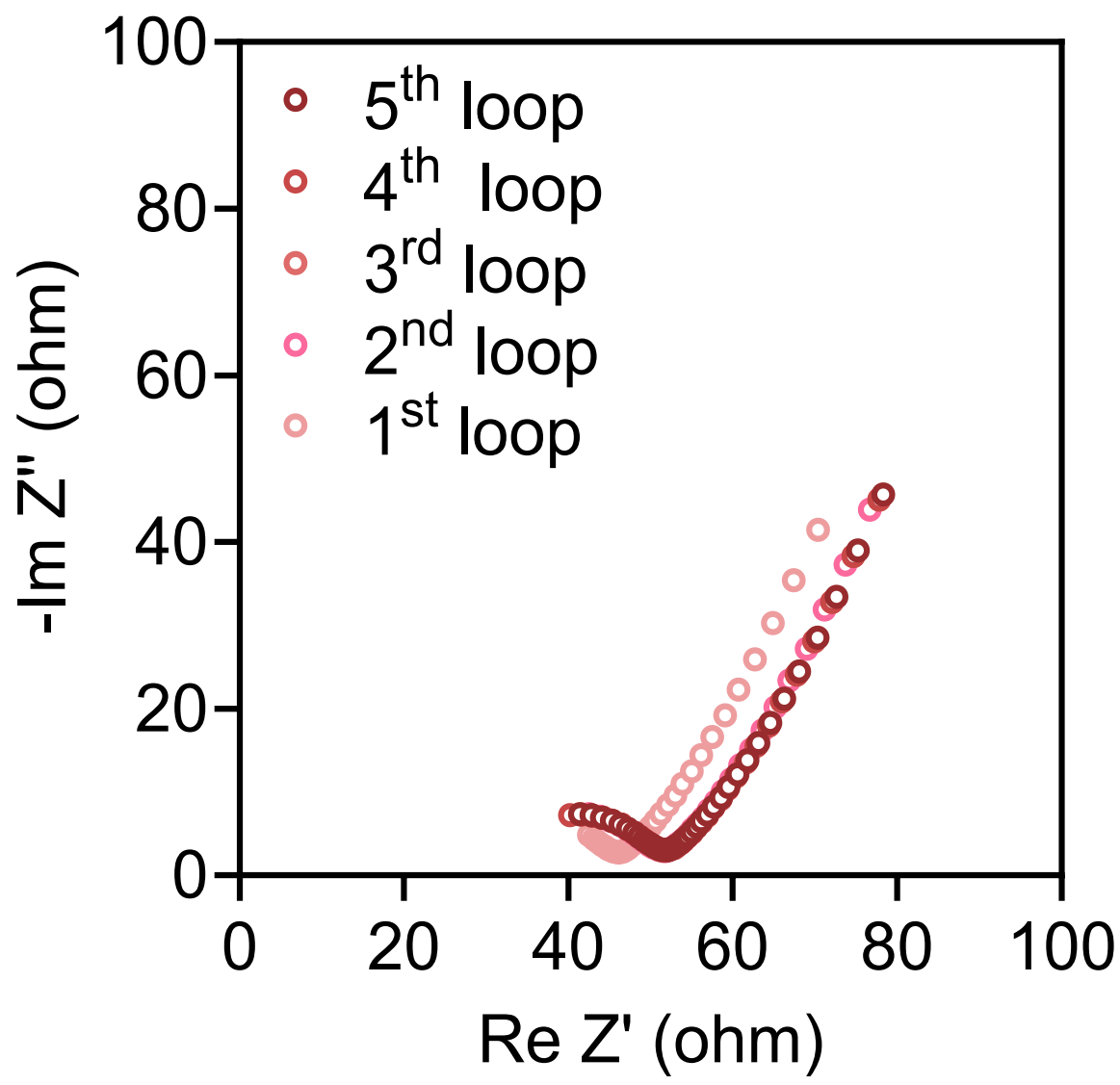

Figure S17. Nyquist plots for the symmetric AC||SPE-65||AC supercapacitor collected after each floating/holding cycle, demonstrating minimal changes in internal resistance and sustained electrochemical stability during repeated voltage holding.

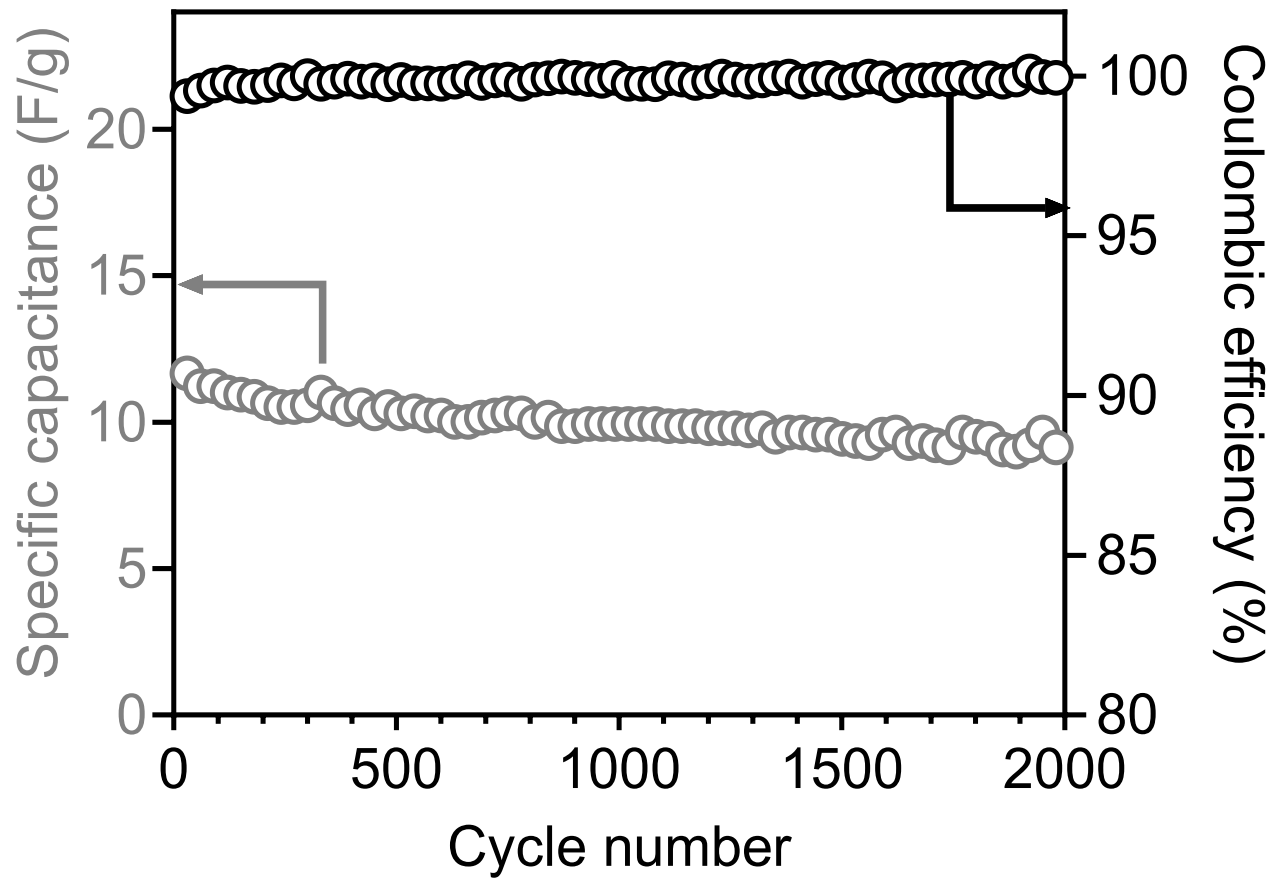

Figure S18. Specific capacitance (F g^{-1}) and Coulombic efficiency (%) of symmetric AC||SPE-20||AC supercapacitor at 100 mA g^{-1} current density, cycled in 1 V operating voltage window.

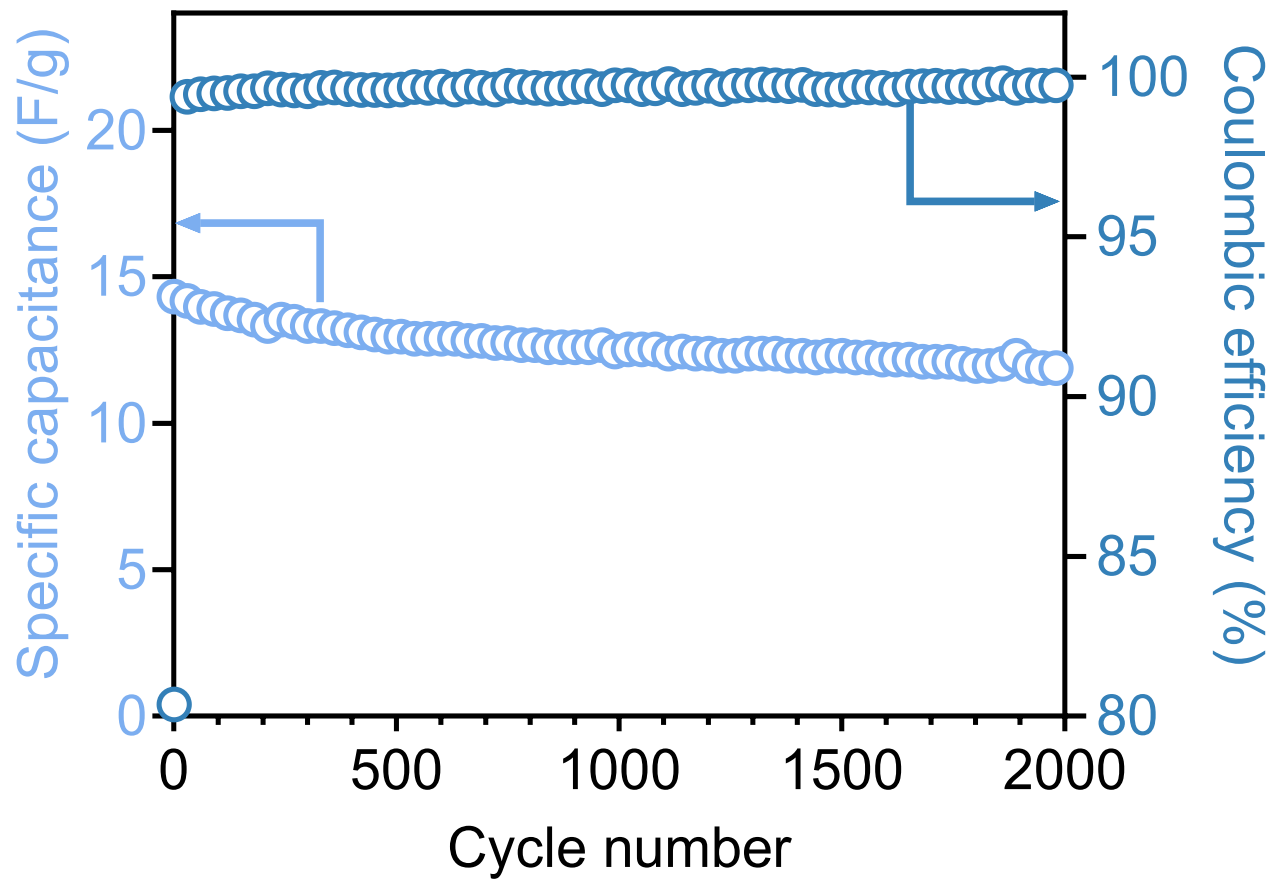

Figure S19. Specific capacitance (F g^{-1}) and Coulombic efficiency (%) of symmetric AC||SPE-35||AC supercapacitor at 100 mA g^{-1} current density, cycled in 1 V operating voltage window.

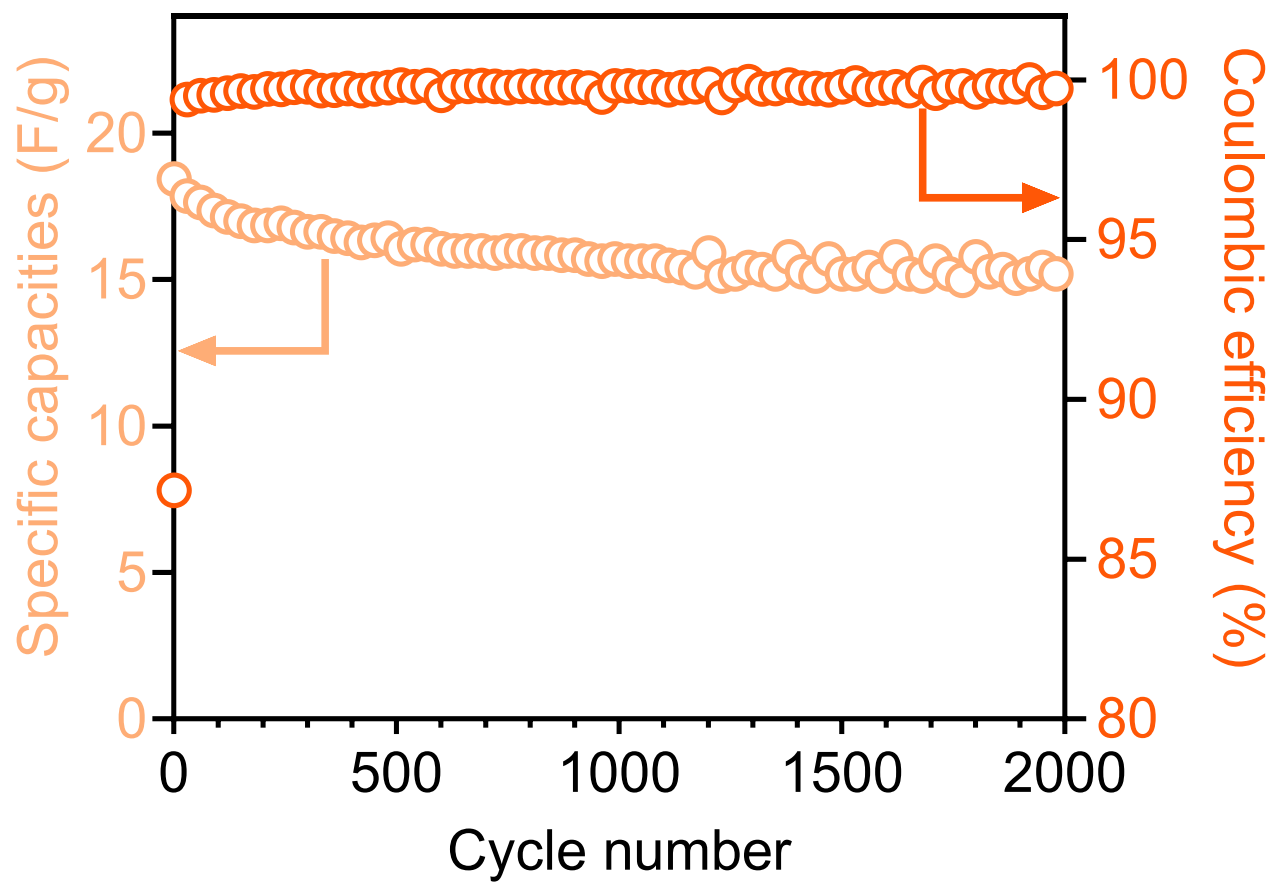

Figure S20. Specific capacitance (F g^{-1}) and Coulombic efficiency (%) of symmetric AC||SPE-50||AC supercapacitor at 100 mA g^{-1} current density, cycled in 1 V operating voltage window.

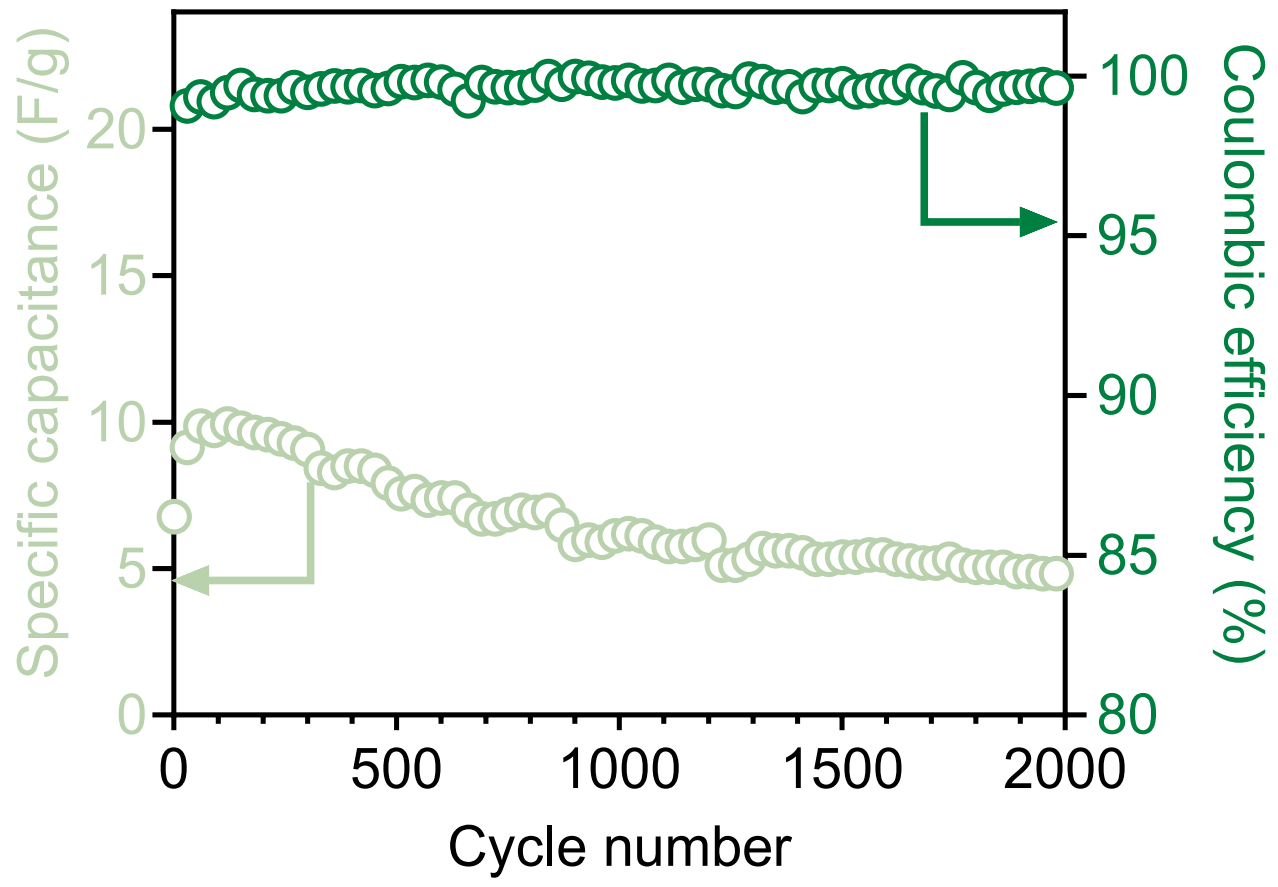

Figure S21. Specific capacitance (F g^{-1}) and Coulombic efficiency (%) of symmetric AC||SPE-80||AC supercapacitor at 100 mA g^{-1} current density, cycled in 1 V operating voltage window.

Table S3. Summary of samples with different molar ratio of PEG-LP_2 using different degradation agents.

	SPE-20	SPE-35	SPE-50	SPE-65	SPE-80
DBU	N	Y	Y	Y	Y
DBU/DDT mixture	N	Y	Y	Y	Y
TBP	N	N	Y	Y	Y

Note: Y(Yes): Sample can be fully degraded with specific depolymerization agents (solution can pass the SEC filter after depolymerization); N(No): Sample cannot be fully degraded with specific depolymerization agents (solution cannot pass the SEC filter after depolymerization).

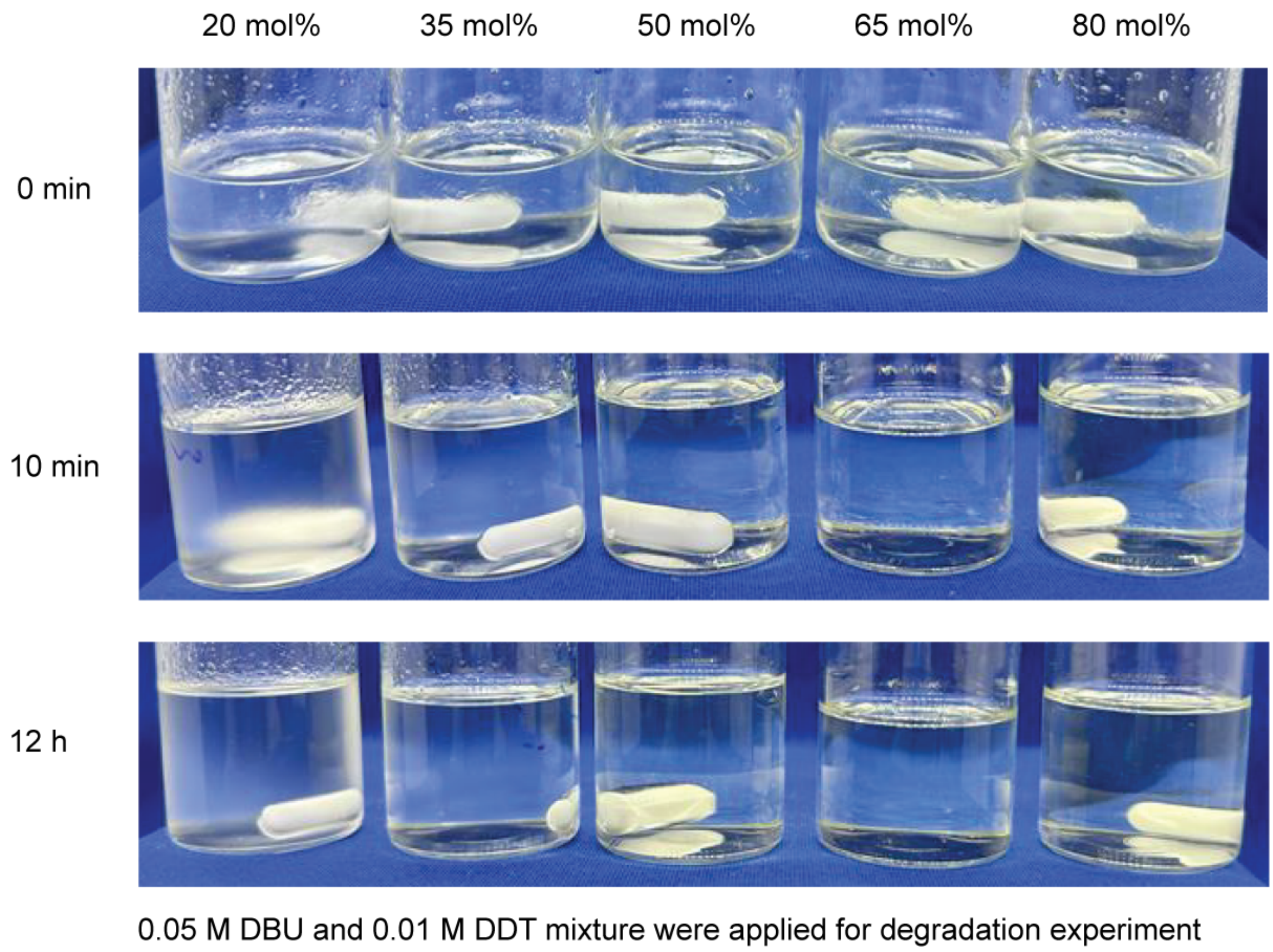

Figure S22. Photographs showing the dissolution of SPEs with varying PEG-LP_2 content in 0.05 M DBU and 0.01 M DDT in DCM, recorded at 0, 30, and 120 min.

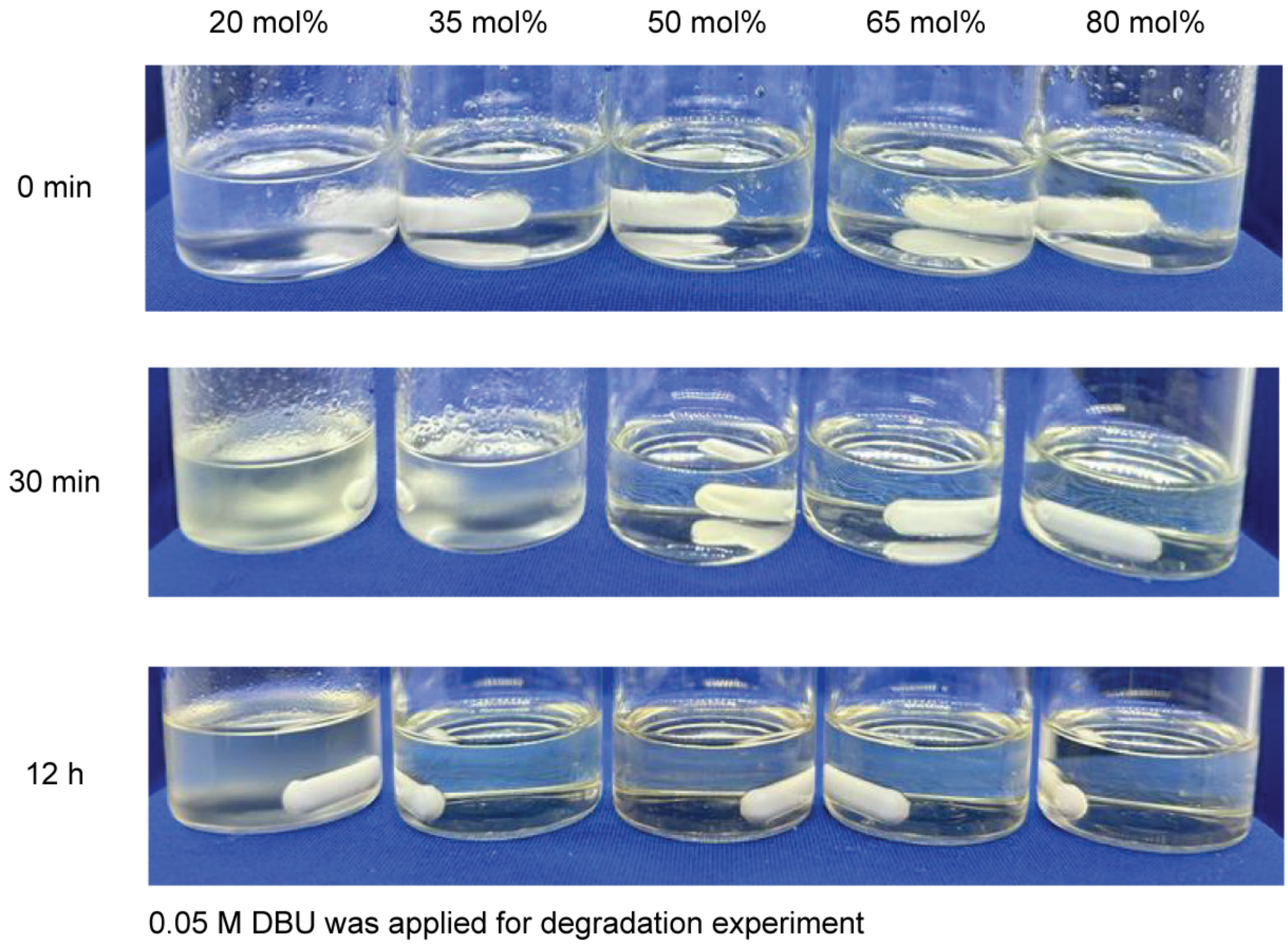

Figure S23. Photographs showing the dissolution of SPEs with varying PEG-LP_2 content in 0.05 M DBU in DCM, recorded at 0, 30, and 120 min.

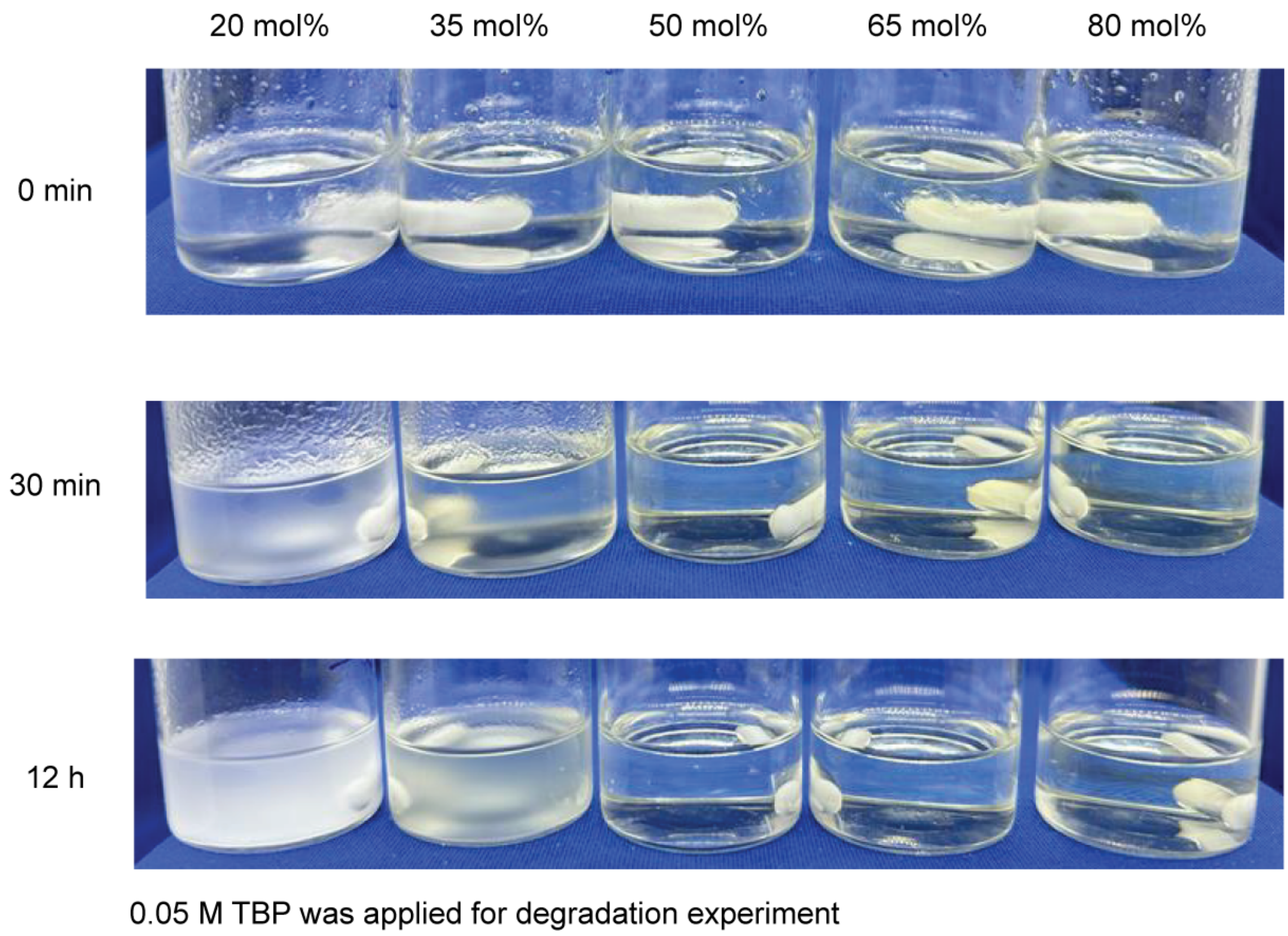

Figure S24. Photographs showing the dissolution of SPEs with varying PEG-LP_2 content in 0.05 M TBP in DCM, recorded at 0, 30, and 120 min.

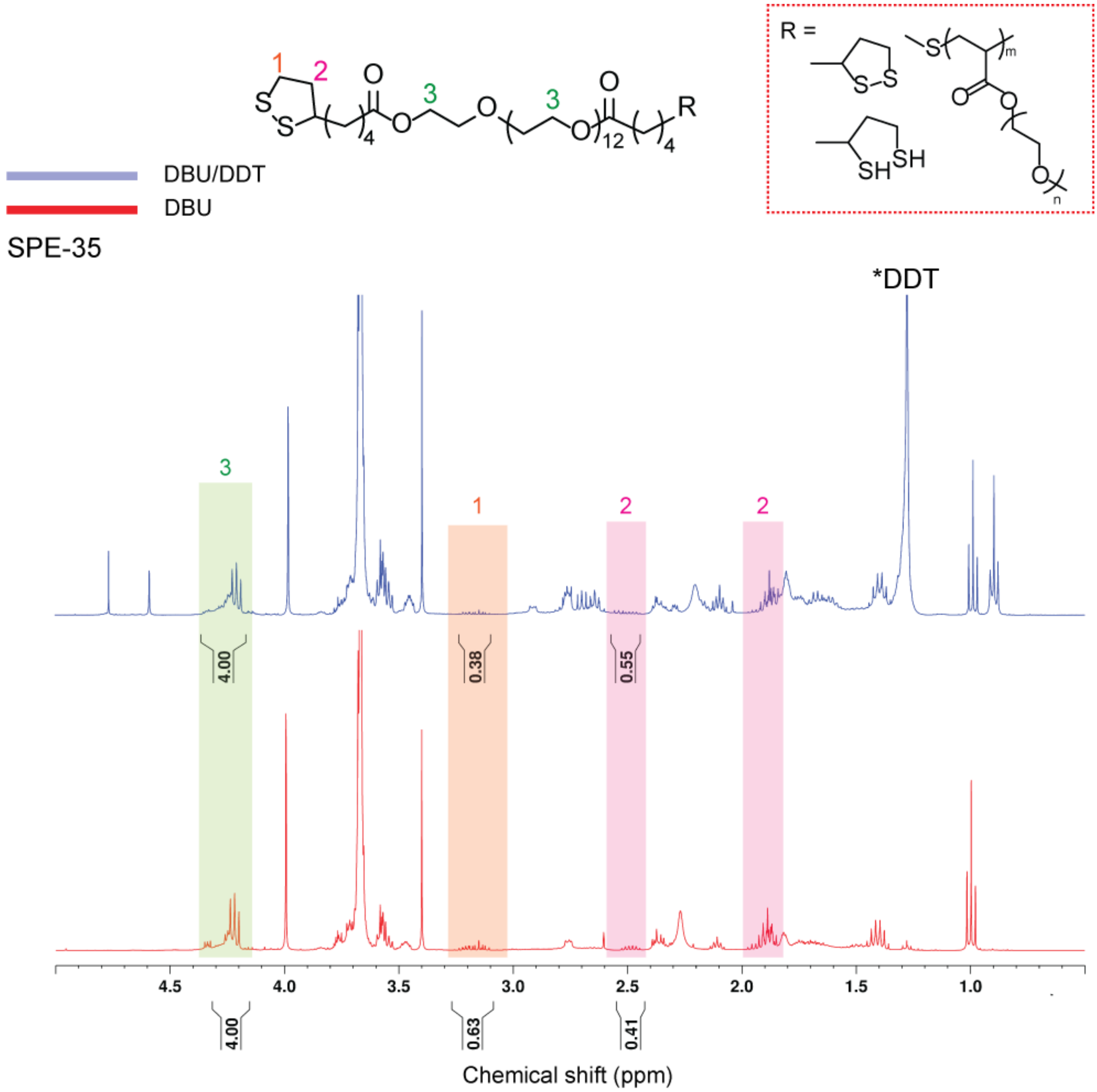

Figure S25. ^{1}H NMR spectra of depolymerization final purified product of SPE-35 using DBU and DDT mixture and pure DBU as depolymerization agents, respectively.

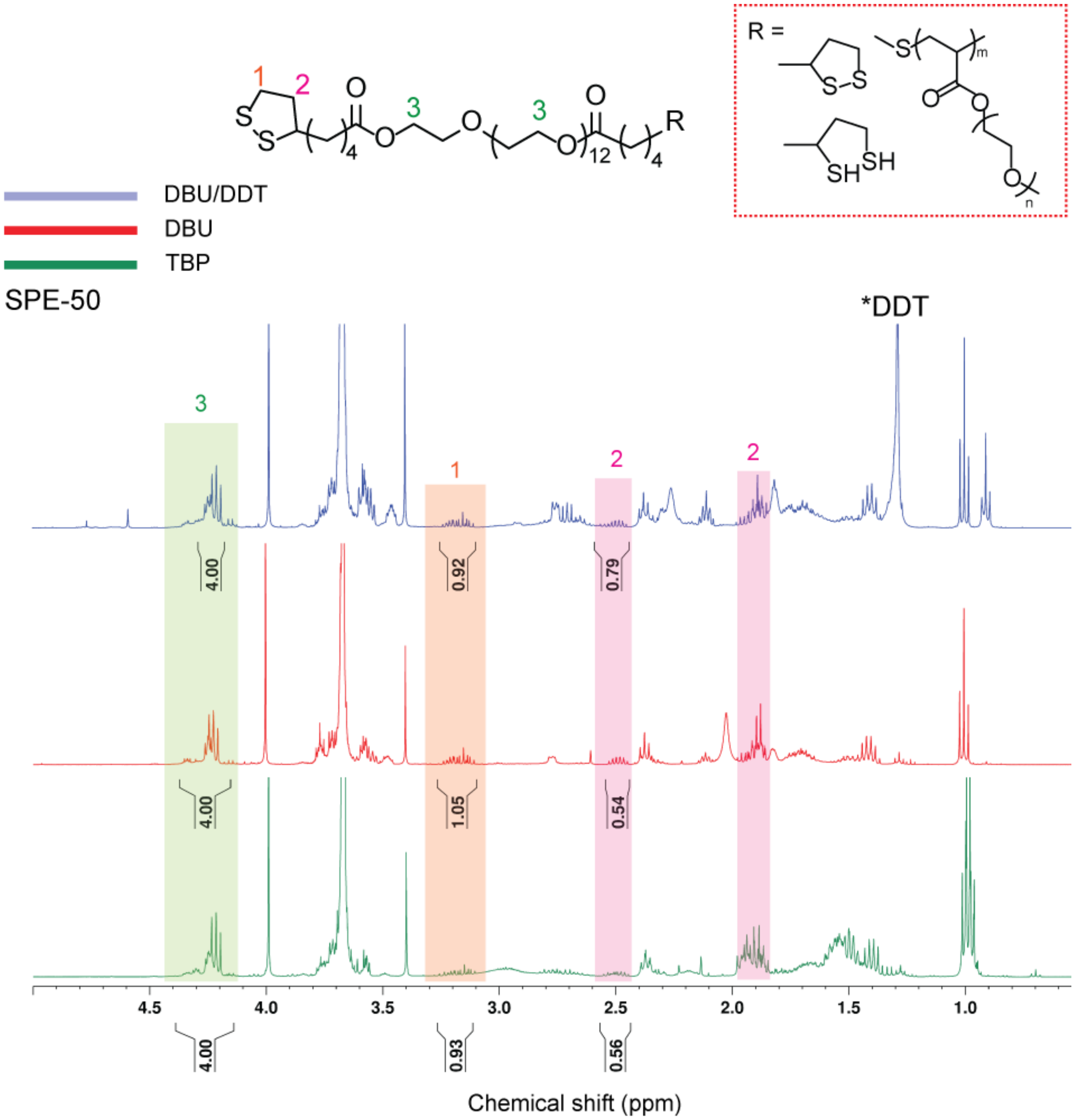

Figure S26. ^{1}H NMR spectra of depolymerization final purified product of SPE-50 using DBU and DDT mixture, pure DBU and TBP as depolymerization agents, respectively.

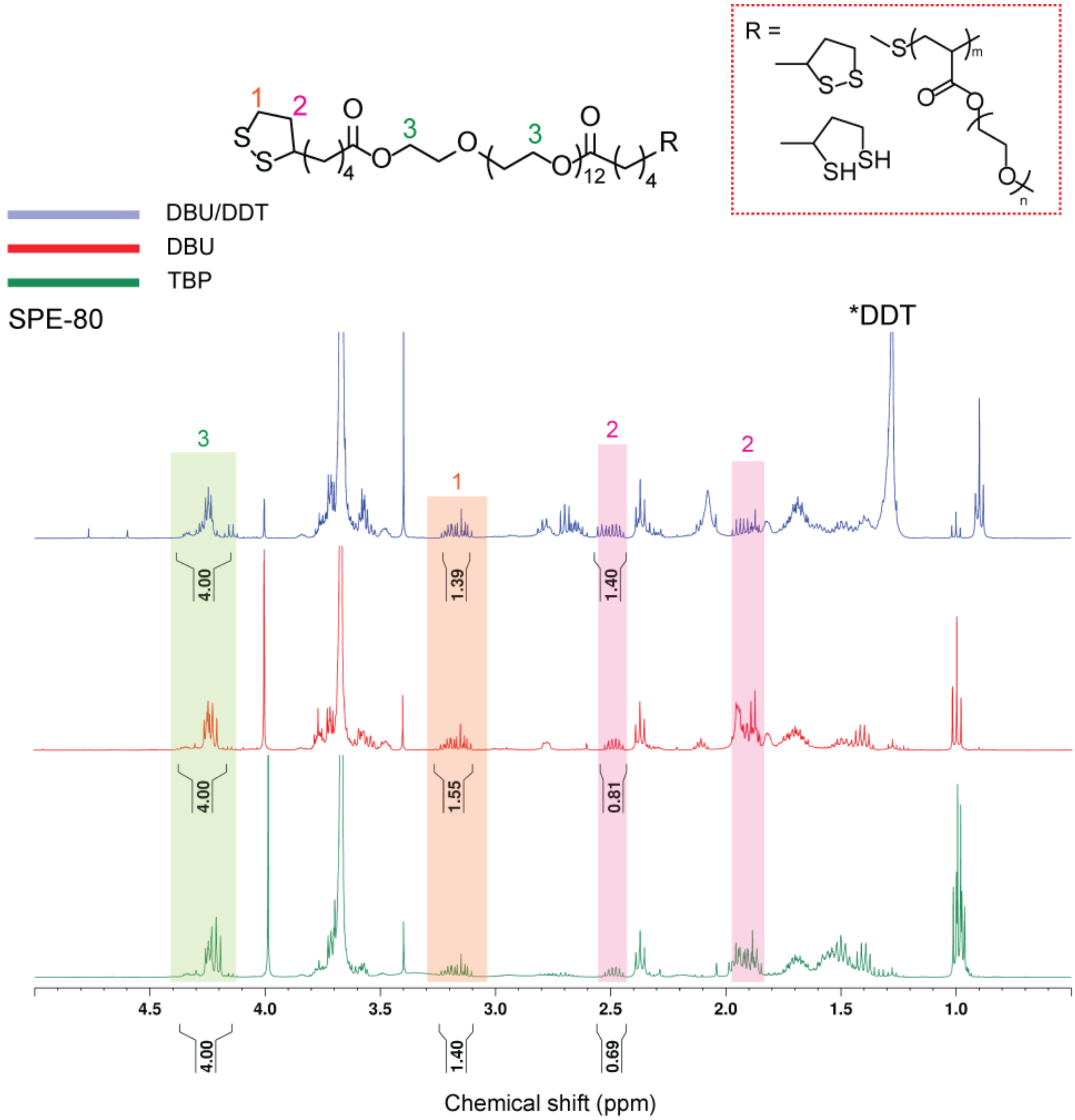

Figure S27. ^{1}H NMR spectra of depolymerization final purified product of SPE-80 using DBU and DDT mixture, pure DBU and TBP as depolymerization agents, respectively.

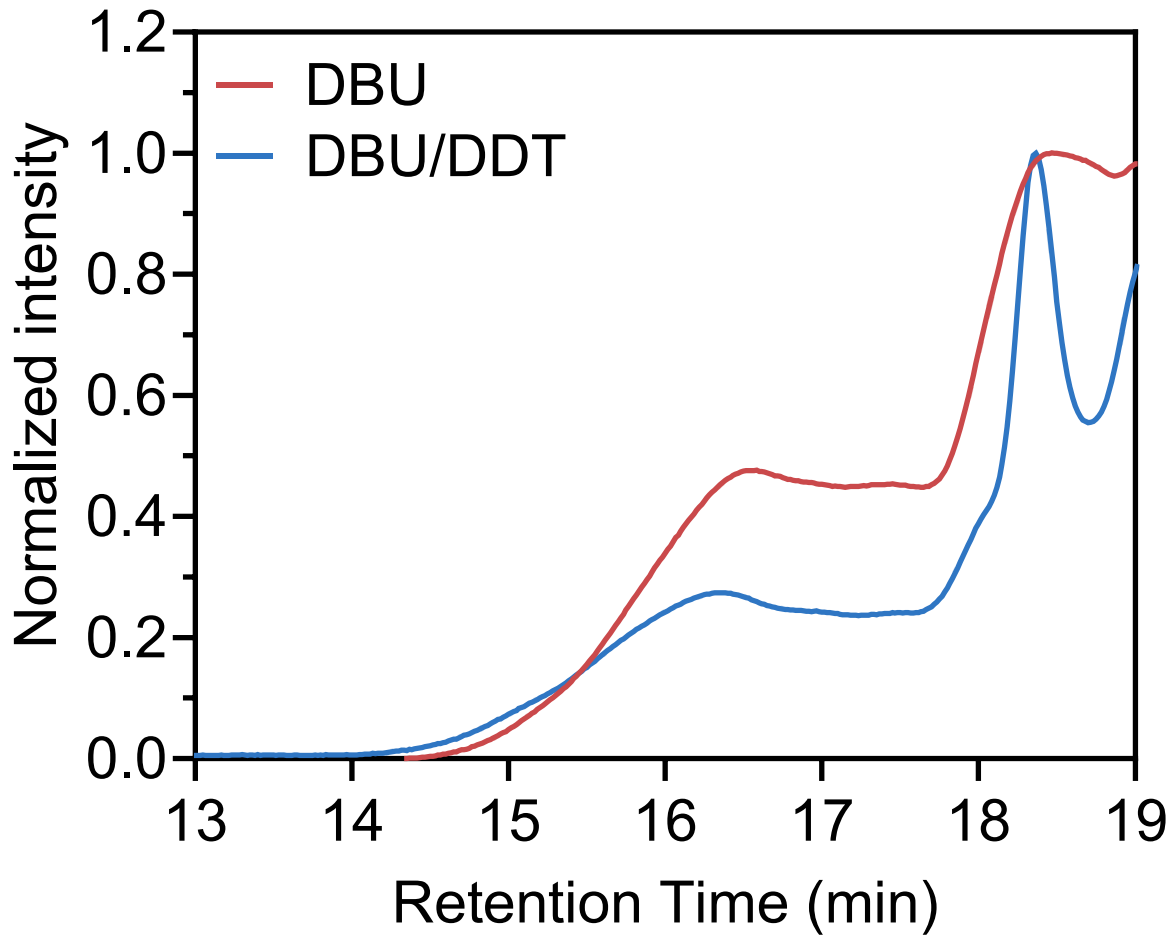

Figure S28. SEC curves of final depolymerization product of SPE-35 using DBU and DDT mixture and pure DBU, respectively.

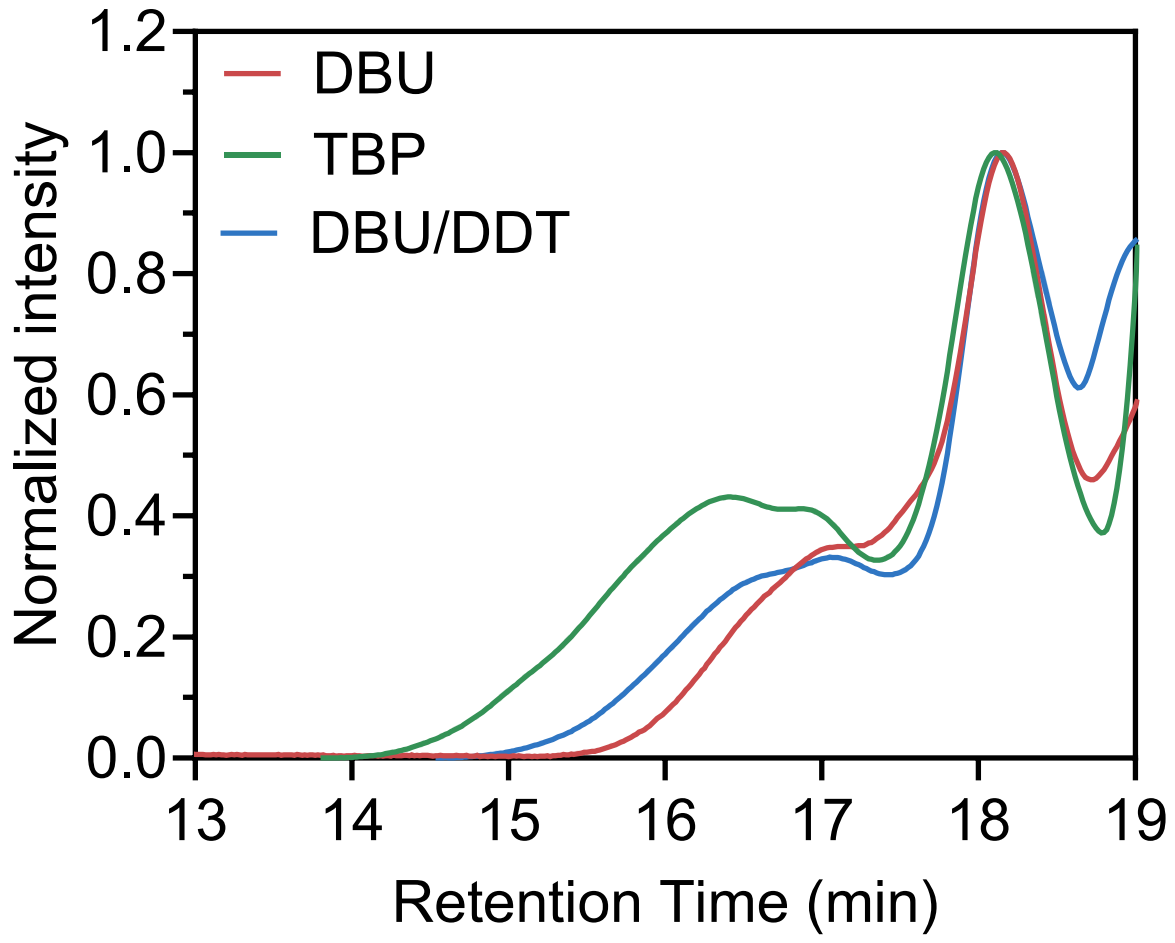

Figure S29. SEC curves of depolymerization final product of SPE-50 using DBU and DDT mixture, pure DBU and TBP, respectively.

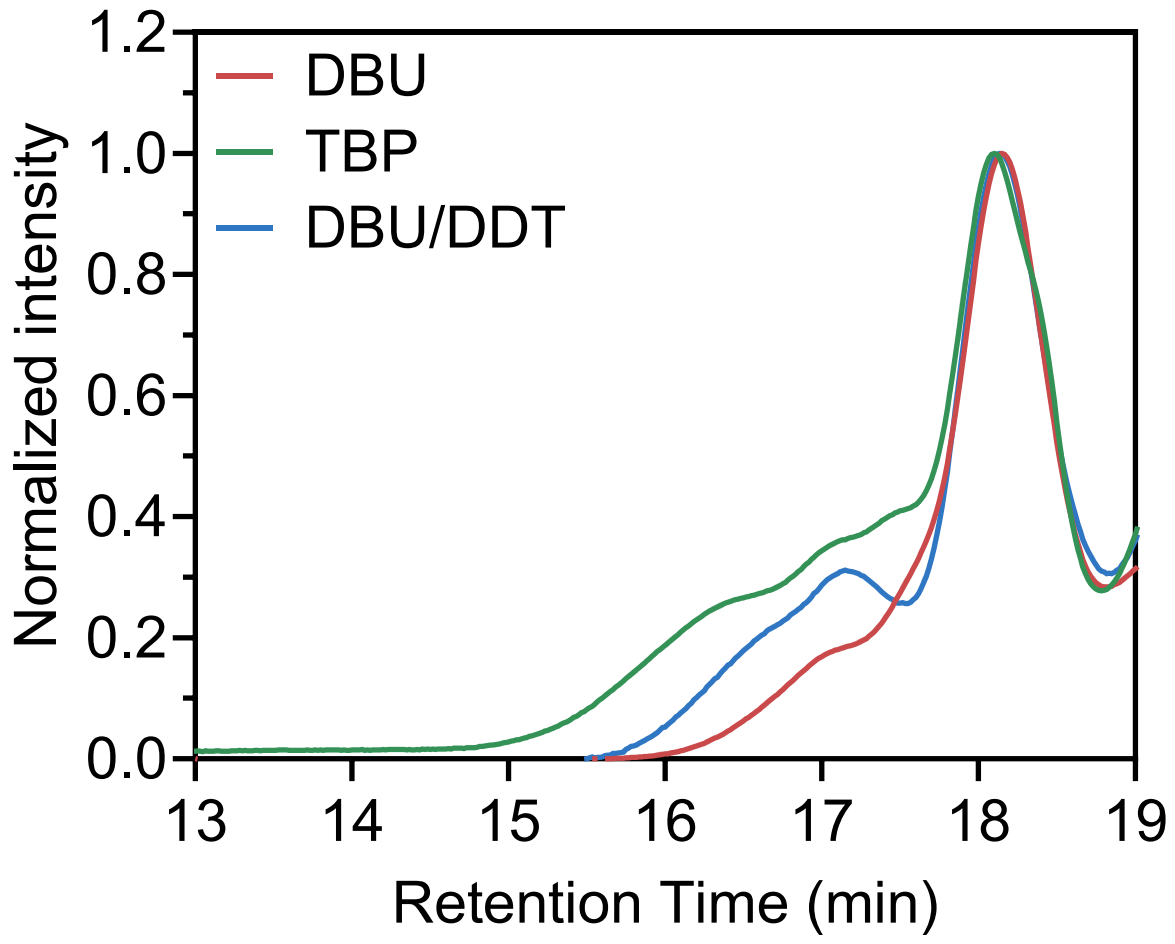

Figure S30. SEC curves of depolymerization final product of SPE-80 using DBU and DDT mixture, pure DBU and TBP, respectively.

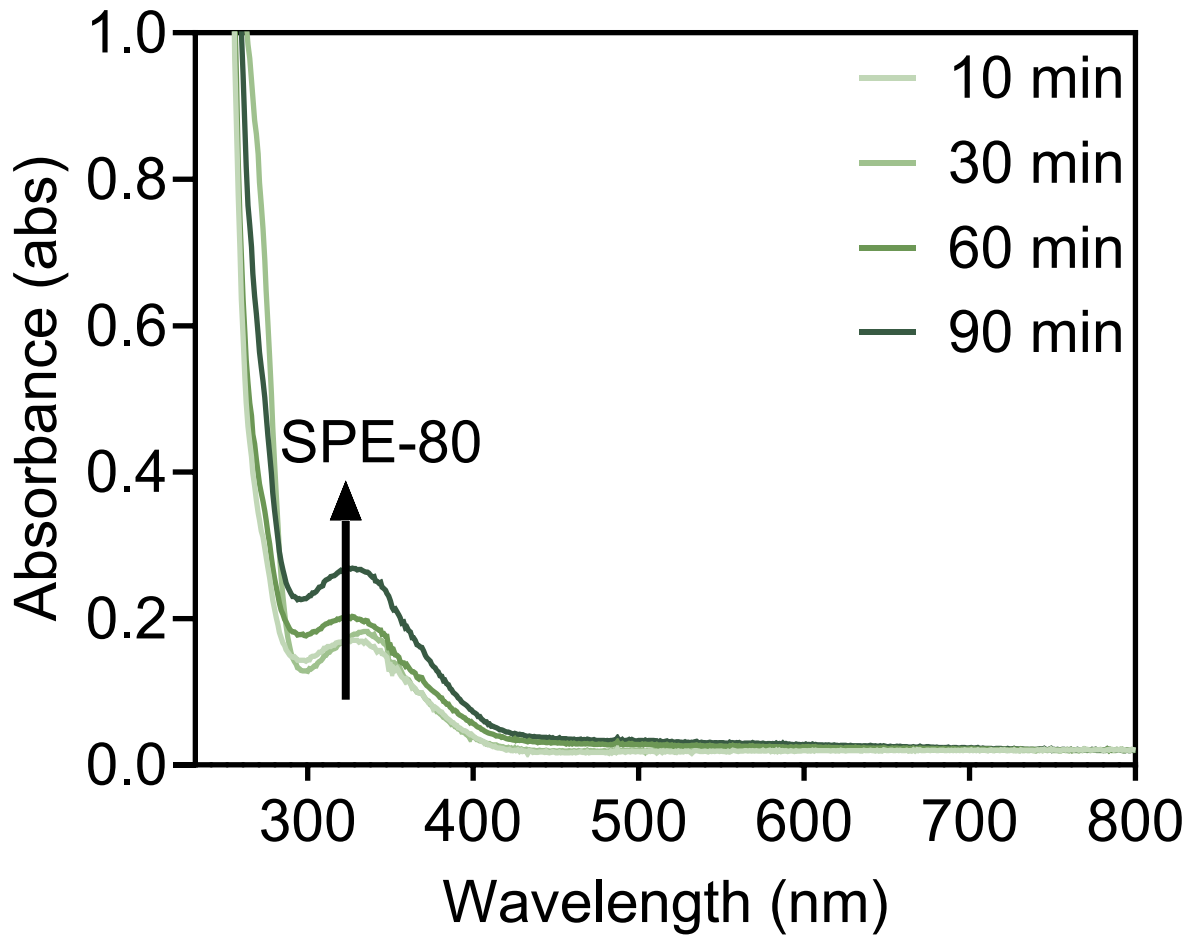

Figure S31. Degradation experiment with DBU monitoring by UV-Vis spectroscopy: SPE-80, with aliquots taken by 10 min, 30 min, 60 min, and 90 min, respectively.

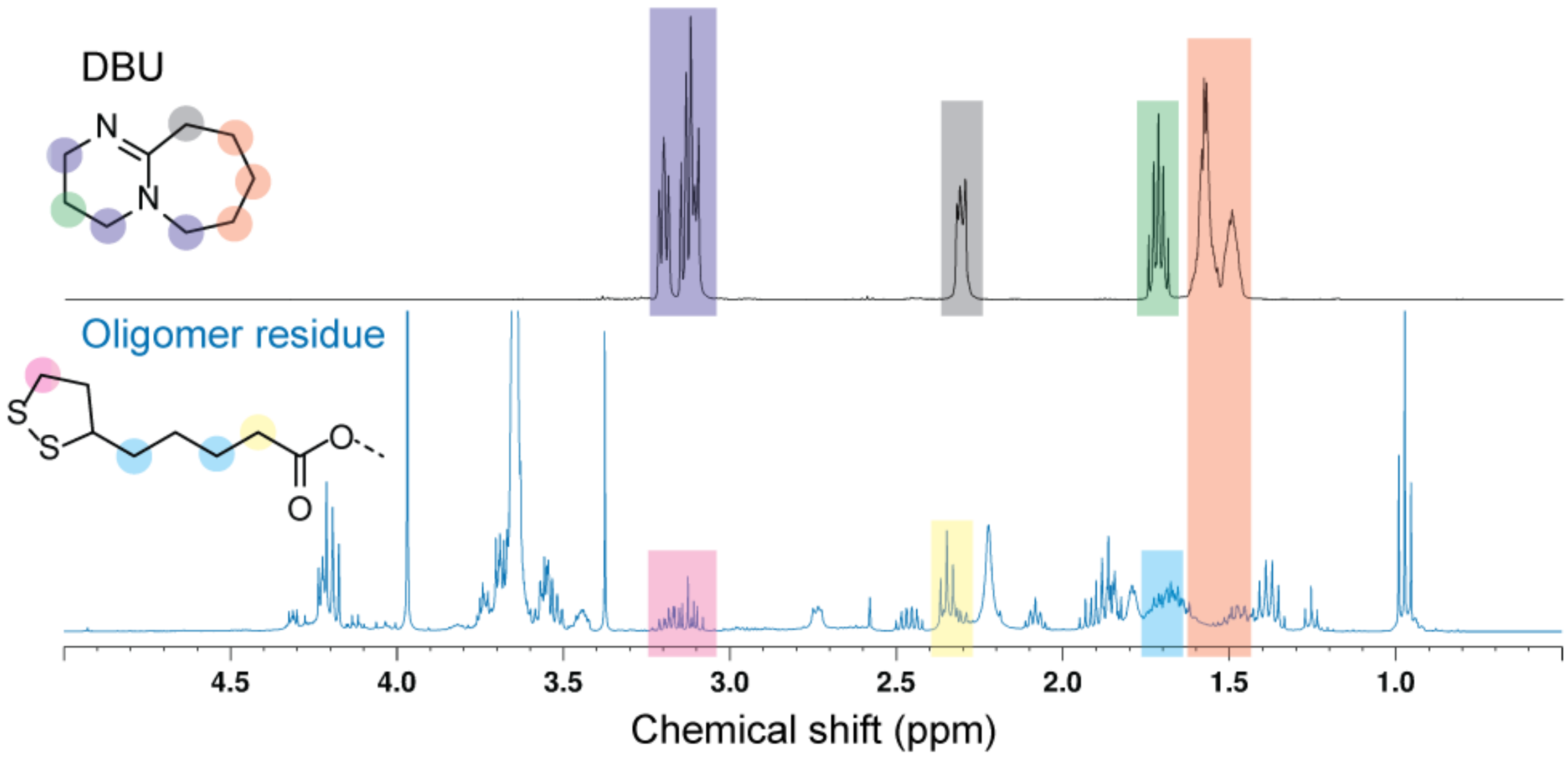

Figure S32. ^{1}H NMR of DBU, 1st depolymerization SPE-65 residue, respectively, showing the DBU was completely removed after recovery (the highlighted region in DBU remain unidentified in oligomer residue). Note: DBU peaks at 3.2 ppm and 1.7 ppm overlaps with lipoate signals.

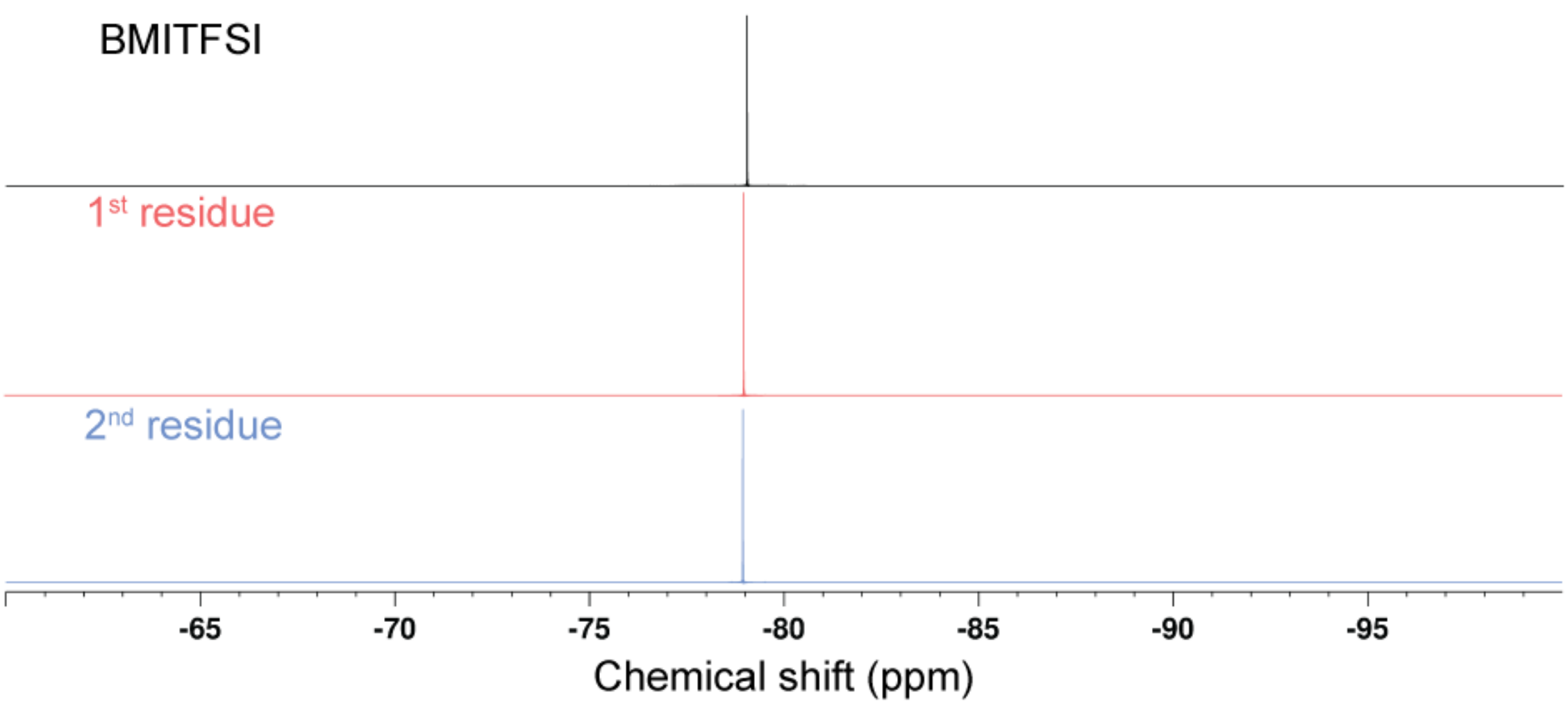

Figure S33. ^{19}F NMR of ionic liquid BMITFSI, 1st depolymerization residue, and 2nd depolymerization residue in $CDCl_3$, respectively.

Table S4. Comparison of pristine and recycled membranes using SPE-65 formulation.

Samples	OEGMEA	PEG-LP$_2$	BMITFSI	TPO	Curing time	Conductivity (mS cm^{-1})
SPE-65 pristine	35 mol%	65 mol%	45 wt%	2 wt%	3 min	1.30 ± 0.11
SPE-65 1st recycled	Extra 10 wt%	-	-	Extra 2 wt%	7 min	0.86 ± 0.12
SPE-65 1st recycled	Extra 30 wt%	-	-	Extra 2 wt%	5 min	1.21 ± 0.11
SPE-65 2nd recycled	Extra 10 wt%	-	-	Extra 2 wt%	20 min	0.71 ± 0.03

Note: Weight percentage of OEGMEA plus that of PEG-LP$_2$ is 55 wt % in total.

Table S5. Percentage of ionic liquid retention during each depolymerization/repolymerization process.

	Pristine	1st recycled	2nd recycled
Mass percentage of ionic liquid (%)	48.2	41.5	37.6
Ionic liquid retention (%)	-	86.1	90.6
Mass percentage of polymer (%)	44.7	40.1	37.2
Polymer retention (%)	-	89.7	92.7

The ionic liquid retention after each depolymerization/repolymerization cycle was estimated from the TGA curves by integrating the mass-loss step corresponding to BMITFSI decomposition. Based on the derivative thermogravimetry (DTG) curves, the higher-temperature degradation peak from approximately 370 °C to 490 °C was attributed to BMITFSI decomposition. Therefore, the mass loss associated with the specific degradation stage was used to estimate the BMITFSI content in each membrane. The ionic liquid retention was calculated using the **Equation S3** below:

$$IL_{retention} = \frac{w_{IL,n}}{w_{IL,n-1}} \times 100\% \quad (3)$$

where $w_{\mathrm{IL,n}}$ and $w_{\mathrm{IL,n-1}}$ are the BMITFSI contents calculated from the corresponding TGA mass-loss steps of the nth cycles and n-1th cycles of SPE-65 membranes, respectively.

Similarly, the cross-linked polymer retention after recovery was also evaluated using the same calculations. Based on the DTG curves, the lower-temperature degradation peak from approximately 270 °C to 370 °C was attributed to cross-linked polymer network decomposition. Therefore, the mass loss associated with the specific degradation stage was used to estimate the cross-linked polymer network in each membrane. The polymer/oligomer retention was calculated using the **Equation S4** below:

$$Polymer_{retention} = \frac{w_{poly,n}}{w_{poly,n-1}} \times 100\% \quad (4)$$

where $w_{poly,n}$ and $w_{poly,n-1}$ are the polymer contents calculated from the corresponding TGA mass-loss steps of the nth cycles and n-1th cycles of SPE-65 membranes, respectively.

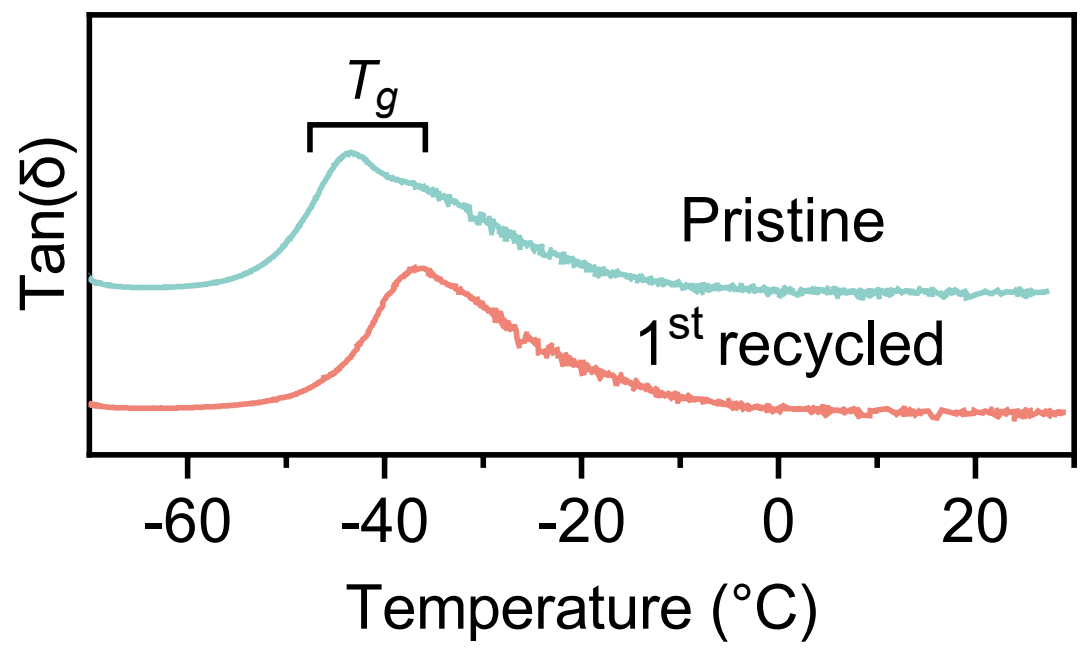

Figure S34. Tan(δ) as a function of temperature for pristine SPE-65 and 1st recycled SPE-65 membranes, respectively.

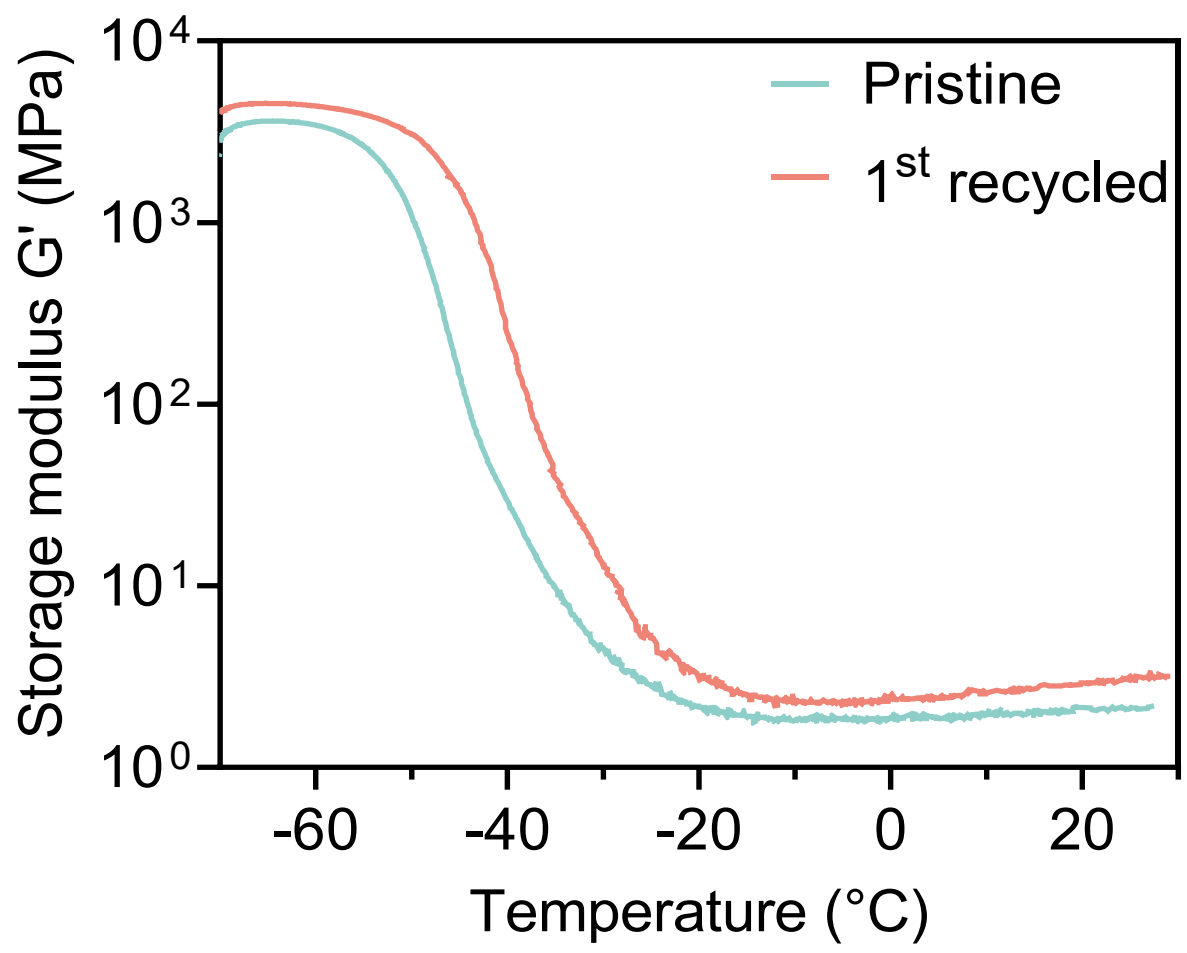

Figure S35. Storage modulus as a function of temperature of pristine SPE-65 and 1st recycled SPE-65 membranes, respectively.

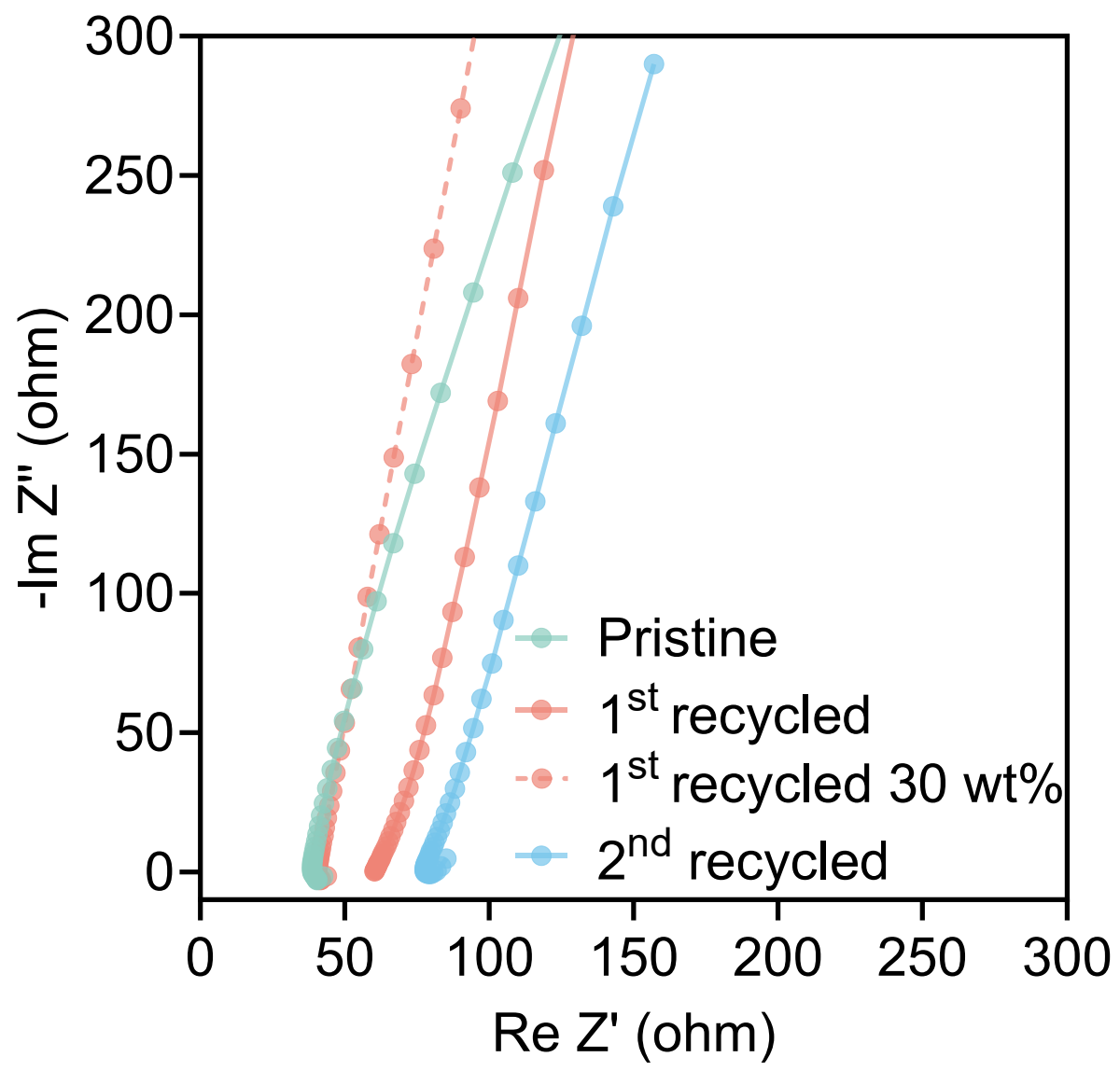

Figure S36. Nyquist impedance plots of SPE-65 pristine and recycled membranes at room temperature. Note: Membrane resistance gradually increased as the number of recycling cycles increased.

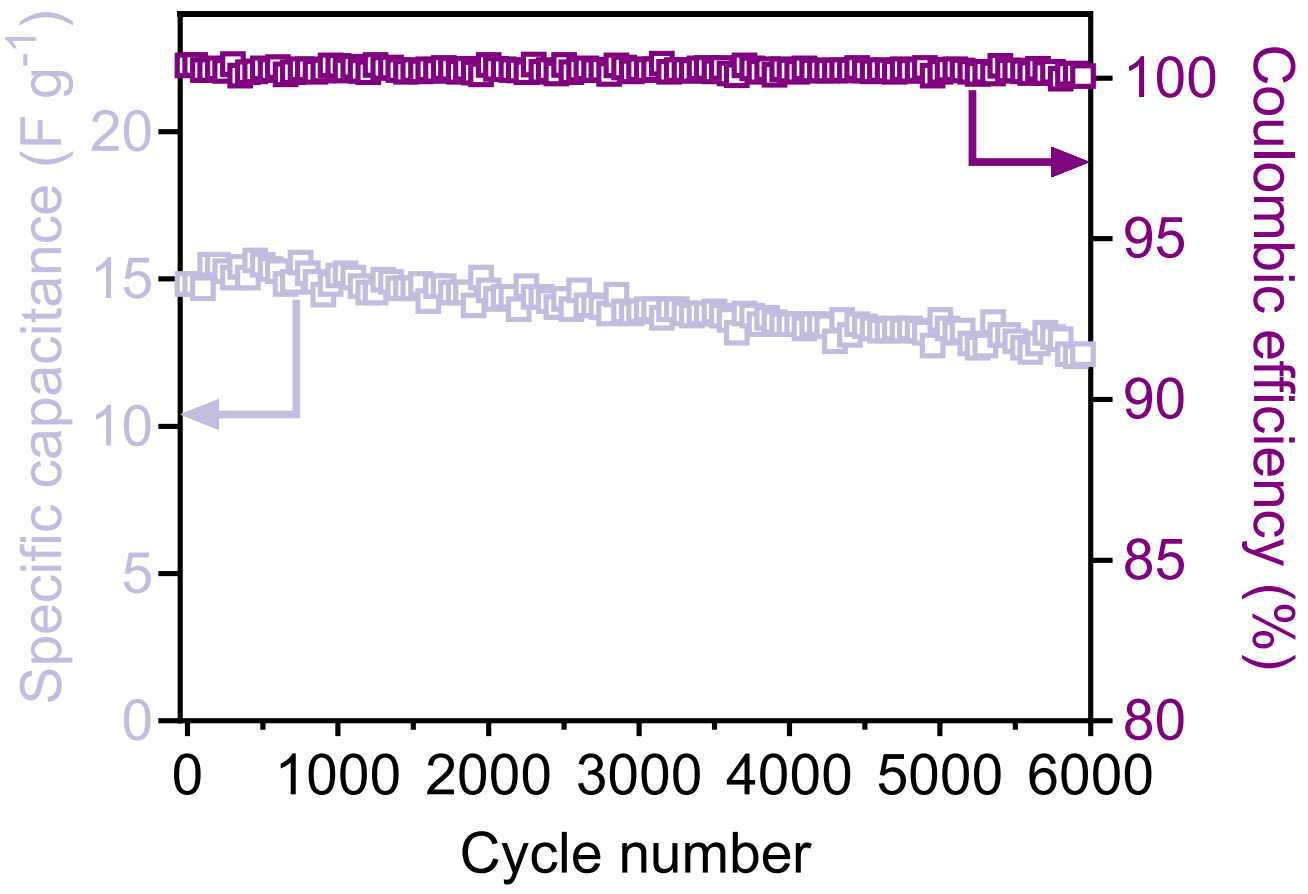

Figure S37. Specific capacitance (SC) and Coulombic efficiency (CE) of symmetric AC||1st recycled SPE-65||AC supercapacitor, respectively, with addition of 30 wt% extra monomer. SC of the cell retained 86% over 6 000 cycles.

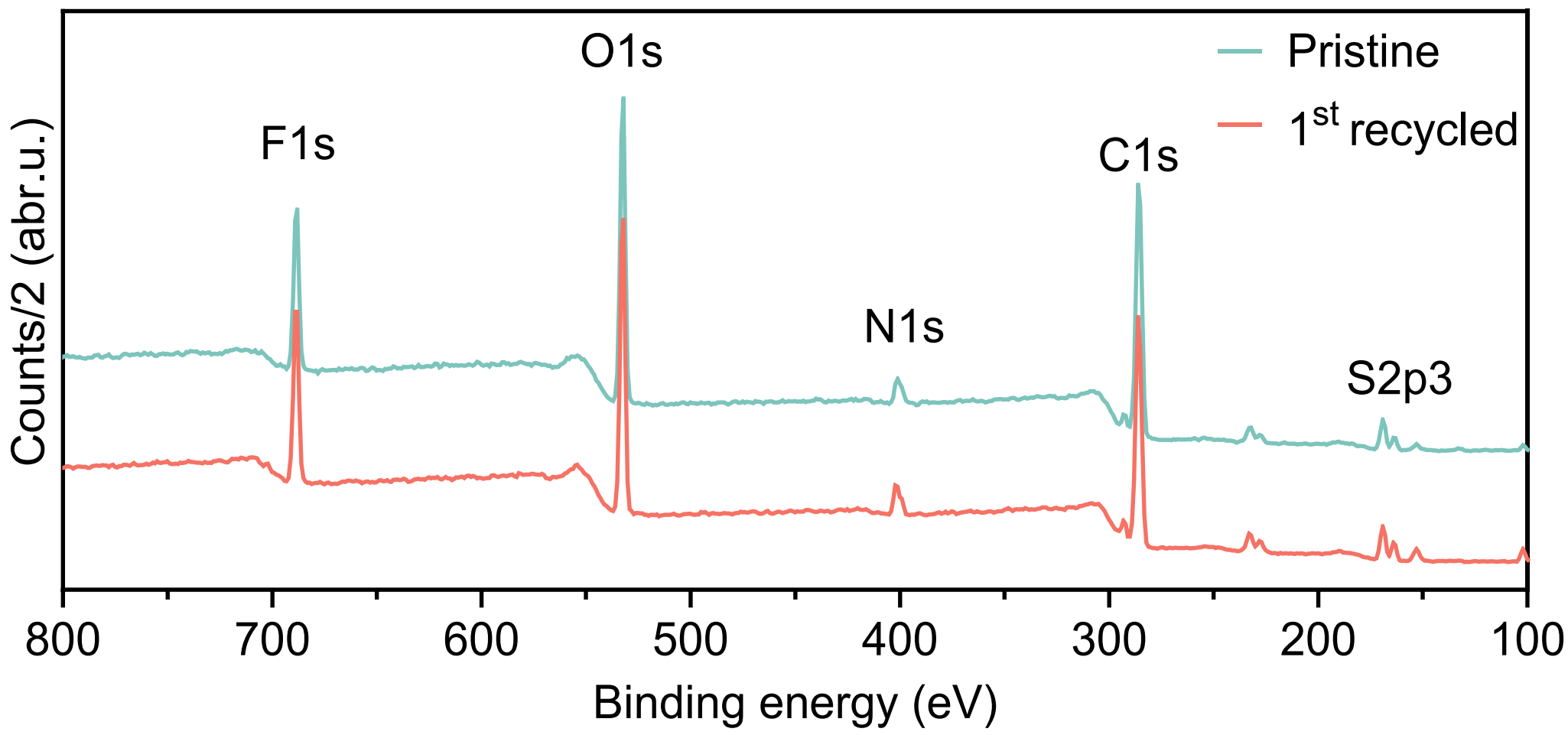

Figure S38. The surface XPS spectra of pristine and 1st recycled SPE-65 membrane, showing F1s, O1s, N1s, C1s, and S2p3 regions, respectively.

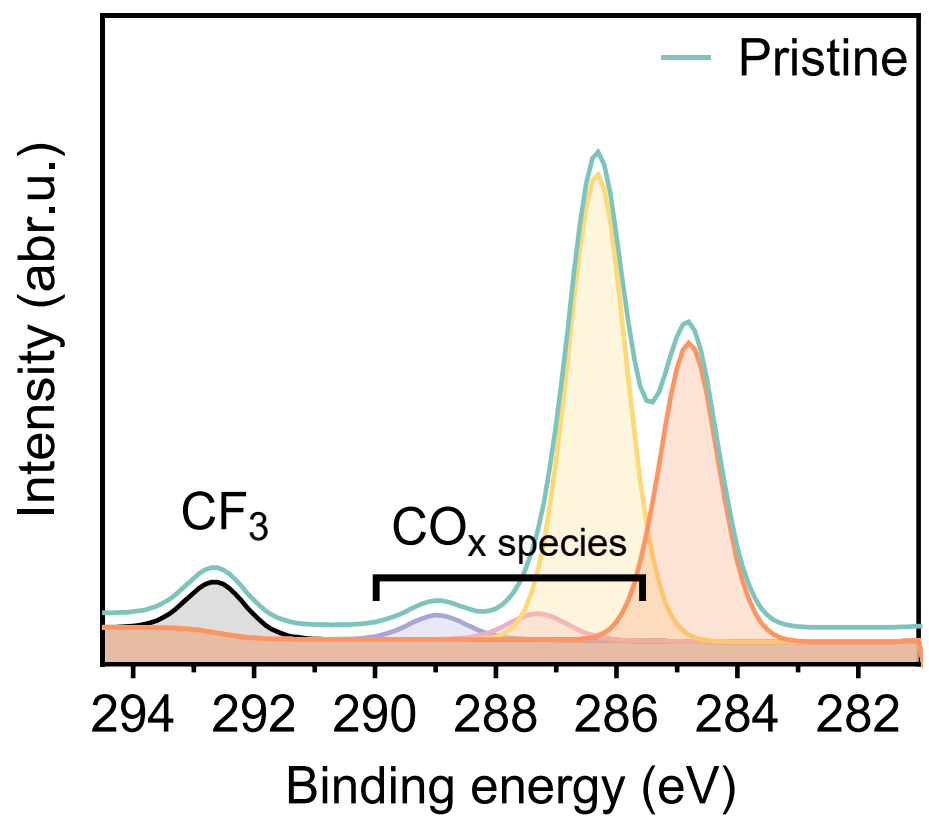

Figure S39. The surface C 1s XPS spectrum for pristine SPE-65 membrane.

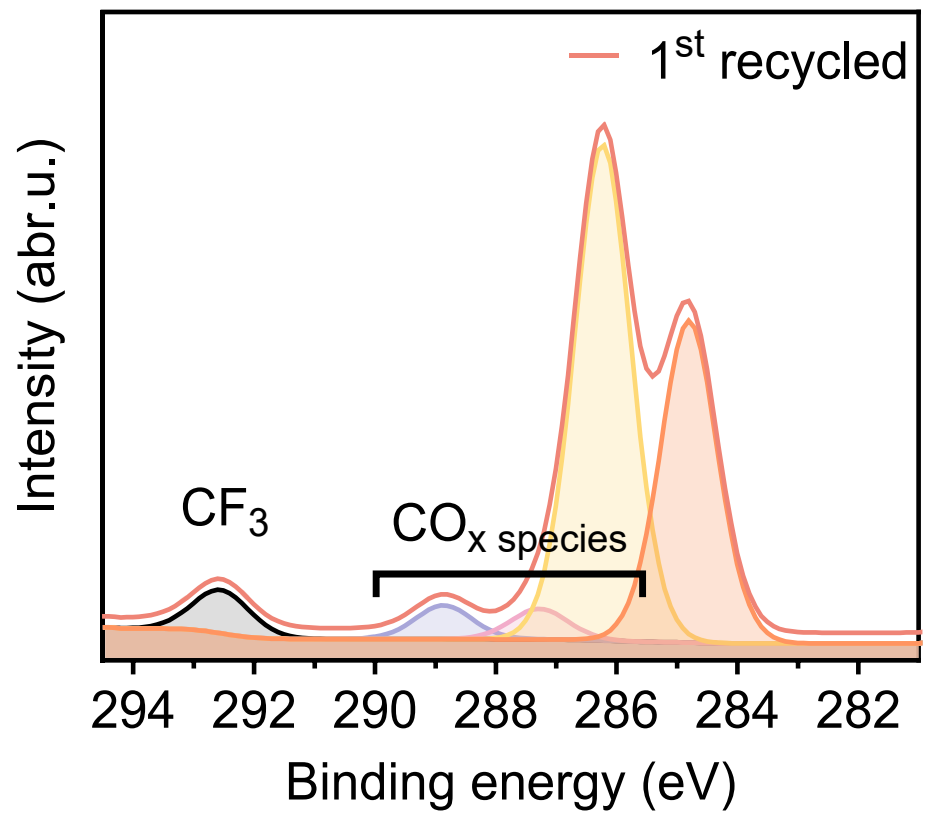

Figure S40. The surface C 1s XPS spectrum for 1st recycled SPE-65 membrane.

Table S6. Atomic concentration of XPS spectroscopy for pristine membrane and 1st recycled membrane, respectively.

Electron shell	Peak BE (eV)	Pristine membrane	1st recycled membrane
		Amount (at. %)	
C1s	284-293	56.8	60.1
O1s	532-534	21.64	21.84
N1s	399-402	4.54	4.11
F1s	688.57	9.56	9.03
S2p3	163-169	5.25	4.05
Si2p	102.16	2.21	0.88

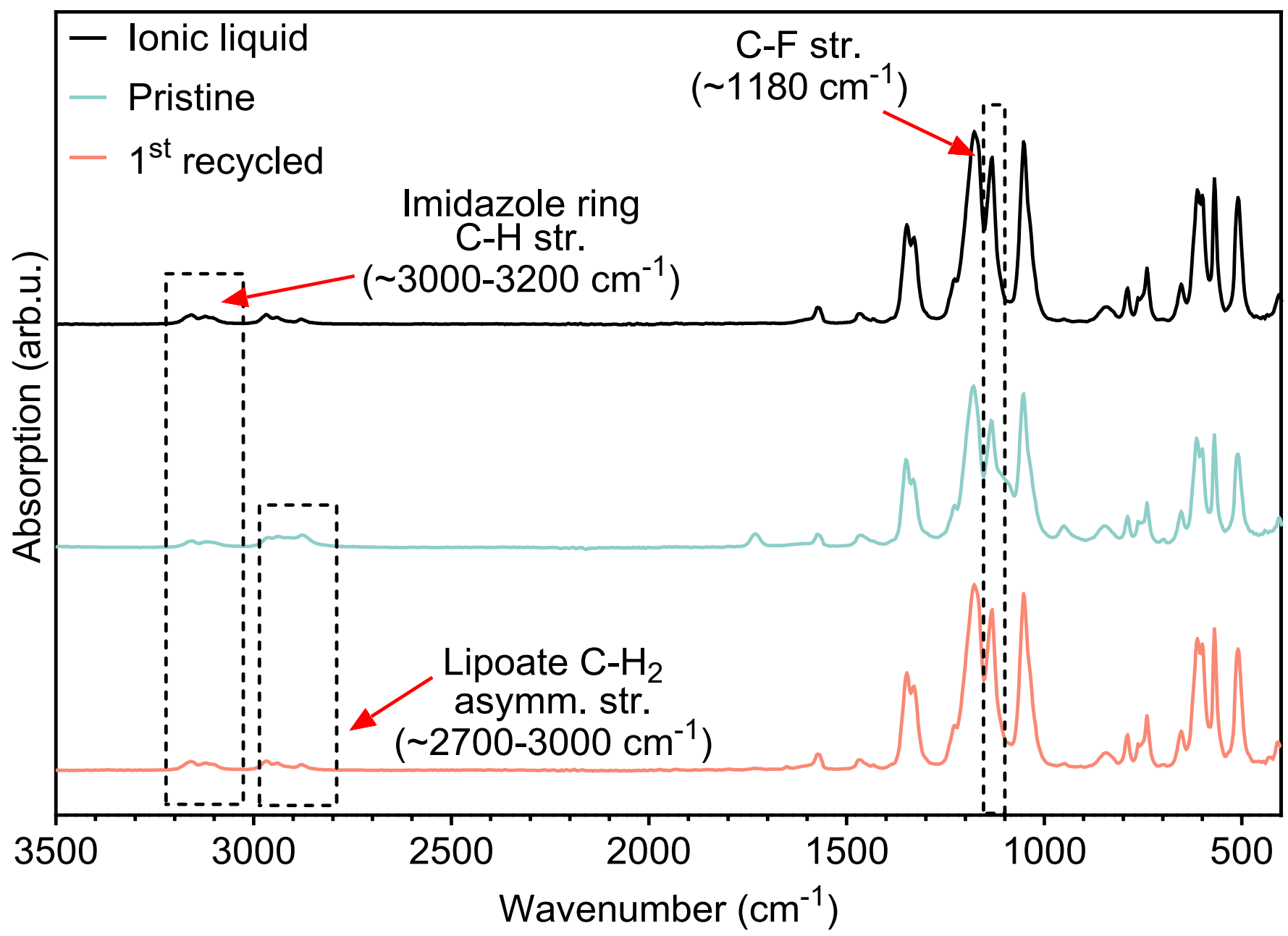

Figure S41. ATR-FTIR spectroscopy of ionic liquid, pristine SPE-65 and 1st recycled SPE-65, respectively. Note: The characteristic absorption bands of the ionic liquid were assigned based on previously reported[1], confirming the successful incorporation and retention of the ionic liquid within the SPE-65 membrane after recycling.

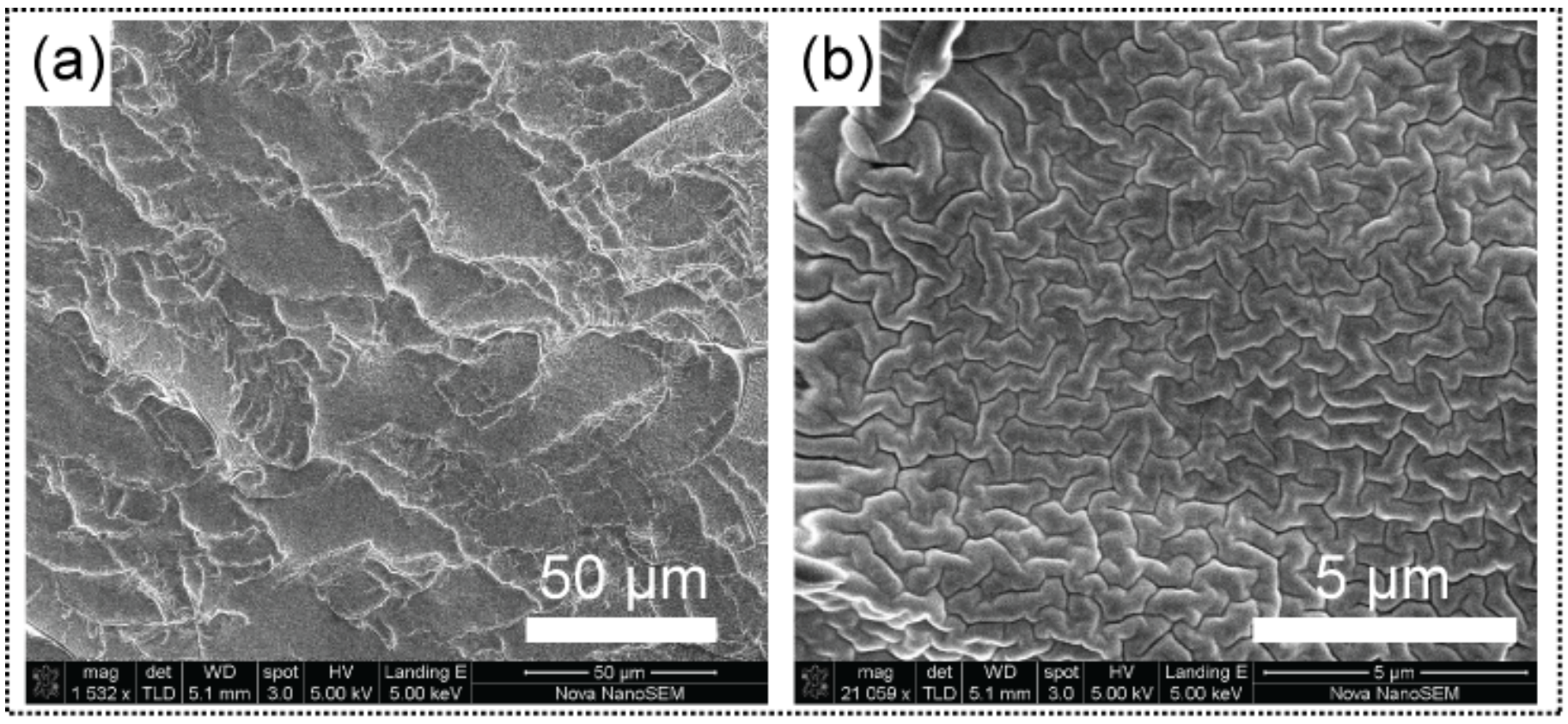

Figure S42. Cross-section SEM images for pristine SPE-65membranes.

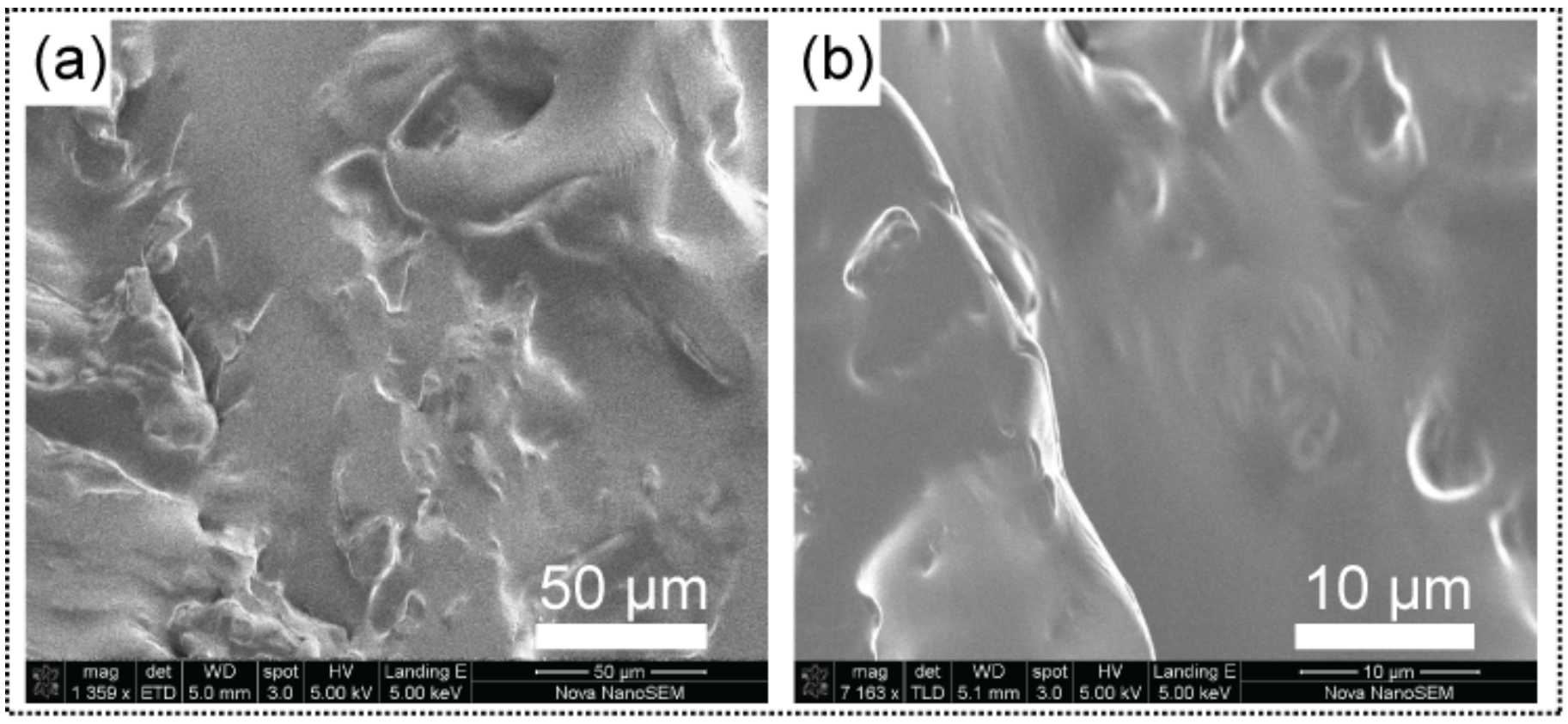

Figure S43. Cross-section SEM images for 1st recycled SPE-65membranes.

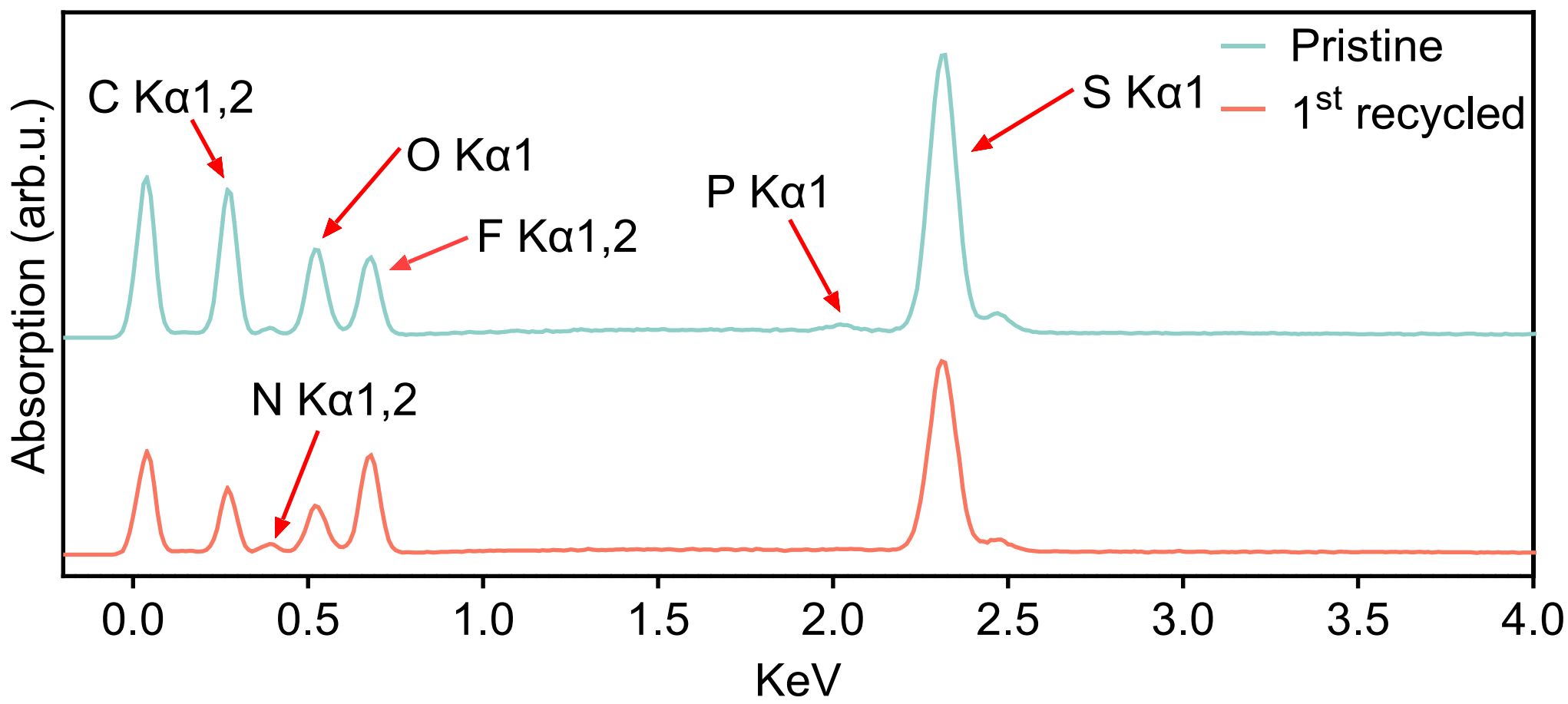

Figure S44. SEM-EDX mapping spectrum of a) pristine and b) 1st recycled membrane, confirming the presence of relevant elemental peaks attributed to ionic liquid (F, S, and N), polylipoates (O, S), and TPO (P).

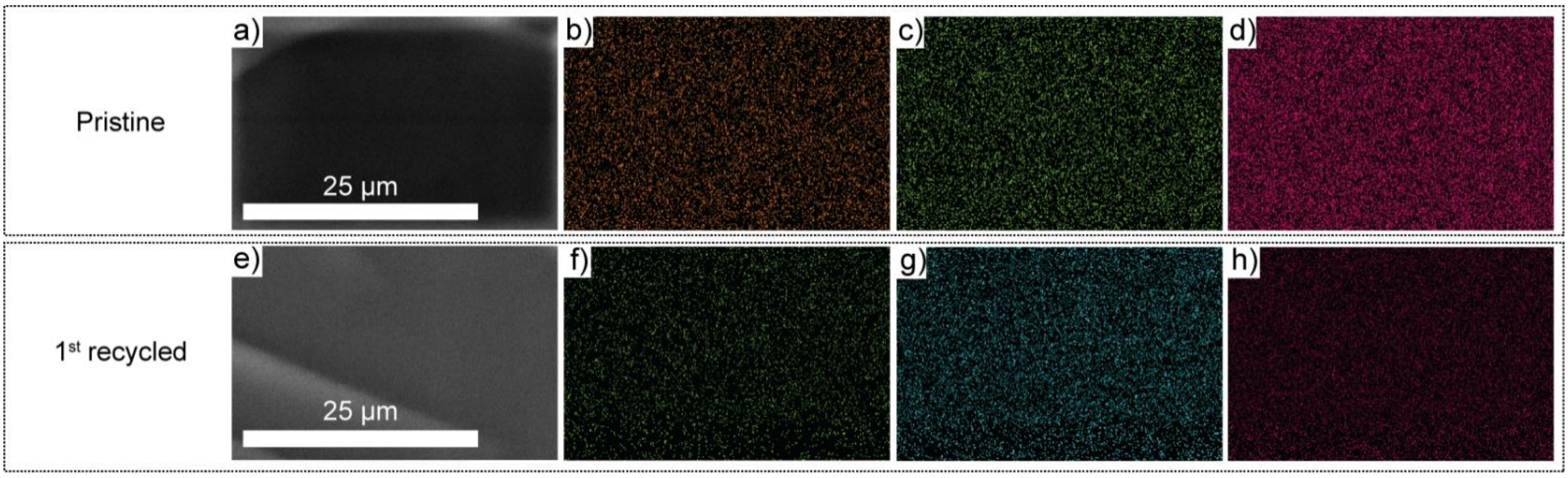

Figure S45. EDX experiments; a-d) SEM micrograph of pristine SPE-65, and elemental mapping of oxygen (O), fluorine (F), and sulfur (S) respectively; e-h) SEM micrograph of 1st recycled SPE-65, and elemental mapping of oxygen, fluorine, and sulfur, respectively.

Supporting Information - References

[1] K. Lee, Y. Shang, V. A. Bobrin, R. Kuchel, D. Kundu, N. Corrigan, C. Boyer, “3D Printing Nanostructured Solid Polymer Electrolytes with High Modulus and Conductivity” *Adv. Mater.* **2022**, *34*, 2204816.
